# RC1339/APRc from *Rickettsia conorii* Is a Novel Aspartic Protease with Properties of Retropepsin-Like Enzymes

**DOI:** 10.1371/journal.ppat.1004324

**Published:** 2014-08-21

**Authors:** Rui Cruz, Pitter Huesgen, Sean P. Riley, Alexander Wlodawer, Carlos Faro, Christopher M. Overall, Juan J. Martinez, Isaura Simões

**Affiliations:** 1 The Center for Neuroscience and Cell Biology (CNC), Coimbra, Portugal; 2 Biocant, Biotechnology Innovation Center, Cantanhede, Portugal; 3 Centre for Blood Research and Department of Biological and Medical Sciences, Faculty of Dentistry, University of British Columbia, Vancouver, British Columbia, Canada; 4 Vector-Borne Diseases Laboratories, Department of Pathobiological Sciences, School of Veterinary Medicine, Louisiana State University, Baton Rouge, Louisiana, United States of America; 5 Protein Structure Section, Macromolecular Crystallography Laboratory, National Cancer Institute at Frederick, Frederick, Maryland, United States of America; 6 Department of Biochemistry and Molecular Biology, University of British Columbia, Vancouver, British Columbia, Canada; Yale University School of Medicine, United States of America

## Abstract

Members of the species *Rickettsia* are obligate intracellular, gram-negative, arthropod-borne pathogens of humans and other mammals. The life-threatening character of diseases caused by many *Rickettsia* species and the lack of reliable protective vaccine against rickettsioses strengthens the importance of identifying new protein factors for the potential development of innovative therapeutic tools. Herein, we report the identification and characterization of a novel membrane-embedded retropepsin-like homologue, highly conserved in 55 *Rickettsia* genomes. Using *R. conorii* gene homologue RC1339 as our working model, we demonstrate that, despite the low overall sequence similarity to retropepsins, the gene product of *rc1339* APRc (for Aspartic Protease from *Rickettsia conorii*) is an active enzyme with features highly reminiscent of this family of aspartic proteases, such as autolytic activity impaired by mutation of the catalytic aspartate, accumulation in the dimeric form, optimal activity at pH 6, and inhibition by specific HIV-1 protease inhibitors. Moreover, specificity preferences determined by a high-throughput profiling approach confirmed common preferences between this novel rickettsial enzyme and other aspartic proteases, both retropepsins and pepsin-like. This is the first report on a retropepsin-like protease in gram-negative intracellular bacteria such as *Rickettsia*, contributing to the analysis of the evolutionary relationships between the two types of aspartic proteases. Additionally, we have also shown that APRc is transcribed and translated in *R. conorii* and *R. rickettsii* and is integrated into the outer membrane of both species. Finally, we demonstrated that APRc is sufficient to catalyze the *in vitro* processing of two conserved high molecular weight autotransporter adhesin/invasion proteins, Sca5/OmpB and Sca0/OmpA, thereby suggesting the participation of this enzyme in a relevant proteolytic pathway in rickettsial life-cycle. As a novel *bona fide* member of the retropepsin family of aspartic proteases, APRc emerges as an intriguing target for therapeutic intervention against fatal rickettsioses.

## Author Summary

Several rickettsiae are pathogenic to humans by causing severe infections, including epidemic typhus (*Rickettsia prowazekii*), Rocky Mountain spotted fever (*Rickettsia rickettsii*), and Mediterranean spotted fever (*Rickettsia conorii*). Progress in correlating rickettsial genes and gene functions has been greatly hampered by the intrinsic difficulty in working with these obligate intracellular bacteria, despite the increasing insights into the mechanisms of pathogenesis of and the immune response to rickettsioses. Therefore, comparison of the multiple available genomes of *Rickettsia* is proving to be the most practical method to identify new factors that may play a role in pathogenicity. Here, we identified and characterized a novel retropepsin-like enzyme, APRc, that is expressed by at least two pathogenic rickettsial species, *R. conorii* and *R. rickettsii*. We have also established that APRc acts to process two major surface antigen/virulence determinants (OmpB/Sca5, OmpA/Sca0) *in vitro* and we suggest that this processing event is important for protein function. We demonstrate that APRc is specifically inhibited by drugs clinically used to treat HIV infections, providing the exciting possibility of targeting this enzyme for therapeutic intervention. With this work, we demonstrate that retropepsin-type aspartic proteases are indeed present in prokaryotes, suggesting that these enzymes may represent an ancestral form of these proteases.

## Introduction

The genus *Rickettsia* represents a group of gram-negative obligate intracellular bacteria that exist as pathogens and symbionts of eukaryotic cells. These bacteria are transmitted to mammals by arthropod vectors such as ticks, lice, and fleas. With the advent of new molecular biology tools and whole genome sequence analysis *Rickettsia* species have been classified into several distinct genetic groups including the ancestral group (AG), spotted fever group (SFG), typhus group (TG), and transitional group (TRG) [1]–[4]. Many rickettsial species belonging to the TG and SFG are pathogenic to humans causing serious illnesses, including epidemic typhus (*Rickettsia prowazekii*), Rocky Mountain spotted fever (RMSF) (*Rickettsia rickettsii*), and Mediterranean spotted fever (MSF) (*Rickettsia conorii*) [5]–[7]. The life-threatening character of many rickettsial species is the consequence of their highly virulent properties and unique biological characteristics including aerosol transmission, persistence in infected hosts, and low infectious dose. There is growing concern about rickettsial diseases and their impact on global health, with members of the genus *Rickettsia* being identified, together with other bacteria, as emerging/re-emerging pathogens, responsible for the majority of emerging infectious diseases events between 1940 and 2004 [8]. Although in the U.S. the case fatality rate for RMSF has declined over the years (to less than 0.5% in 2010), the Brazilian spotted fever (also caused by *R. rickettsii*) has case fatality rates ranging from 30% to 80% [9]. MSF is also associated with high morbidity and mortality, with case fatality rates varying from 21% to 33% in Portugal [9]. In fact, from 1989 to 2000 the incidence rate of MSF in Portugal was one of the highest in the Mediterranean area (9.8 cases per million) [10]. From the typhus group, *R. prowazekii* infection is still recognized as one of the most severe rickettsioses with case fatality rates as high as 12%. The emergent and severe character of rickettsioses with their associated high morbidity and mortality rates, together with the lack of protective vaccines, strengthen the importance of identifying new protein factors that may work as potential targets for the development of more efficacious therapies against these diseases [6], [11].

In line with what has been described for other obligate intracellular bacteria, rickettsial species have highly conserved and reduced genome sizes, which derive from reduction of originally larger genomes accompanying the adaptation to strict intracellular lifestyles [12]–[15]. Significant progress has been made with regards to the analysis of genetic composition of a number of rickettsial species (now 55 sequenced genomes); however, the genetic intractability of these bacteria has severely limited molecular dissection of virulence factors associated with their intracellular parasitism and pathogenic mechanisms. Comparative genomics has resulted in identification of several genes encoding secreted proteins that are potential virulence factors involved in pathogenesis [16]–[18]. However, many more putative rickettsial virulence factors and hypothetical proteins remain to be functionally defined.

Bacterial pathogenicity generally results from a combination of factors and there are different bacterial components and strategies contributing to virulence [19]. Among these components emerges a diverse array of proteolytic enzymes (mainly localized to the bacterial surface or secreted), which have been recognized as virulence factors in several pathogenic bacteria. Such enzymes play critical functions related to colonization and evasion of host immune defenses, acquisition of nutrients for growth and proliferation, and facilitation of dissemination or tissue damage during infection [19]–[21]. The relevance of proteolytic events for bacterial pathogenicity and the progressive increase in antibiotic resistance among pathogenic bacteria contribute to positioning proteases as potential candidate targets for the development of alternative antibacterial strategies [20]. However, thus far only a few proteases have been identified and characterized in *Rickettsia* [18], [22], [23].

In this work, we have identified a gene coding for a putative membrane-embedded aspartic protease (AP) of the retropepsin type, conserved in all 55 sequenced *Rickettsia* genomes. The retropepsins (also known as family A2 of aspartic proteases) were first identified with the discovery of the Human Immunodeficiency Virus 1 (HIV-1) protease (PR) in the late 1980's [24] and the recognition of its essential role in the maturation of HIV-1. These proteases require homodimerization of two monomeric units in order to form a functional enzyme, structurally related to the pepsin family (A1) of bi-lobal APs [25]–[27]. Interestingly, the existence of retropepsin-like enzymes in prokaryotes has always been a matter of debate [28], [29] and never unequivocally demonstrated. Herein, we describe the characterization of the retropepsin-like homologue from *Rickettsia conorii* (RC1339/APRc – for Aspartic Protease from *Rickettsia conorii*) and demonstrate that this protease shares several enzymatic properties (e.g. autolytic activity, optimum pH, sensitivity to specific HIV-1 PR inhibitors) with other APs. Moreover, we demonstrate that this novel protease is expressed *in vivo* in two pathogenic species of *Rickettsia* and provide experimental evidence for its potential role as a modulator of rickettsial surface cell antigen (Sca) proteins OmpB (Sca5/rOmpB) and OmpA (Sca0/rOmpA).

This work provides the first evidence for a retropepsin-like protease in gram-negative intracellular bacteria such as *Rickettsia*, and the contribution of these results to change the paradigm on the evolutionary relationships between pepsin-like and retroviral APs is also discussed.

## Results

### The gene encoding a putative retropepsin-like enzyme is highly conserved in *Rickettsia* species

**Figure 1:**
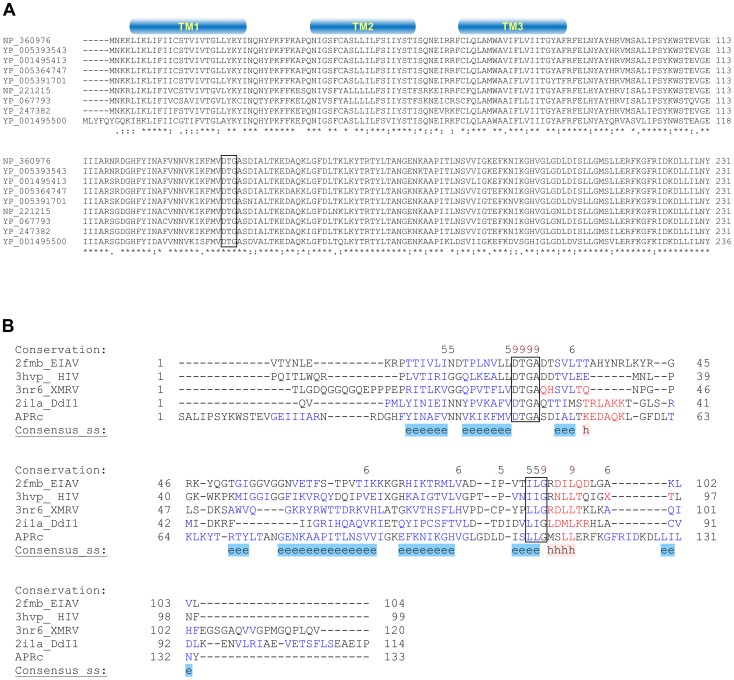
*RC1339/*APRc gene homologues from *Rickettsia* spp. display a striking pattern of sequence conservation among each other and retain structural similarity with other members of the retropepsin family. (A) Multi-alignment of deduced amino acid sequences of the putative retropepsin-like protease from representative species from all rickettsial taxonomic groups (spotted fever group, typhus group, transitional group and ancestral group). Sequences were aligned against RC1339/APRc sequence from *R. conorii* (NP_360976) using the ClustalW software [69]. Accession numbers and corresponding species are described in Table 1. The predicted α-helical transmembrane domains are represented by cylinders and the box indicates the active site motif (DTG). (B) Structure-based alignment of the soluble catalytic domain of RC1339/APRc with HIV-1 (PDB 3hvp), EIAV (PDB 2fmb) and XMRV (PDB 3nr6) retropepsins and with DdI1 putative protease domain (PDB 2i1a), performed with PROMALS3D [71]. The first line shows conservation indices for positions with a conservation index above 4. Consensus_ss represent consensus predicted secondary structures (alpha-helix: h; beta-strand: e). Sequences are colored according to predicted secondary structures (red: alpha-helix, blue: beta-strand). Red nines highlight the most conserved positions. Active site consensus motif Asp-Thr-Gly and hydrophobic-hydrophobic-Gly sequence are boxed.

*In silico* analysis of the genome sequence of *R. conorii* str. Malish 7, the etiologic agent of MSF, revealed a gene (RC1339) with 696 bp, encoding a putative retropepsin-like aspartic protease. This gene is highly conserved among 55 sequenced *Rickettsia* genomes, with deduced amino acid sequences sharing more than 84% sequence identity among each other. This striking pattern of conservation is illustrated in Figure 1A, which shows the alignment of the deduced amino acid sequence of *R. conorii* RC1339/APRc with eight other homologues from representative species of each rickettsial group (SFG, TG, TRG and AG). Protein sequences are highly conserved, with amino acid identities ranging from 84.0% (*R. bellii* str. OSU 85–389 APRc homologue) to 99.6% (*R. parkeri* str. Portsmouth APRc homologue) (Table 1). Strikingly, this novel type of rickettsial AP showed no detectable sequence homology when compared with APs from other organisms, except for the presence of the hallmark sequence motifs of family A2 members. Although the overall sequence identity with other retropepsins was found to be lower than 14% (with only 6% for the HIV-1 PR which is considered the archetypal member of this family of APs), it was possible to identify the active site consensus motif Asp-Thr-Gly (contained in the sequence Xaa-Xaa-Asp-Xbb-Gly-Xcc, where a Xaa is hydrophobic, Xbb is Thr or Ser, and Xcc is Ser, Thr or Ala) corresponding to the sequence Met-Val-Asp-Thr-Gly-Ala (amino acids 138–143), followed downstream by a hydrophobic-hydrophobic-Gly sequence (Leu-Leu-Gly, amino acids 208–210). These features are characteristic of retropepsin-like APs which are obligate homodimers, with each monomer contributing one catalytic residue and one hydrophobic-hydrophobic-Gly motif to form the structural feature known as psi loop [25], [26]. A distinguishing feature of APRcs compared to retroviral enzymes is their predicted membrane-embedded nature, with different algorithms predicting three putative transmembrane (TM) α-helix segments in the N-terminal domain of APRc (Figure 1A). The presence of cysteine residues in these predicted transmembrane regions, which may be linking the three α-helical chains together through interchain disulfide bonds, may likely contribute to structural stability. Additionally, an inside orientation for the N terminus and an outside orientation for the C-terminal soluble protease domain of APRc (Arg87-Tyr231) relative to the membrane was predicted by the HMMTOP2 [30]. Interestingly, a similar domain organization - putatively membrane embedded with a soluble catalytic domain - with variations in the number of predicted TM helices has been also described for eukaryotic retropepsin-like proteases such as human and mouse SASPase [31], [32], as well as for one putative retroviral-type AP (SpoIIGA) [33] identified in *Bacillus subtilis*, for which no enzymatic characterization is yet available.

**Table 1:** Protein sequence identity of each APRc homologue in relation to NP_360976 as well as the taxonomic group of analyzed rickettsial spp.

APRc HOMOLOGUES	SPECIES	IDENTITY	TAXONOMY
***NP_360976***	*R. conorii* str. Malish 7	100%	SFG
***YP_005393543***	*R. parkeri* str. Portsmouth	99.6%	SFG
***YP_001495413***	*R. rickettsii* str. Sheila Smith	99.1%	SFG
***YP_005364747***	*R. amblyomii* str. GAT-30V	97.4%	SFG
***YP_005391701***	*R. montanensis* str. OSU 85–930	96.5%	SFG
***NP_221215***	*R. prowazekii* str. Madrid E	90.0%	TG
***YP_067793***	*R. typhi* str. Wilmington	88.3%	TG
***YP_247382***	*R. felis* str. URRWXCal2	95.2%	TRG
***YP_001495500***	*R. bellii* str. OSU 85–389	84.0%	AG

(SFG: spotted fever group; TG: typhus group; TRG: transitional group; AG: ancestral group).

Despite the high divergence at the sequence level, a structure-based alignment of the putative soluble catalytic domain of RC1339/APRc with HIV, Equine Infectious Anemia Virus (EIAV), and Xenotropic Murine Leukemia Virus-related Virus (XMRV) retropepsins, as well as with Ddi1 putative protease domain (Figure 1B), further suggested an overall retention of structural similarity through conservation of secondary structure, thereby anticipating an evolutionary relationship between APRc and retropepsins.

### RC1339/APRc displays autoprocessing activity strictly dependent on the catalytic aspartate residue

**Figure 2:**
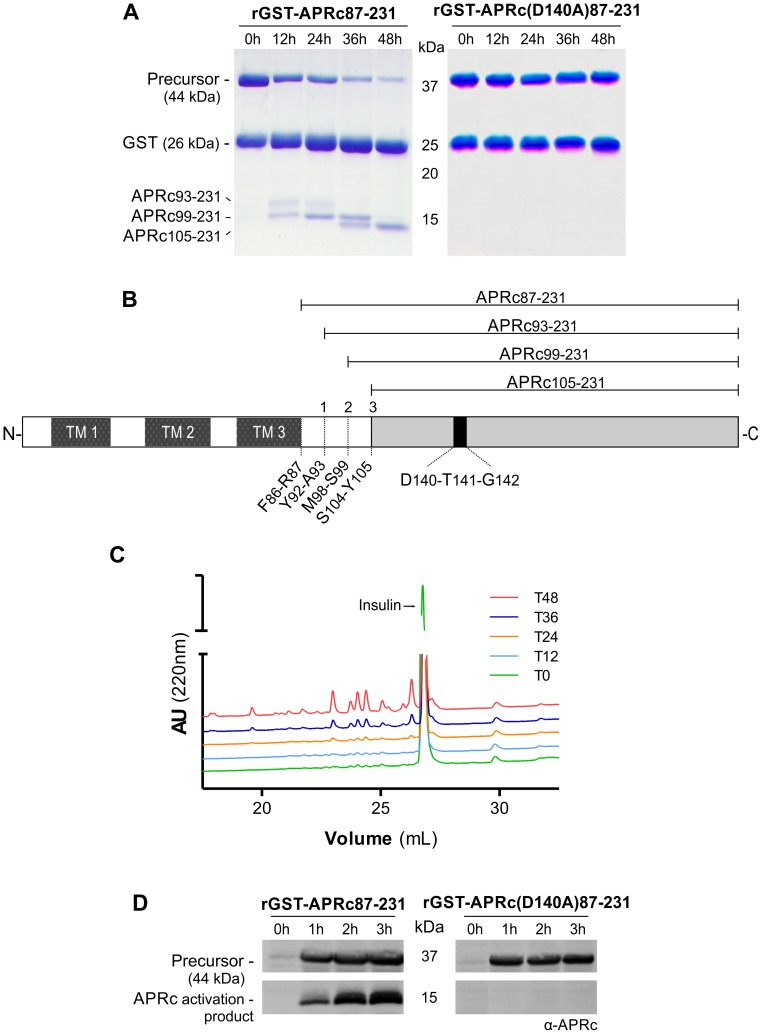
The recombinant soluble catalytic domain of APRc displays autoprocessing activity dependent on the catalytic aspartate residue. (A) The soluble catalytic domain (amino acids 87–231) was fused to GST and produced in *E. coli*. Upon purification, the auto-activation of rGST-APRc_87–231_ was performed *in vitro* in 0.1 M sodium acetate buffer pH 6 at 37°C for 48 h and monitored by SDS-PAGE stained with Coomassie blue. rGST-APRc_87–231_ undergoes multi-step auto-activation processing, resulting in the formation of the activated form APRc_105–231_ with ∼14.2 kDa (left panel). Mutation of the active site aspartic acid by alanine in this fusion construct [rGST-APRc(D140A)_87–231_] clearly impaired the auto-catalytic activity of the protease (right panel). (B) Schematic representation of full-length APRc domain organization. APRc is predicted to comprise three transmembrane domains (TM 1–3) at the N terminus and the soluble catalytic domain at the C terminus. The three auto-cleavage sites (shown in A) identified by Edman degradation are highlighted by order of cleavage (1–3). (C) Activity of wt rGST-APRc_87–231_ towards oxidized insulin B chain was tested over activation time. Samples from each time point evaluated in panel A were incubated with oxidized insulin B chain. Reaction products were then evaluated by RP-HPLC showing that substrate cleavage (appearance of four major peaks) was concomitant with appearance of the final activation product. T0, T12, T24, T36 and T48, correspond to the different time points of activation (hours) tested, as shown in A. (D) rGST-APRc_87–231_ auto-processing ability during expression was evaluated in total lysates of *E. coli* cells expressing wt rGST-APRc_87–231_ or the correspondent active site mutant rGST-APRc(D140A)_87–231_ over a time-course of 3 h and subsequently subjected to Western blot analysis with anti-APRc antibody. A band with approximately 15 kDa was only detected for the wt construct. Incubation and expression time course in hours (h) are indicated above gels and the molecular weight markers in kilodaltons (kDa) are shown on the left.

Using *R. conorii* RC1339 as our working model, we started assessing, by producing the soluble catalytic domain fused to GST in *E. coli*, whether this gene would indeed encode an active aspartic protease. Assuming the predicted boundary between the transmembrane and soluble catalytic domains at Phe86-Arg87, the synthetic codon optimized sequence coding for the whole soluble domain was cloned into pGEX-4T2 (pGST-APRc_87–231_) and the fusion construct was expressed in *E. coli* (BL21 Star (DE3) strain). The soluble fraction of the cell lysates was subjected to a GSTrap HP affinity chromatography and the eluted fractions were pooled and further purified by size-exclusion chromatography on a Superdex 200 HiLoad 26/60. Purified fractions analyzed by SDS-PAGE confirmed the presence of the fusion protein with the molecular mass of approximately 42 kDa, as well as free GST (25 kDa), which likely results from proteolytic degradation by the host. One of the features shared by the retropepsins is their autoprocessing activity which promotes their own release from a larger polyprotein precursor [25]. As shown in Figure 2A, our results demonstrate that recombinant rGST-APRc_87–231_ also undergoes a multi-step processing *in vitro* upon incubation at pH 6.0, resulting in the generation of different cleavage products over activation time. Edman sequencing of these APRc fragments allowed the identification of the three autolytic cleavage sites: Tyr92-Ala93, Met98-Ser99, and Ser104-Tyr105 (Figure 2B).

As a first approach to assess enzyme activity, we used oxidized insulin B chain as a substrate as this polypeptide is usually cleaved by aspartic proteases, and tested its cleavage over activation time for purified rGST-APRc_87–231_. As illustrated in Figure 2C, samples from each time point (0, 12, 24, 36 and 48 h) were tested and the reaction products separated by RP-HPLC. Interestingly, the presence of several insulin cleavage products was concomitant with the appearance of the activation product APRc_105–231_, suggesting that autoprocessing may be an essential step for the activation of recombinant APRc.

In order to evaluate the role of the putative catalytic aspartate for this autoprocessing activity, an active site mutant of rGST-APRc_87–231_, where the putative catalytic aspartate residue was mutated to an alanine [rGST-APRc(D140A)_87–231_] was produced, purified, and activated under the same conditions as for the WT fusion protein. As predicted, the mutation significantly affected the activation process (Figure 2A, right panel), suggesting that APRc is dependent on the conserved catalytic aspartate residue for triggering autolytic activity. To confirm that the previously observed insulin degradation resulted from APRc activity, parallel tests were performed with the active site mutant rGST-APRc(D140A)_87–231_ incubated under similar conditions (Figure S1). The autoprocessing ability of APRc and the importance of the catalytic aspartate were further confirmed by expressing the constructs harboring the soluble domain (rGST-APRc_87–231_) and its active site mutant (rGST-APRc(D140A)_87–231_) in *E. coli* and by analyzing total soluble fractions for the presence of APRc-activated forms with a specific APRc polyclonal antibody (raised towards amino acids 165–178). As shown in Figure 2D, and consistent with the results obtained in our *in vitro* assays, the activation products were only detected when the WT sequence was expressed, further corroborating the role of the catalytic aspartate in autoprocessing. This intrinsic autoprocessing observed during expression in *E. coli* is in line with what has been documented for other retropepsins (e.g. HIV-1 PR and XMRV PR [34], [35]).

**Figure 3:**
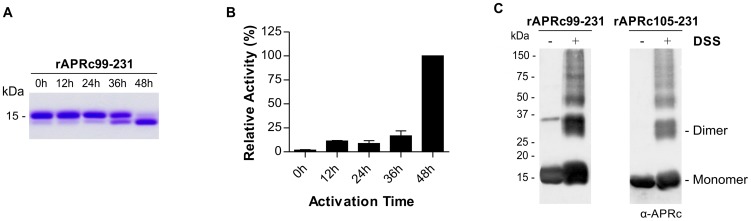
Auto-processing activity of the last intermediate of activation rAPRc_99–231_. (A) The intermediate of activation APRc_99–231_ was fused to C-terminal His-tag and produced in *E. coli*. Upon purification, the auto-activation assays were performed *in vitro* in 0.1 M sodium acetate buffer pH 6 at 37°C for 48 h and monitored by SDS-PAGE stained with Coomassie blue. rAPRc_99–231_ undergoes auto-processing, resulting in the formation of the activated form. (B) Activity of rAPRc_99–231_ towards the fluorogenic substrate MCA-Lys-Ala-Leu-Ile-Pro-Ser-Tyr-Lys-Trp-Ser-Lys-DNP was tested over activation time. Substrate cleavage increased with accumulation of the final activation product. The error bars represent standard deviation of the mean. (C) The quaternary configuration of rAPRc_99–231_ precursor and activated forms was assessed by incubating the protease with the cross-linker DSS. Both DSS treated and untreated protein samples were subjected to Western blot analysis with anti-APRc antibody. In the presence of the cross-linking agent, a significant proportion of the protein migrated as a dimer, although the monomeric forms and larger aggregates were also observed. The molecular weight markers in kilodaltons (kDa) are shown on the left.

Since the expression of the soluble domain of APRc fused to GST resulted in a high degree of contamination with free GST, an alternative strategy was undertaken to streamline the production of APRc activation product with higher yield and purity. For this we designed two new constructs where the sequences encoding the intermediate of activation APRc_99–231_, as well as the final product APRc_105–231_, were cloned into pET23a expression vector (Invitrogen) in frame with a C-terminal 6×His-tag. Both constructs were readily expressed in the soluble form in *E. coli*. A purification protocol consisting of a Ni-IMAC step, followed by dialysis of APRc-enriched polled fractions, and further purification through a cation exchange chromatography with a Mono S column was optimized. As shown in Figure 3A, the His-tagged intermediate rAPRc_99–231_ was also able to undergo auto-activation into the mature form at pH 6.0. To further characterize the enzymatic activity of APRc we designed a specific fluorogenic substrate, which mimics the identified auto-cleavage site between Ser104-Tyr105 residues (Rick14 peptide: MCA-Lys-Ala-Leu-Ile-Pro-Ser-Tyr-Lys-Trp-Ser-Lys-DNP), and tested this substrate during rAPRc_99–231_ activation. As previously observed with the GST-fusion precursor, activity towards this substrate was shown to be also dependent on the conversion step and the highest activity was observed upon accumulation of the conversion product (Figure 3A–B), further strengthening the importance of enzyme activation. Interestingly, when the final product APRc_105–231_ was directly produced in *E. coli*, no proteolytic activity was observed towards the same substrate (data not shown). This result suggests that protease autoprocessing may indeed be accompanied by some conformational change that is not observed when the activation product is directly expressed in *E. coli*. Wan and co-workers have reported a similar result for HIV-1 PR by showing that a recombinant protein corresponding to the mature form of the protease (99 amino acids) with two additional amino-acids at the N-terminus (Met and Gly) displayed no proteolytic activity [36]. Based on this result, we have focused on the construct of the precursor form APRc_99–231_ for further analysis.

Given the observed impact of mutating the catalytic Asp residue on the autoprocessing ability of APRc we decided to evaluate the effect of pepstatin (a classical inhibitor of aspartic proteases) and indinavir (an HIV-1 PR inhibitor) in rAPRc_99–231_ autoprocessing. Our results (Figure S2A) show that in the presence of pepstatin the auto-activation step was slightly slowed, whereas indinavir had no apparent inhibitory effect on this auto-processing activity. Surprisingly, EDTA inhibited rAPRc_99–231_ auto-activation, suggesting that a metal ion may be involved in proper folding and/or enzyme activity.

Given the homodimeric nature of retropepsins, crosslinking studies using DSS as the crosslinking agent were also conducted with purified rAPRc_99–231_, as well as with the derived activation product, in order to provide an evidence for APRc dimer formation. Reaction products and control samples were analyzed by immunoblotting (Figure 3D) and, as expected, the results revealed a significant amount of APRc associated as dimer, although monomeric and larger aggregate species were also visible. Similar results were observed when glutaraldehyde, which differs from DSS in the length of connecting backbone (11.4 Å for DSS and 7 Å for glutaraldehyde) was used (Figure S2B). These results were consistent with the analysis of both forms by analytical size-exclusion chromatography under native conditions (Figure S2C) where the presence of oligomeric species was observed, although the large majority of the protein was shown to accumulate as a monomer.

### The soluble domain of RC1339/APRc is an active enzyme with properties resembling those of retropepsins

**Figure 4:**
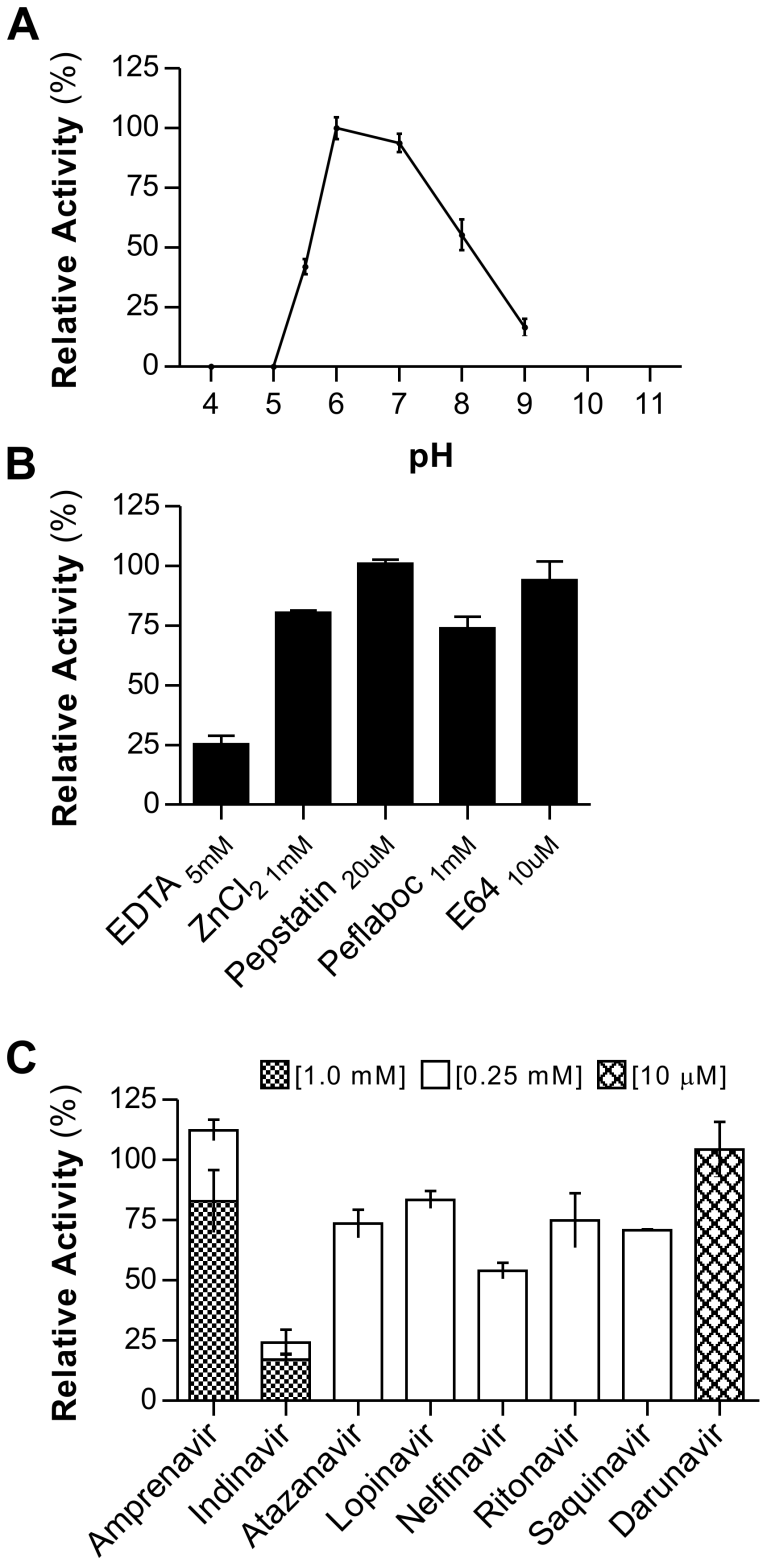
rAPRc activation product displays optimal activity at pH 6 and is strongly inhibited by specific HIV-1 PR inhibitors. The effect of pH, class-specific and HIV-1 PR specific inhibitors on the proteolytic activity of rAPRc activation product was evaluated using the synthetic fluorogenic substrate (MCA)Lys-Ala-Leu-Ile-Pro-Ser-Tyr-Lys-Trp-Ser-Lys(DNP). (A) Activity at different pH values. Activated rAPRc was incubated with the substrate at 37°C in buffers ranging between pH 4 and pH 9 containing 100 mM NaCl (50 mM sodium acetate pH 4.0, 5.0, 5.5 and 6.0 and 50 mM Tris-HCl pH 7.0, 8.0 and 9.0). (B) and (C) To test the effect different compounds, the protease was pre-incubated in the presence of each inhibitor for 10 minutes at room temperature in 50 mM sodium acetate pH 6.0 containing 100 mM NaCl before adding the substrate. The rate of substrate hydrolysis (RFU/sec) was monitored for 3 hours and the relative activity normalized by setting the maximum activity at 100%. The error bars represent standard deviation of the mean.

Based on the observed enzymatic activity upon conversion of the precursor form rAPRc_99–231_, all characterization studies were focused exclusively on this derived activation product (for simplification denoted APRc). The effect of pH was determined using the same fluorogenic substrate - MCA-Lys-Ala-Leu-Ile-Pro-Ser-Tyr-Lys-Trp-Ser-Lys-DNP - in a range of pH values from 4 to 9. From this analysis an optimal activity at pH 6.0 was observed (Figure 4A), with no appreciable hydrolytic activity below pH 5.0. This higher optimal pH value is consistent with optimum pH values reported for other retropepsins [37], [38].

When investigating the susceptibility of APRc to classical protease inhibitors (Figure 4B), this protease was shown to be insensitive to pepstatin A, even though a slightly inhibitory effect was observed during autolytic processing. In contrast, APRc activity was strongly inhibited by EDTA, retaining only 25% activity, and a small inhibitory effect was also observed with Pefabloc. No significant effect was observed after incubation with E-64, whereas incubation with Zn^2+^ (Figure 4B) slightly affected enzyme activity.

In order to provide additional evidence that APRc is indeed a retropepsin-like enzyme we analyzed the effects of different clinical inhibitors of HIV-1 PR. Strikingly, incubation with indinavir resulted in a near complete inhibition of APRc, even when tested at a final concentration of 0.25 mM in the assay. Additionally, nelfinavir, saquinavir, amprenavir and atazanavir also had a significant inhibitory effect, ranging between approximately 30–50% of inhibition (Figure 4C). This inhibitory effect of specific HIV-1 protease inhibitors against a prokaryotic retropepsin-like enzyme has not been previously described.

### APRc displays a unique specificity pattern with amino acid preferences resembling those of retropepsin- and pepsin-like PRs

To determine the APRc sequence specificity we employed Proteomic Identification of Protease Cleavage Sites (PICS) [39], [40], a high-throughput profiling approach based on the use of database-searchable proteome-derived oligopeptide libraries representing the natural biological sequence diversity as test substrates [39]. A unique advantage of PICS is the simultaneous determination of sequence specificity on both sides of the scissile bond, the prime-side (P′) and non-prime side (P) [41]. PICS substrate peptide libraries are prepared by digestion of a complex proteome from a sequenced model organism with highly specific endoproteases such as trypsin (cleavage after Arg and Lys) or GluC (cleavage after Glu and Asp), followed by blocking of primary amines at peptide N termini and in Lys side chains. Cleavage of library peptides by the test protease of interest generates C-terminal cleavage products with free α- amines that are exploited for selective enrichment and identification by LC-MS/MS. The identified cleavage products constitute the prime side sequences (P′) of the cleaved library peptides. Due to the non-random nature of the PICS libraries the non-prime side sequences can be inferred by database matching to allow reconstruction of the complete cleavage sites.

**Figure 5:**
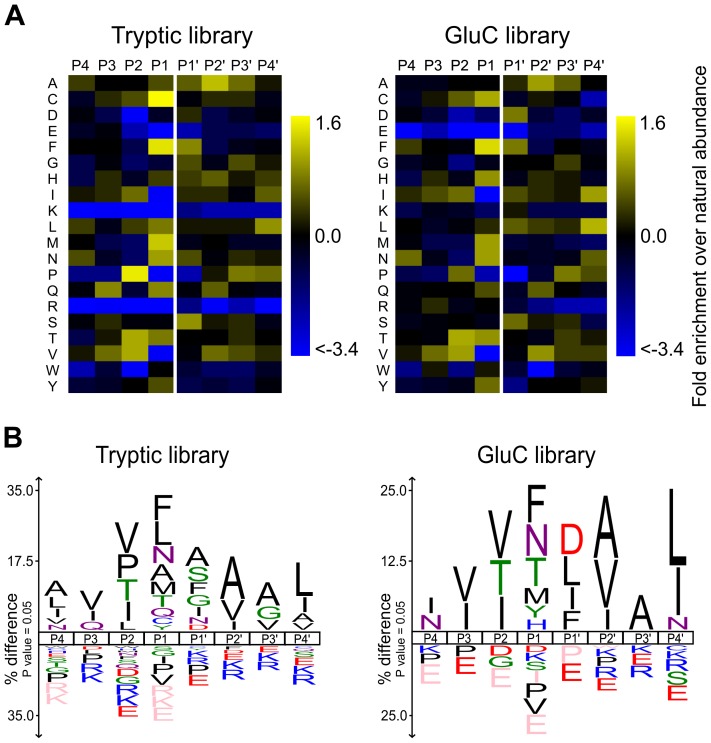
rAPRc specificity profiling reveals similar amino acid preferences to both retropepsin and pepsin-like proteases. Graphic representation of APRc specificity profile by Heatmaps and IceLogos. Results are from Tryptic and GluC peptide libraries derived from a *Homo sapiens* proteome (THP1 cells) incubated with activated rAPRc at a ratio of 1∶50 (enzyme/library). The analytical strategy applied was similar to that described in [40]. PICS libraries were analyzed by multiple sequence alignments and applying correction for natural amino acid abundance. For each class of PICS library, the average amino acid occurrences in P4–P4′ were calculated from three experiments and are either shown in the form of (A) a two-dimensional heatmap of log(2) transformed values of fold-enrichment over natural abundance of amino acids and (B) % difference IceLogos. Both tryptic and GluC display consistency between them. In IceLogos representation, horizontal axis represents the amino acid position and vertical axis denotes the over- and under-representation of amino acid occurrence compared with the Swiss-Prot *Homo sapiens* protein database. Cysteines are carboxyamidomethylated and lysines are dimethylated.

In this work, active APRc was incubated with PICS libraries generated by digestion of total human THP1 (human monocytic leukemia cell line) cell proteins by either trypsin or GluC. These PICS experiments resulted in the identification of 830 and 327 C-terminal cleavage products from tryptic and GluC libraries, respectively (Tables S1 and S2). The corresponding N-terminal cleavage products and complete cleavage sites were obtained and summarized using the WebPICS tool [40]. The PICS-based APRc specificity profiles are shown in Figure 5 and a good agreement was observed between the two complementary peptide libraries. APRc displays only moderate specificity and accepts multiple amino acids at each position. At P1, directly preceding the scissile bond, APRc showed a preference for large hydrophobic residues such as phenylalanine, tyrosine, methionine, leucine, and carboxyamidomethylated cysteine (modified during library preparation). In addition, APRc also preferred the neutral amino acids threonine and asparagine at this site. A similar preference was observed for P1′, although this further included small amino acids alanine, serine, and glycine, as well as aspartate. Notably, cleavage sites were almost devoid of Pro at P1 and P1′. Furthermore, PICS revealed distinct preferences for selected amino acids at other positions, likely reflecting structural constraints imposed by the substrate recognition and binding to the pocket site. In P2, APRc preferences include valine, isoleucine, proline, and threonine, whereas a predominant preference for small and branched aliphatic amino acids alanine, valine, and isoleucine was observed at P2′. More distant from the cleavage site, small preferences for valine and isoleucine at P3 and for alanine and glycine in P3′ and a strong preference for leucine or isoleucine at P4′ were observed. Interestingly, basic and acidic residues were significantly underrepresented throughout. The large number of APRc cleavage sites identified from the tryptic PICS library further allowed investigation of subsite cooperativity. When comparing two of the strongest cleavage site determinants, phenylalanine at P1 and proline at P2, we observed apparent mutual exclusion. Of the 103 unique cleavage sites that contained proline in P2, only 4 had phenylalanine in P1 (3.7% compared to 10.5% occurrence for all identified cleavage sites), which was compensated by more frequent occurrence of P1 methionine (10.3% compared to 5.8% total occurrence) and P1 asparagine (14% compared to 8.3% total occurrence). Correspondingly, peptides with phenylalanine in P1 yielded 4.6% P2 proline (compared with 12.9% total occurrence), whereas peptides with methionine or asparagine in P1 yielded 22.9% or 21.7% P2 proline, respectively. A similar trend was observed in identified cleavage sites from GluC libraries, indicating subsite cooperativity between P2 and P1.

These results clearly show that, although displaying a unique profile, APRc shares some specificity requirements with retropepsins as well as with pepsin-like enzymes (particularly BACE), further supporting APRc has being a member of the aspartic protease family.

### RC1339/APRc accumulates in the outer membrane in *E. coli*

**Figure 6:**
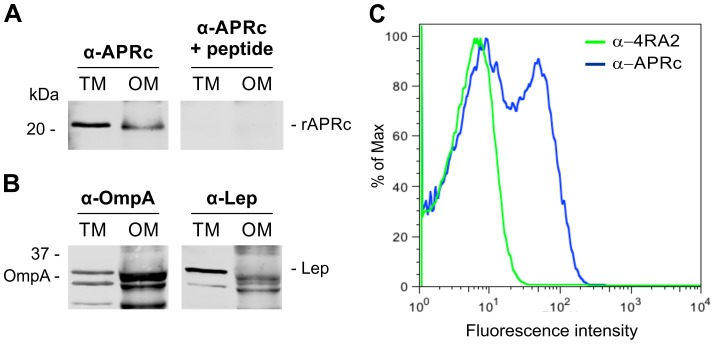
Recombinant full-length APRc accumulates in the outer membrane in *E. coli* and the soluble catalytic domain is exposed to the cell surface. (A) Full-length APRc was expressed in *E. coli* and total (TM) as well as outer membrane (OM) fractions were isolated and analyzed by Western blot with anti-APRc antibody (left panel). As a control for non-specific staining, peptide competition assays were performed by blocking the anti-APRc antibody with the immunizing peptide (right panel). One specific band with approximately 21 kDa was detected in the OM fraction. (B) The purity of OM fractions was confirmed by using OmpA and Lep proteins as internal markers for the outer and inner membranes of *E. coli*, respectively. Both proteins were present in TM faction, while only OmpA is detected in OM faction. (C) Flow cytometric analysis was carried out for recombinant APRc recognition at the surface of *E. coli* cells. PFA-fixed *E. coli* cells were incubated with anti-APRc and anti-RNA polymerase α (RNAPα mAb 4RA2,) followed by secondary detection using goat anti-rabbit IgG Alexa Fluor 488- and goat anti-mouse IgG R-PE-Cy5.5 conjugated secondary antibodies, respectively. Porous, permeabilized cells staining positive for RNAPα were excluded from the analysis by selective gating of this population. Fluorescence was detected on the remaining *E. coli* cells incubated with anti-APRc, thereby confirming the expression of recombinant APRc at the outer membrane and its exposure to extracellular milieu.

As previously mentioned, full-length RC1339/APRc was predicted to be membrane-embedded with an extracytoplasmic orientation of the C-terminal domain. In order to provide experimental validation of these theoretical observations we used *E. coli* as our working model. An untagged construct in pET28a comprising RC1339/APRc full-length coding sequence was generated and protein expression carried out as described under Experimental Procedures. Protease insertion into the membrane was first assessed by subcellular fractionation studies followed by Western blot analysis with a specific APRc antibody. A band of approximately 21 kDa, whose nature was confirmed by peptide competition assays, was detected in the total membrane fraction and shown to accumulate in the outer membrane (Figure 6A). The purity of the outer membrane (OM) fraction was confirmed by Western blotting against *E. coli* Lep and OmpA proteins, as inner and outer membrane markers [42], respectively, and compared to the total membrane fraction (Figure 6B). As expected, OmpA was detected in the outer membrane fraction and the absence of cross-contamination with inner membrane proteins was confirmed through loss of signal for Lep, when compared with total membrane fraction. Interestingly, APRc displayed a molecular weight lower than expected (∼21 kDa instead of the predicted 26.4 kDa), and parallel experiments with a C-terminal His-tagged construct confirmed the presence of the tag in the membrane fractions (data not shown), clearly suggesting that the protease may be processed at the N terminus during translocation to the membrane.

In an attempt to expand our knowledge about the membrane topology of APRc, further studies were performed in order to determine the overall in/out orientation of this protein relative to the outer membrane of *E. coli*. To investigate this, PFA-fixed *E. coli* cells expressing untagged full-length APRc were subjected to flow cytometry with both anti-APRc and anti-α-subunit of RNA polymerase (mAb 4RA2) antibodies. The staining of *E. coli* cells with the 4RA2 mAb was primarily used to restrict the analysis to the non-permeable cells. As shown in Figure 6C, after gating out all the cells that stained positive for RNAPαprotein (permeable cells), bacterial surface staining with anti-APRc was observed, confirming the integration of RC1339/APRc into the outer membrane of *E. coli* and the orientation of the soluble catalytic domain to the extracellular milieu.

### APRc is expressed in *Rickettsia conorii* and *Rickettsia rickettsii* and is localized in the outer membrane

**Figure 7:**
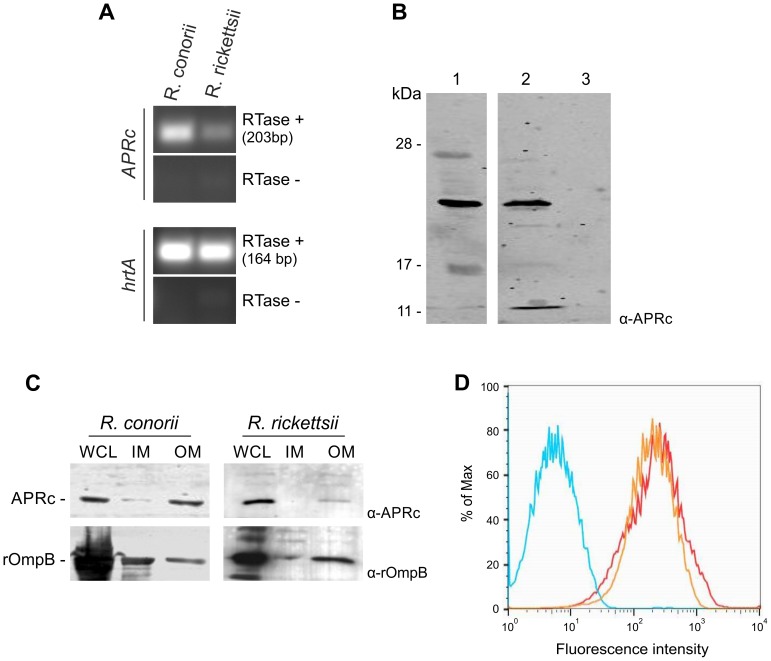
*RC1339/*APRc is expressed in *Rickettsia conorii* and *Rickettsia rickettsii* and accumulates at the outer membrane in both species. (A) RT-PCR analysis of *RC1339/*APRc expression on rickettsial spp.. The housekeeping gene *hrtA* (17 kDa surface antigen) was used as a control. The negative control for the cDNA synthesis lacking reverse transcriptase is identified by (RTase -). Rickettsial species are identified on the top and the gene names are shown on the left side of the agarose gel. (B) A whole cell lysate from *R. rickettsii* (1) and insoluble (2) and soluble (3) fractions from *R. conorii* extracts were isolated and then subjected to Western Blot analysis with anti-APRc antibody. A specific band with approximately 21 kDa was detected. (C) Whole cell lysates (WCL), inner (IM) and outer membrane (OM) fractions from sarkosyl treatment of *R. rickettsii* and *R. conorii* extracts were isolated and then subjected to Western Blot analysis with anti-APRc and anti-rOmpB antibody. APRc shares the same localization of rOmpB, an internal marker for outer membrane of *Rickettsia* spp. Molecular mass markers in kilodaltons (kDa) are shown on the left. (D) For flow cytometric analysis of APRc expression in *R. conorii*, fixed bacteria were queried for deposition of anti-APRc (orange trace), negative control lacking primay antibody (blue trace), or the positive control anti-OmpB (red trace), a known rickettsial surface protein. After incubation with fluorescent secondary antibody, both anti-APRc and anti-OmpB detected on the bacterial surface (increased fluorescence), indicating accessibility of these target proteins to exogenously applied antibody.

To determine whether *RC1339*/APRc and the *R. rickettsii* homologue are expressed in the context of the intact bacterium, we isolated total RNA from *R. conorii* and *R. rickettsii* grown in Vero cells and performed reverse transcriptase PCR (RT-PCR). As shown in Figure 7A, both *R. conorii* and *R. rickettsii* produce transcripts for *rc1339* and A1G_07330, respectively, when grown in culture. To confirm expression of these transcripts, protein lysates from each rickettsial species were separated by SDS-PAGE and immunoblotting analyses were carried out with the specific APRc antibody. As depicted in Figure 7B, a major reactive species with an apparent molecular mass of 21 kDa was detected in *R. rickettsii* whole cell lysate and in the insoluble fraction of the *R. conorii* extract. These results clearly confirmed that *rc1339* gene and its *R. rickettsii* homologue are indeed translated in both rickettsial species. Interestingly, and as previously observed in *E. coli*, a molecular weight of around 21 kDa was also detected for APRc in rickettsial extracts. Although we cannot exclude abnormal migration of the protease in the gel, the observed lower molecular weight may also be correlated with APRc processing at the N terminus, as anticipated by our results in *E. coli*.

To provide additional insights on the localization of APRc in these rickettsial species, fractionation studies were performed on purified bacteria. Whole cell lysates as well as isolated inner and outer membrane fractions were separated by SDS-PAGE and analyzed by Western blots. For both species tested, our results were consistent with localization of the protease at the outer membrane, as confirmed by the immunodetection of rickettsial OmpB, which was used as an internal marker for the outer membrane in these assays (Figure 7C). We further confirmed the presence of APRc on the surface of intact *R. conorii* by flow cytometry analysis and also showed that the enzyme's catalytic domain is presented to the extracellular milieu (Figure 7D). Together, these results further illustrate that a novel retropepsin-like enzyme is expressed in two pathogenic rickettsial species and that the APRc catalytic domain is oriented towards the extracellular environment when present at the outer membrane of these bacteria.

### Recombinant *R. conorii* APRc is sufficient to mediate cleavage of OmpB

**Figure 8:**
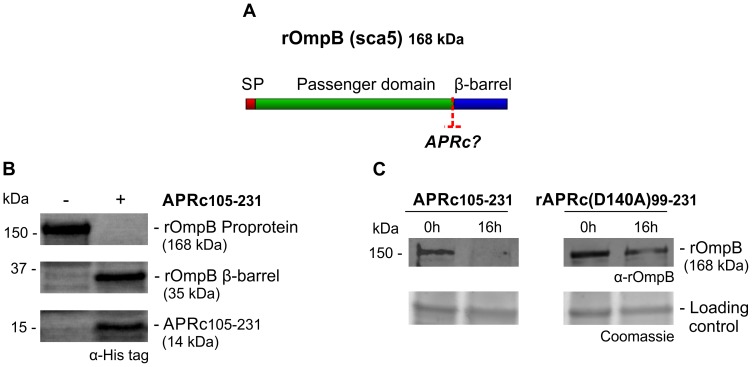
APRc can process rOmpB *in vitro*. (A) rOmpB is proteolytically processed between the passenger and β-barrel domains through a yet unknown mechanism (**?**) and APRc was tested as the candidate enzyme to perform rOmpB preprotein processing *in vitro*. (B) Total membrane fractions of *E. coli* enriched in rOmpB were incubated with activated APRc soluble domain and the reaction products analyzed by Western blot with an anti-Histidine antibody. The integrity of rOmpB proprotein was confirmed in the absence of APRc whereas in the presence of the protease a product with approximately 35 kDa was observed, correlated with the disappearance of the full-length unprocessed form. (C) The integrity of recombinant rOmpB was further evaluated upon incubation with both activated APRc and the active site mutant form (D140A) for 16 h. The reaction products were then subjected to immunoblot analysis with anti-rOmpB MAb, confirming the disappearance of rOmpB in the presence of the active form of the enzyme. Molecular weight markers in kilodaltons (kDa) are shown on the left. Protein loading controls: Coomassie blue staining.

**Figure 9:**
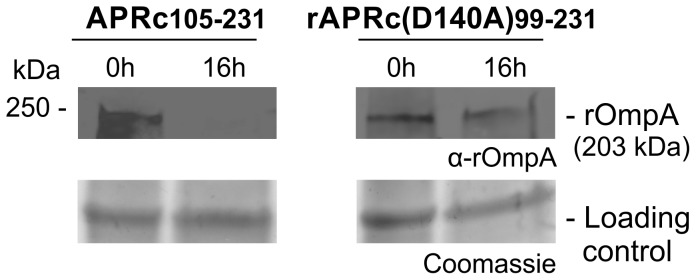
APRc can process rOmpA *in vitro*. Total membrane fractions of *E. coli* enriched in rOmpA were incubated with both activated APRc and the active site mutant form (D140A) for 16 h. The reaction products were then subjected to immunoblot analysis with anti-rOmpA Ab, confirming the disappearance of rOmpA in the presence of the active form of the enzyme. Molecular weight markers in kilodaltons (kDa) are shown on the left. Protein loading controls: Coomassie blue staining.

The evidence that a proportion of APRc is associated with the outer membrane led us to hypothesize that rickettsial surface proteins might be potential substrates for this newly characterized enzyme. As has been shown for other autotransporter proteins, rickettsial OmpB, OmpA, Sca1, and Sca2 are involved in mediating important interactions with mammalian cells and undergo processing events at the outer membrane [43]–[49]. As an example, *R. conorii* OmpB (rOmpB) is expressed as a preprotein of 168 kDa and is subsequently cleaved to release the passenger domain (120 kDa) from the β-barrel translocation domain (32 kDa) [46]. Interestingly, *R. conorii* and *R. japonica* OmpB do not undergo proteolytic cleavage when expressed at the outer membrane of *E. coli*, suggesting that the processing event is not autocatalytic [43]. However, the identity of the enzyme responsible for Sca protein maturation still remains elusive. Therefore, and based on the observed APRc outer membrane localization, we sought to determine whether APRc might participate in the processing of rOmpB (Figure 8A). In order to do this, we performed transactivation assays using *E. coli* outer membrane fractions enriched in recombinant rOmpB (C-terminally His-tagged) and purified active APRc (soluble catalytic domain). Reaction products were then separated by SDS-PAGE and analyzed by Western blot. As shown in Figure 8B, the detection of an anti-His immune reactive product with ∼35 kDa in the presence of APRc was correlated with the disappearance of rOmpB preprotein, suggesting that this enzyme may be indeed capable of promoting cleavage of recombinant rOmpB. Moreover, the generated reactive protein product has approximately the same molecular weight as that expected for rOmpB β-barrel (32 kDa), further suggesting that this proteolytic cleavage may likely be occurring somewhere between the passenger and the β-barrel domains, in agreement with what has been described for native rOmpB [46]. To further validate these results, parallel assays were performed in the presence of APRc active site mutant and the integrity of rOmpB proprotein evaluated by immunoblotting with a specific antibody to this outer membrane protein. As expected, the disappearance of rOmpB proprotein was observed in the presence of active APRc but not when the cell extract was incubated with the active site mutant protein (APRc(D140A)_99–231_). Interestingly, we observed a similar phenomenon using as a substrate another conserved rickettsial antigen, Sca0/OmpA, demonstrating that a protein other than OmpB can be processed by APRc *in vitro* (Figure 9). Altogether, these results suggest that APRc is sufficient to mediate rOmpB maturation and rOmpA maturation *in vitro*, thereby raising an exciting hypothesis regarding possible functional significance of APRc as being able to process these and possibly other autotransporter proteins in the context of intact *R. conorii* cells.

## Discussion

The intrinsic difficulty in working with obligate intracellular parasites such as rickettsiae greatly hampers the correlation of rickettsial gene products with their function. Therefore, valuable information on the nature of conserved genes as well as on the identification of new bacterial factors that may play a role in rickettsiae pathogenesis is mostly being provided by comparative genomics. Using this approach, we identified a gene encoding a putative membrane embedded aspartic protease with a retroviral-type signature, highly conserved in 55 *Rickettsia* genomes. Using the *R. conorii* gene homologue RC1339 as our working model we demonstrate that the gene product (APRc) displays a high degree of identity among *Rickettsia* spp., although no significant homology is observed when compared to other aspartic proteases, except for the conservation of the motif around the catalytic aspartate as well as the hydrophobic-hydrophobic-glycine motif required for the formation of the psi loop. These features resemble the retroviral APs comprising family A2, which are characterized by being active only as symmetric dimers with a single active site, where each monomer contributes one aspartate [25], [27]. Despite the observed low overall sequence similarity with retropepsins, our results on the enzymatic characterization of the soluble catalytic domain of RC1339/APRc further revealed that this novel rickettsial enzyme indeed shares several properties with this family of APs. The common properties include autolytic activity impaired by mutation of the catalytic aspartate, accumulation in the dimeric form, optimal activity at pH 6, inhibition by specific HIV-1 PR inhibitors, and specificity preferences resembling those of both AP families. The presence of retroviral-type APs in bacteria has been previously demonstrated (SpoIIGA in *Bacillus subtilis* [33] and PerP in *Caulobacter crescentus* [50]). However, no enzymatic characterization is yet available for these enzymes and their inclusion as retropepsin-type protease members has not been universally accepted [29]. Therefore, the results described here provide experimental substantiation that RC1339/APRc is a novel retropepsin-like enzyme expressed in bacteria.

Most viral retropepsins are strictly required for the processing of Gag and Gag-Pol polyproteins into mature structural and functional proteins (including themselves) and are, therefore, indispensable for viral maturation [51]. Because of this, retropepsin-type APs are generally characterized by their inherent autolytic function. Interestingly, our results with APRc soluble catalytic domain fused to GST also demonstrated the ability of this protein to undergo a multi-step autocatalytic conversion *in vitro* into APRc_105–231_ mature form, and this autolytic activity was again confirmed when the last intermediate of activation was produced in *E. coli*. As expected for a retropepsin-like enzyme, mutation of the catalytic aspartate impaired this process. The enzymatic activity assays performed during these autoactivation studies (either using oxidized insulin B chain or the fluorogenic peptide mimicking the final cleavage site between the Ser104-Tyr105 residues) clearly indicated that APRc activity appears to be dependent on the presence of the final activation product. These results suggest that the processing at the N terminus must be the determining step for the regulation of enzymatic activity, presumably through a conformational change occurring upon conversion from rAPRc_99–231_ to APRc_105–231_ form. This is in line with what has been already described for recombinant HIV-1 PR, where the increase in catalytic activity upon protease autolytic conversion has been correlated with a conformational rearrangement between the precursor/inactive vs. mature/active forms of the enzyme [51], [52]. However, further studies are required to better understand the maturation of APRc precursor forms *in vitro* and how this is accomplished and controlled *in vivo*. In fact, we have shown that APRc accumulates in the outer membrane in *R. conorii* and *R. rickettsii* and, therefore, we cannot rule out that the presence of the transmembrane domain may play an important role in this maturation process *in vivo*.

Another interesting observation was that APRc autolytic activity, as well as cleavage of the fluorogenic substrate, occurred at a pH optimum of 6.0. This is again in good agreement with the optimal pH of other retropepsin-like [37], [38] enzymes as well as of the pepsin-like renin [53], [54] and, actually, it is consistent with the presence in APRc of an alanine residue downstream from the catalytic motif (Asp-Thr-Gly-Ala), instead of the common threonine residue found in most pepsin-like APs [27]. Together with the observed inhibitory effect of specific HIV-1 PR inhibitors, these results strengthen the striking resemblance between the enzymatic properties of APRc and those of viral retropepsins. Unexpectedly, we observed a drastic inhibitory effect of EDTA on both APRc maturation and hydrolysis of the fluorogenic substrate, suggesting that this protease may depend on a metal ion for folding and/or activity. A similar effect has not been reported for other retropepsins and no homology to a metalloprotease consensus motif was identified in APRc that could justify this inhibition. Therefore, further structural studies will be required to help in understanding this effect.

To provide additional evidence on the nature of APRc as a retropepsin-like AP we determined both the prime and nonprime side specificity using PICS [39]. Although HIV-1 PR is the only AP for which a PICS analysis has been reported [40], there are several studies for many different APs on specificity towards individual substrates (compiled, at least partially, by MEROPS [55]) providing a collection of cleavage patterns for these enzymes. A comparison of the substrate specificity of APs with our PICS results confirmed common preferences between APRc and both retropepsin and pepsin-type APs. The amino acid preference of APRc for P1 position is in good agreement with the canonical specificity of APs for large hydrophobic amino acids, such as phenylalanine, methionine, carboxyamidomethylated cysteine (which results from the modification during generation of peptide libraries), or leucine. Despite the observed lower selectivity, a similar trend for accommodating hydrophobic amino acids is also observed in P1′. As observed in both tryptic and GluC libraries, APRc appears to display broader specificity for P1 and P1′, while a more constrained amino acid preference is observed for P3, P2, and P2′ positions. This observation may account for an important role on substrate recognition and binding to the active pocket site and may ultimately influence hydrolytic efficiency. Strikingly, a high degree of similarity is found with more specialized pepsin-like proteases such as BACE for P3 (with a preference for valine and isoleucine) and P2′ (alanine and valine) positions, as well as with cathepsin D (also for P2′). Interestingly, APRc also displays unique amino acid preferences such as proline at P2 (although the preference observed for valine and threonine in this position has also been described for feline immunodeficiency virus retropepsin [55], [56]), and leucine and isoleucine in P4′ position. When compared with the two major types of cleavage sites proposed for HIV-1 PR and other retropepsins, APRc specificity profile suggests a preference for type 2-like substrates with hydrophobic amino acids in P1 and P1′, whereas type 1-like substrates with the typical combination of tyrosine(phenylalanine)-proline at P1-P1′ appear disfavored [51], [57]. Moreover, our results suggest a cooperative effect between P2 and P1 positions by revealing that a P2 proline co-occurs more frequently with P1 methionine or asparagine residues and that proline is not favored at this position when P1 is occupied by phenylalanine. Curiously, APRc autolytic cleavage sites do not perfectly match the observed specificity preferences of the activated form used in PICS, suggesting either a different conformational arrangement of the protease or a dependence on the sequence context and/or conformation of the substrate. This is not totally unexpected, as for HIV-1 PR it has also been reported that specificity towards nonviral protein substrates significantly differed from viral polyprotein cleavage sites [51].

Aspartic proteases were assumed for a long time to be restricted to viruses and eukaryotes. However, more recently proteins bearing the characteristic hallmark features of the pepsin family have been identified in seven bacterial genomes [29] and the detailed biochemical characterization of the pepsin-like homologue from the *Shewanella amazonensis*, shewasin A [58], has clearly demonstrated that this bacterial AP is strongly reminiscent of its eukaryotic counterparts. These observations have raised a discussion on the evolutionary relationships between bacterial and eukaryotic pepsin-like APs, by suggesting that bi-lobal pepsin-like proteases may have evolved from primordial homodimeric aspartic proteases before divergence between eukaryotes and prokaryotes (through the proposed gene duplication and fusion event [59]). Our current results on RC1339/APRc further support this hypothesis by providing the first experimental evidence that a gene for a single-lobed AP is indeed present in prokaryotes, coding for an active enzyme with properties resembling those of retropepsins. Moreover, these results offer additional clues on the relationships between retropepsin-like and pepsin-like APs. The presence of single-lobed AP genes in prokaryotes suggests that enzymes such as APRc may actually represent the most ancestral forms of these proteases, whereas retroviral retropepsins may instead correspond to a derived state.

Besides demonstrating that RC1339 encodes an active enzyme, we have also shown that this rickettsial protease is expressed in both *R. conorii* and *R. rickettsii*. Unlike the large majority of α-helical type of integral membrane proteins, sub-cellular localization studies revealed an outer membrane accumulation for APRc which was also confirmed by expression of the full-length protease in *E. coli* and in the context of intact *R. conorii*. So far, only three transmembrane proteins with α-helical architecture have been reported to be embedded in the outer membrane of gram-negative bacteria [60]–[62]. Therefore, our results provide additional evidence that the bacterial surface is not restricted to proteins with β-barrel structures known to play essential roles in energetics, metabolism, signal transduction, and transport [63], [64], further suggesting that the repertoire of proteins with α-helices localized to the OM may be higher than anticipated. Nevertheless, transport and insertion of APRc into the OM definitely requires further studies in order to clarify whether the detected 21 kDa band is an intermediate processed form or the result of different gel mobility.

In line with our evidence for the native expression of APRc in *R. conorii* and *R. rickettsii*, a multiomics study performed in *Rickettsia prowazekii* to identify potential virulence factors has also confirmed transcription of *RC1339* gene homologue (*RP867*) [17]. Importantly, these studies also showed differential regulation of *RP867* expression in different strains of *R. prowazekii* with a fold change of 1.77 between the virulent strain Rp22 and the avirulent strain Erus. This evidence for an up-regulation of APRc's gene expression in *R. prowazekii* Rp22, combined with our results confirming protease expression and accumulation into the OM in *R. conorii* and *R. rickettsii*, strongly support a relevant role of this highly conserved protease in rickettsial life cycle. Serine-, cysteine-, and metalloproteases are widely spread in many pathogenic bacteria, where they play critical functions related to pathogenesis and virulence [19], [20]. However, much less is known about the role of aspartic proteases since the presence of this class of enzymes in pathogenic bacteria has not been previously reported. Taking under consideration the unique biochemical and enzymatic features of APRc presented in this work: i) the apparent non-stringent sequence requirement; ii) outer membrane localization and extracellular orientation of recombinant APRc catalytic domain and iii) autolytic activity suggesting that the soluble biological unit may be released from the surface of rickettsial cells by an ectodomain shedding-like process, we anticipate a potential multi-functional role for this rickettsial protease. One of the proposed functions concerns APRc contribution for the degradation of host tissues for supplying bacteria with nutrients, similar to that described for other extracellular proteases secreted by many pathogens [19]. Second, this protease may also support the spread of the infection and dissemination of bacteria into deeper tissue through the shedding of cell surface adhesion molecules or the inactivation of the components of the host immune system such as proteins from the complement system [21], [65], [66]. And third, APRc may participate in the degradation and/or maturation of other rickettsial proteins, in particular those located at the OM, such as Sca proteins [45], [67], exemplified by Sca5/rOmpB and Sca0/OmpA. In contrast to other autotransporter proteins from gram-negative bacteria with auto-proteolytic activity such as SPATEs (Serine Protease Autotransporters) [68], rOmpB processing is thought to implicate a protease as previous expression studies in *E. coli* have failed to demonstrate autocatalytic activity [43], [47]. In this work, we have started addressing this last hypothesis and we showed that APRc is indeed sufficient to catalyze the processing of Sca0/OmpA and Sca5/rOmpB *in vitro* and that, for the latter, the generated product is consistent with the cleavage between the passenger and the β-peptide regions. The N-terminal sequence of the β-peptide has been experimentally determined for *R. typhi* and *R. prowazekii* [46] rOmpB and the region spanning the cleavage site (/) corresponds to the sequence Ala-Ala-Val-Ala-Ala/Gly-Asp-Glu-Ala-Val. Although we cannot exclude that in *R. conorii* the cleavage of rOmpB may occur slightly upstream from this region, if considering a similar cleavage site the amino acids present in P4, P3, P1, P1′ and P4′ are in good agreement with the observed specificity preferences for APRc, while the differences observed for the remaining positions may reflect again the importance of sequence context/substrate conformation for APRc cleavage. Nevertheless, additional experiments are required to determine the cleavage site and its relevance in the context of intact rickettsiae as well as APRc role in the degradation of other rickettsial and/or host proteins.

In summary, the findings described herein show that this newly characterized aspartic protease from *Rickettsia* is an active enzyme with features highly reminiscent of retropepsin-type proteases and we anticipate its participation in a relevant proteolytic pathway in rickettsial life-cycle, likely as a modulator of activity of other rickettsial membrane-localized proteins. Determination of APRc three-dimensional structure and dissection of its contribution to rickettsial pathogenesis will be critical to start unveiling the significance of this novel protease as a potential target for therapeutic intervention.

With this work we expect to contribute to start changing the currently accepted evolutionary paradigm of aspartic proteases, by positioning what we denominate as “prokaryopepsins” as the new archetypes of modern APs.

## Materials and Methods

### Materials

Oligonucleotide primers were purchased from Integrated DNA Technologies, Leuven, Belgium. Synthetic genes encoding the full-length RC1339 and the predicted soluble catalytic domain, the fluorogenic peptide PepRick14 (MCA-Lys-Ala-Leu-Ile-Pro-Ser-Tyr-Lys-Trp-Ser-Lys-DNP) and the rabbit polyclonal antibody raised towards the sequence Cys-Tyr-Thr-Arg-Thr-Tyr-Leu-Thr-Ala-Asn-Gly-Glu-Asn-Lys-Ala (anti-APRc) were produced by GenScript (Piscataway, NJ, USA). N-terminal amino acid sequence analyses were performed in the Analytical Services Unit - Protein Sequencing Service, ITQB (Oeiras, Portugal). Rabbit polyclonal antibody against the purified APRc_99–231_His construct (see below) was generated by standard immunization schemes approved by the LSU School of Veterinary Medicine Institutional Animal Care and Use Committee (IACUC).

### Bioinformatics analysis

Gene and protein sequences for *R. conorii* str. Malish 7 RC1339 were obtained from the genome sequence at NCBI (NC_003103) (AAL03877). Amino acid sequence alignment and the degree of identity between RC1339/APRc homologues from *Rickettsia* (genus) (TaxID 780) were obtained with ClustalW [69], by comparing the sequences deposited in NCBI database with the following accession numbers: NP_360976 (*R. conorii* str. Malish 7), YP_005393543 (*R. parkeri* str. Portsmouth), YP_001495413 (*R. rickettsii* str. Sheila Smith), YP_005364747 (*R. amblyomii* str. GAT-30V), YP_005391701 (*R. montanensis* str. OSU 85–930), NP_221215 (*R. prowazekii* str. Madrid E), YP_067793 (*R. typhi* str. Wilmington), YP_247382 (*R. felis* URRWXCal2) and YP_001495500 (*R. bellii* OSU 85–389). The protein family, domain, and functional sites were searched using the InterProScan program [70]. Topology structure was predicted with HMMTOP2 algorithm [30]. A structure-based alignment of RC1339/APRc soluble catalytic domain with HIV-1 (PDB 3hvp), EIAV (PDB 2fmb), and XMRV (PDB 3nr6) retropepsins and with DdI1 putative protease domain (PDB 2i1a) was performed with PROMALS3D [71].

### DNA constructs

The sequences encoding the full-length and the predicted soluble domain of RC1339/APRc were chemically synthetized with OptimumGene codon optimization technology for *E. coli* codon usage and cloned into pUC57 vector. The gene encoding the full-length APRc (construct coding amino acids 1–231) was then amplified to include restriction sites for NcoI and NotI at 5′- and 3′-ends, respectively, using the forward primer 5′-CCATGGGAATGAACAAAAAACTGATCAAACTG-3′ and the reverse primer 5′-CTCGAGATAATTCAGAATCAGCAGATCTTT-3′; the resulting PCR product was cloned into pGEM-T Easy plasmid (Promega). After digestion with NcoI and NotI, APRc_1–231_ insert was subcloned into pET28a expression vector (Invitrogen) in frame with a C-terminal His-tag (pET-APRc_1–231_His). In order to generate the untagged construct, an insertion mutagenesis was performed to include the TGA stop codon at the end of the full-length sequence using the Quick Change site-directed mutagenesis kit (Stratagene) and the primers 5′-ATTCTGAATTATTGACTCGAGCACCAC-3′ (forward) and 5′-GTGGTGCTCGAGTCAATAATTCAGAAT-3′ (reverse) (pET-APRc_1–231_).

The optimized sequence encoding the predicted soluble catalytic domain of APRc (construct coding amino acids 87–231) flanked by restriction sites for BamHI (5′)/EcoRI (3′) was inserted in frame to the C terminus of GST in pGEX-4T2 expression vector (Amersham) using the same pair of restriction enzymes (pGST-APRc_87–231_).

For generating the expression construct bearing the sequence encoding the intermediate activation form APRc_99–231_ (construct coding amino acids 99–231), the sequence was firstly amplified using the construct pETAPRc_1–231_ as the template and the forward primer containing a NdeI restriction site (5′-CATATGTATAAATGGAGTACCGAAGTT-3′) and the same reverse primer used for amplification of APRc_1–231_ (5′-CTCGAGATAATTCAGAATCAGCAGATCTTT-3′), and cloned into pGEM-T Easy (Promega). The insert was then digested with NdeI/NotI and subcloned into pET23a expression vector (Invitrogen) in frame with a C-terminal His-tag (pETAPRc_99–231_His).

The active site mutant of APRc (both in pGEX4T2 and pET23a constructs) was generated by replacing the putative active site aspartic acid residue by alanine (D140A) using the Quick Change site-directed mutagenesis kit (Stratagene) and the primers 5′-AAAATCAAATTCATGGTGAATACCGGCGCCTCTGATATTGCA-3′ (forward) and 5′-TGCAATATCAGAGGCGCCGGTATTCACCATGAATTTGATTTT-3′ (reverse) (mutation underlined). All positive clones were selected by restriction analysis and confirmed by DNA sequencing.

The construct for expression of rOmpB in *E. coli* was generated as described elsewhere [43].

### Expression and purification of the soluble forms of APRc

rGST-APRc_87–231_ and the corresponding active site mutant protein were expressed by standard procedures. Briefly, *E. coli* BL21 Star (DE3) cells transformed with each plasmid construct, pGST-APRc_87–231_ and pGST-APRc_87–231_(D140A), were grown at 37°C until an OD_600 nm_ of 0.7. Protein expression was then induced with 0.1 mM IPTG for 3 hours, after which cells were harvested by centrifugation at 9000 *g* for 20 minutes at 4°C, and resuspended in PBS buffer. Lysozyme (100 µg/ml) was added and the harvested cells were frozen at −20°C. After freezing and thawing, bacterial cell lysates were incubated with DNase (1 µg/ml) and MgCl_2_ (5 mM) for 1 hour at 4°C. The total cell lysate was then centrifuged at 27216 *g* for 20 minutes at 4°C and the resulting supernatant filtered (0.2 µm) before loading onto a GSTrap HP 5 ml column (GE Healthcare Life Sciences) previously equilibrated in PBS buffer. After extensive washing, the protein of interest was eluted in 50 mM Tris-HCl pH 8 with 10 mM glutathione and immediately loaded onto a Superdex 200 HiLoad 26/60 (GE Healthcare Life Sciences) equilibrated in PBS buffer for further purification and glutathione removal.

Expression of *E. coli* BL21 Star (DE3) cells transformed with pETAPRc_99–231_His plasmid as well as isolation of total soluble protein were performed under the same conditions as described for rGST-APRc_87–231_, except that in this case the cell pellet was resupended in 20 mM phosphate buffer pH 7.5, 500 mM NaCl and 10 mM imidazole. The resultant supernatant was then loaded onto a Histrap HP 5 ml column (GE Healthcare Life Sciences) pre-equilibrated in the same buffer. Protein elution was performed by a three-step gradient of imidazole (50 mM, 100 mM and 500 mM) and fractions containing the protein of interest (100 mM imidazole gradient step) were pooled and buffer exchanged into 20 mM phosphate buffer pH 7.5 by an overnight dialysis step. Dialyzed protein was further purified by cation-exchange chromatography with a MonoS column (GE Healthcare Life Sciences) equilibrated in the same buffer and elution was carried out by a linear gradient of NaCl (0–1 M).

### Auto-processing studies

Time-course studies of APRc activation were undertaken with two recombinant forms of the soluble catalytic domain of APRc (rGST-APRc_87–231_ and rAPRc_99–231_). Purified samples of rAPRc were first diluted to 0.1 mg/mL with PBS and then diluted 1∶1 with 0.1 M sodium acetate buffer pH 6. Diluted samples were incubated up to 48 h at 37°C and aliquots were taken every 12 h for SDS-PAGE analysis and proteolytic activity assays. To evaluate the effect of inhibitors on rAPRc auto-activation processing, a time-course analysis was carried out in the presence of 20 µM pepstatin, 1 mM indinavir or 5 mM EDTA and protein samples were analyzed by SDS-PAGE.

### Dimerization assays

Crosslinking reactions with disuccinimidyl suberate (DSS) (Pierce) were performed in 20 mM phosphate buffer pH 7.5 containing 150 mM NaCl. A solution of 0.2 mg/ml of purified rAPRc (rAPRc_99–231_ and activated product rAPRc_105–231_) was treated with a 50-fold molar excess of DSS in a total volume of 50 µl and allowed to react for 30 min at room temperature. For glutaraldehyde treatment, a solution of 0.5 mg/ml of purified rAPRc_99–231_ was treated with 5 µl of 1.15% freshly prepared solution of glutaraldehyde for 4 minutes at 37°C, in a total volume of 50 µl, under similar buffer conditions. To terminate the reactions, 5 µl of the quenching buffer 1 M Tris-HCl pH 8.0 were added. Crosslinked proteins were separated by SDS-PAGE and analyzed by Western blot with anti-APRc antibody.

### Analytical size-exclusion chromatography

Precursor rAPRc_99–231_ and activated rAPRc_110–231_ forms were analyzed under nondenaturing conditions by analytical size-exclusion chromatography on a Superdex 200 5/150 GL (GE Healthcare Life Sciences) column connected to a Prominence HPLC system (Shimadzu Corporation, Tokyo, Japan). The column was equilibrated in 20 mM phosphate buffer pH 7.5 containing 150 mM NaCl, and calibrated with Gel Filtration LMW and HMW calibration kits (GE Healthcare Life Sciences), according to the manufacturer's instructions. The molecular mass markers used for calibration were conalbumin (75 kDa), ovalbumin (43 kDa), carbonic anhydrase (29 kDa), and ribonuclease A (13.7 kDa).

### Enzyme activity assays

The effect of pH on activity and inhibitory profile of purified recombinant rAPRc (rAPRc_105-231_) was determined by fluorescence assays in 96-well plates in in a Gemini EM Fluorescence Microplate Reader, using the fluorogenic substrate PepRick14 (MCA-Lys-Ala-Leu-Ile-Pro-Ser-Tyr-Lys-Trp-Ser-Lys-DNP) (final concentration of 2.5 µM). For determination of the pH profile, rAPRc_105-231_ was assayed for activity at 37°C in buffers ranging between pH 4 and 9 (50 mM sodium acetate pH 4.0, 5.0, 5.5 and 6.0; 50 mM Tris-HCl pH 7.0, 8.0 and 9.0) containing 100 mM NaCl. To test the effect of classical inhibitors, the protease was pre-incubated in the presence of each inhibitor, 20 µM pepstatin, 5 mM EDTA, 1 mM ZnCl_2_, 1 mM Pefabloc, or 10 µM E-64, for 10 minutes at room temperature in 50 mM sodium acetate pH 6.0 containing 100 mM NaCl before determination of proteolytic activity. The effect of the HIV inhibitors on rAPRc proteolytic activity was also evaluated. The following reagents were obtained through the NIH AIDS Research and Reference Reagent Program, Division of AIDS, NIAID, NIH: indinavir sulfate, nelfinavir, ritonavir, saquinavir, amprenavir, atazanavir sulfate, sarunavir, and lopinavir. Each inhibitor was again incubated with rAPRc for 10 minutes at room temperature in 50 mM sodium acetate pH 6.0 containing 100 mM NaCl and 5% DMSO, except for indinavir and darunavir that were assayed without DMSO. Indinavir, nelfinavir, ritonavir, saquinavir, amprenavir, atazanavir and lopinavir were tested in the range of 0.25 mM–1 mM and the inhibitor darunavir in the range of 2.5 µM–10 µM (final concentration). The rate of substrate hydrolysis was monitored for 3 hours by the increase in fluorescence intensity with excitation/emission wavelengths of 328/393 nm and the relative activity normalized by setting rAPRc activity as 100%.

Evaluation of proteolytic activity during auto-processing time-course analysis was performed towards oxidized insulin β chain. Substrate (1 mg/ml) was incubated with purified recombinant APRc enzyme: substrate mass ratio of 1∶15) in 0.1 mM sodium acetate buffer pH 6.0. After an overnight incubation at 37°C the reaction mixture was centrifuged at 20000 *g* during 6 min and the digestion fragments were separated by RP-HPLC on a C18 column (KROMASIL 100 C18 250, 4.6 mm), using a Prominence system (Shimadzu Corporation, Tokyo, Japan). Elution was carried out with a linear gradient (0–80%) of acetonitrile in 0.1% v/v trifluoroacetic acid for 30 min at a flow rate of 1 ml/min. Absorbance was monitored at 220 nm.

### PICS

rAPRc (APRc_105-231_) specificity profiling was determined according to the Proteomic Identification of Protease Cleavage Sites (PICS) methodology as described elsewhere [40], with minor changes. Tryptic and GluC peptide libraries were generated from THP1 cells. rAPRc-to-library ratios were 1∶50 (wt/wt) and incubation was performed at 37°C for 16 h in 50 mM sodium acetate buffer pH 6.0, 150 mM NaCl. The separation of carboxy-peptide cleavage products was carried out on a column packed with PepMap C18 resin (Dionex) connected to a LC-MS/MS QSTAR Pulsar (AB SCIEX) operated by the UBC Michael Smith Laboratory/Laboratory for Molecular Biophysics Proteomics Core Facility, and on HALO C18 column (Eksigent) connected to a LC-MS/MS TripleTOF 5600, AB SCIEX (Center for Neuroscience and Cell Biology Proteomics Unit). Peptide sequences were identified with both Mascot [72] and X!Tandem [73], in conjunction with PeptideProphet [74] at a confidence level >95%. Mass tolerance was 10 ppm for parent ions and 0.6 Da for fragment ions. Search parameters were set to identify static modifications as carboxyamidomethylation of cysteine residues (+57.02 Da), dimethylation of lysines (+28.03 Da) and thioacylation of peptide amino termini (+88.00 Da). Semi-style cleavage searches were applied with no constraints for the orientation of the specific terminus. The Web-based PICS service [40] was used to derive nonprime sequences. Sequence logos were generated with IceLogo with a *p*-value of 5% [75].

### RT-PCR analysis

For cDNA synthesis, total RNA was isolated from an aliquot of frozen *R. conorii* Malish 7 and *R. rickettsii* “Sheila Smith”-infected Vero cells using the SurePrep TrueTotal RNA Purification Kit (Fisher Scientific), according to the manufacturer's instructions. After extraction, RNA samples were treated with DNase I, RNase-free set (Thermo Scientific) for 30 minutes at 37°C. The reaction was inactivated by adding 50 mM EDTA and heating the mixture at 65°C for 10 minutes. Next, approximately 1 ug of total RNA was used as the template for reverse transcription using the iScript cDNA Synthesis Kit (BioRad), according to the manufacturer's instructions. For all extracted samples, negative RT-PCR controls were processed in the absence of reverse transcriptase. APRc gene expression was assessed by PCR reaction with the specific primers RC1339_RT-Fwd (5′-AAAGCCGCCCCTATAACCTT-3′) and RC1339_RT-Rev (5′-TCCTGAAACCTTTGAAACGCTC-3′) which were designed for the amplification of a segment with 136 bp. The PCRs were performed in a 50 µl volume, with 1 µl of cDNA as the DNA template, 0.1 µM of each primer, 1× PCR buffer (100 mM Tris-HCl (pH 9.0), 15 mM MgCl2, 500 mM KCl), 200 µM of dNTP mix, and 1 U of *Taq* DNA polymerase (GE Healthcare). The PCR mixtures were incubated at 95°C for 3 min, followed by 35 cycles of 95°C (30 sec), 55°C (30 sec), and 72°C (30 sec). The gene *hrtA* (17 kDa surface antigen) was used as the positive control using the primers Rc_htrA_RT-Fwd (5′-GGACAGCTTGTTGGAGTAGG-3′) and Rc_htrA_RT-Rev (5′-TCCGGATTACGCCATTCTAC-3′). An aliquot of 20 µl of each PCR product was electrophoresed on a 1.7% agarose gel and stained with ethidium bromide. The size of the PCR product was determined by comparison with GeneRuler 1 kb Plus DNA Ladder (Thermo Scientific).

### *R. conorii* and *E. coli* fractionation

Cell fractionation studies with *Rickettsia* spp. were performed as previously described [76]. Briefly, approximately 5×10^6^ plaque forming units (pfu) of purified *R. conorii* Malish 7 or *R. rickettsii* “Sheila Smith” was fixed in 4% paraformaldehyde (PFA) in PBS, washed in PBS and then removed from BSL3 containment after verification that viable rickettsiae were no longer present according to standard operating procedures (SOPs). For whole-cell lysates, cells were resuspended in SDS-PAGE loading buffer and boiled. Total outer membrane proteins were extracted essentially as described in [77]. The sample was resuspended in 1.5 ml of 20 mM Tris pH 8.0 containing 1× protease inhibitor cocktail and then subjected to three rounds of French press treatment for cell lysis. The resulting lysate was centrifuged at 10,000 g for 3 minutes to remove unbroken cells and then incubated in 0.5% sarkosyl at room temperature for 5 minutes. The sarkosyl-treated lysate was centrifuged at >16,000 *g* for 30 minutes at 40°C. The sarkosyl soluble protein fraction was removed and the remaining insoluble pellet representing the outer-membrane protein fraction was washed in 20 mM Tris pH 8.0 and then boiled in 0.5 ml of 20 mM Tris pH 8.0 containing 0.5 ml of 2× SDS sample buffer. Protein samples were aliquoted and frozen at −20°C until use.

For isolation of total and outer membrane fractions of *E. coli* BL21 Star (DE3) cells expressing full-length rAPRc_1–231_ the protocol used was essentially as described in [77]. BL21 (DE3) cells were transformed with pETAPRc_1–231_ construct and grown at 37°C until an OD_600 nm_ of 0.6–0.7. Expression of rAPRc_1–231_ was induced with 0.1 mM IPTG for 3 hours, after which cells were pelleted by centrifugation at 9000 *g* for 20 minutes at 4°C and resuspended in PBS buffer. Cells were then mechanically disrupted on a FrenchPress following the manufacturer's instructions (3×, 1500 psi), and cleared by centrifugation at 20000 *g* for 20 minutes. Total membrane were directly pelleted by ultracentrifugation at 144028 *g* for 1 hour at 4°C and resuspended in PBS buffer. For enrichment and purification of OM proteins, inner membrane proteins were extracted by incubating the supernatant of lysate clearance with sarkosyl (final concentration of 0.5%) at room temperature for 5 minutes. Outer membranes were pelleted by ultracentrifugation at 144028 *g* for 1 hour at 4°C and resuspended in PBS buffer. Total membrane, soluble/sarkosyl-solubilized and outer membrane proteins were resolved by SDS-PAGE and analyzed by immunoblotting using antibodies against APRc, Lep and OmpA, the last two used as internal markers for inner and outer membranes of *E. coli*, respectively.

### Flow cytometry

*E. coli* BL21 (DE3) were transformed with pETAPRc_1–231_ construct and grown at 37°C until an OD_600 nm_ of 0.6–0.7. Protein expression was induced with 0.1 mM IPTG for 3 hours. Cells were then fixed for 20 minutes in 4% PFA and subsequently washed in cold PBS. Fixed cells were incubated with anti-APRc rabbit polyclonal (2 µg/ml) and anti-RNAPα mouse monoclonal antibodies (mAb 4RA2, 50 ng/ml), and then labeled with both goat anti-rabbit IgG Alexa Fluor 488 (Life Technologies) and goat anti-mouse IgG R-PE-Cy5.5 conjugated secondary antibodies (SouthernBiotech) at the specified concentration (4 µg/ml). Bacteria were analyzed by flow cytometry using a BD FACSCalibur (BD Biosciences) instrument and FlowJo software. For analysis of non-permeable *E. coli* cells, positive anti-RNAPα staining cells were gated out, and intact cells analyzed for surface expression of APRc_1–231_ with anti-APRc antibody. Detection of APRc and OmpB on the surface of PFA-fixed, intact *R. conorii* cells was determined using rabbit polyclonal anti-APRc_99–231_ antibody, anti-OmpB mAb 5C7.27 [44] and the appropriate AlexaFluor 488 conjugated secondary antibody as described above for the *E. coli* samples.

### *In vitro* cleavage assays

The expression of rOmpB and rOmpA independently in *E. coli* BL21 (DE3) cells was performed as previously described [43], [78]. To assess for *in vitro* proteolytic cleavage of this outer membrane protein by rAPRc, the total membrane fraction of *E. coli* cells overexpressing rOmpB and rOmpA was isolated as described under the section *R. conorii* and *E. coli* fractionation and then incubated for 16 hours at 37°C in 50 mM sodium acetate pH 4.0, 100 mM NaCl with 25 µg of purified active rAPRc_105–231_. A parallel incubation was performed under similar conditions with the active site mutant rAPRc(D140A)_99–231_ as a negative control. The reaction products were separated by SDS-PAGE and analyzed by Western blot with anti-APRc, anti-rOmpB_35–1334_ and anti-rOmpA [78] rabbit polyclonal antibodies.

### SDS-PAGE and western blotting

SDS-PAGE analysis was performed in a Bio-Rad Mini Protean III electrophoresis apparatus using 4–20% or 12.5% polyacrylamide gels. Samples were treated with loading buffer (0.35 M Tris-HCl, 0.28% SDS buffer pH 6.8, 30% glycerol, 10% SDS, 0.6 M DTT and 0.012% Bromophenol Blue) and boiled for 5 minutes before loading. Gels were stained with Coomassie Brilliant Blue R-250 (Sigma). For Western blot analysis, protein samples were resolved by SDS-PAGE and electrotransferred onto PVDF or nitrocellulose membranes by standard wet (using the buffer 25 mM Tris, 192 mM glycine and 20% methanol) or semi-dry (in buffer 25 mM Tris, 192 mM glycine, 20% methanol and 0.025% SDS) transfer apparatus. Membranes were then blocked for one hour in standard TBS containing 1% (v/v) Tween-20 supplemented with 5% (w/v) skim milk or 2% (w/v) BSA and then incubated with the antibodies, anti-APRc rabbit polyclonal (GenScript, 2 µl/ml), anti-rOmpB_35–1334_ and anti-rOmpA [78] rabbit polyclonal, anti-OmpA (*E. coli*) serum (1∶20000), anti-Lep (*E. coli*) serum (1∶1000). Anti-OmpA and anti-Lep antibodies were kindly provided by Professor Gunnar von Heijne (Stockholm University, Sweden). Membranes were washed in TBS, containing 0.1% (v/v) Tween-20 and incubated with secondary anti-mouse or anti-rabbit alkaline phosphatase-conjugated (GE Healthcare) and IRDye-conjugated (LI-COR Biotechnology) antibodies and revealed using ECF chemiluminescence detection kit (GE Healthcare) in a Molecular Imager FX (Bio-Rad) or by infrared detection using an Odyssey infrared dual-laser scanning unit (LI-COR Biotechnology), respectively.

To confirm the specific band reactivity of anti-APRc antibody, a peptide competition assay was performed. The primary antibody was pre-incubated with 100-fold (mass) excess of immunizing peptide (CYTRTYLTANGENKA) for 20 minutes at room temperature prior immunoblotting analysis and parallel experiments were performed with pre-incubated and non-incubated antibody.

## Supporting Information

Figure S1 **Mutation of the active site aspartic acid impairs activity towards oxidized insulin B chain.** Activity of wt rGST-APRc_87–231_ and rGST-APRc(D140A)_87–231_ towards oxidized insulin B chain was tested upon activation assays *in vitro* in 0.1 M sodium acetate buffer pH 6 at 37°C for 48 h. T48_WT and T48_Mut correspond to the analysis of reaction products by RP-HPLC for the wt and active site mutant, respectively. Ctrl Insulin corresponds to the RP-HPLC profile of oxidized insulin B chain in the absence of protease. The presence of several peaks upon incubation with wt protease is consistent with substrate cleavage.
(TIF)

Figure S2 **Auto-processing activity of the last intermediate of activation rAPRc99–231 and oligomeric status.** (A) The intermediate of activation APRc99–231 fused to C-terminal His-tag was subjected to auto-activation assays in vitro in 0.1 M sodium acetate buffer pH 6 at 37°C for 48 h, in the presence of pepstatin, indinavir and EDTA. Assays were monitored by SDS-PAGE stained with Coomassie blue. In the presence of pepstatin A this conversion was slower and no significant effect was detected under the presence of indinavir. The presence of EDTA completely inhibited protease conversion. (B) The quaternary configuration of rAPRc99–231-His precursor was assessed by incubating the protease with the cross-linker glutaraldehyde. Both glutaraldehyde treated and untreated protein samples were subjected to Western blot analysis with anti-APRc antibody. In the presence of the cross-linking agent, a significant proportion of the protein migrated as a dimer, although the monomeric forms and larger aggregates were also observed. (C) Analysis of precursor rAPRc99–231 and activated rAPRc110–231 forms by analytical size exclusion chromatography. The Superdex 200 5/150 GL was equilibrated in 20 mM phosphate buffer pH 7.5 containing 150 mM NaCl. The black dots refer to elution volumes of molecular mass markers used for calibration. From left to right: conalbumin (75 kDa), ovalbumin (43 kDa), carbonic anhydrase (29 kDa) and ribonuclease A (13.7 kDa).
(TIF)

Table S1 **APRc cleavage sites identified from a tryptic peptide library using Mascot and X!Tandem.** Peptides identified by LC-MS/MS spectrum-to-sequence assignment with Mascot and X!Tandem are listed with PeptideProphet probability score, calculated neutral mass and one exemplary accession number of a matching UniProt protein entry is listed. This data was further processed and rendered non-redundant for generation of cleavage specificity profiles.
(DOCX)

Table S2 **APRc cleavage sites identified from a GluC peptide library using Mascot and X!Tandem.** Peptides identified by LC-MS/MS spectrum-to-sequence assignment with Mascot and X!Tandem are listed with PeptideProphet probability score, calculated neutral mass and one exemplary accession number of a matching UniProt protein entry is listed. This data was further processed and rendered non-redundant for generation of cleavage specificity profiles.
(DOCX)
