# Supplementary figures and images for: RC1339/APRc from Rickettsia conorii Is a Novel Aspartic Protease with Properties of Retropepsin-Like Enzymes
Source: PLoS Pathog. 2014 Aug 21;10(8):e1004324. doi: 10.1371/journal.ppat.1004324 (PMC4140852; doi:10.1371/journal.ppat.1004324)

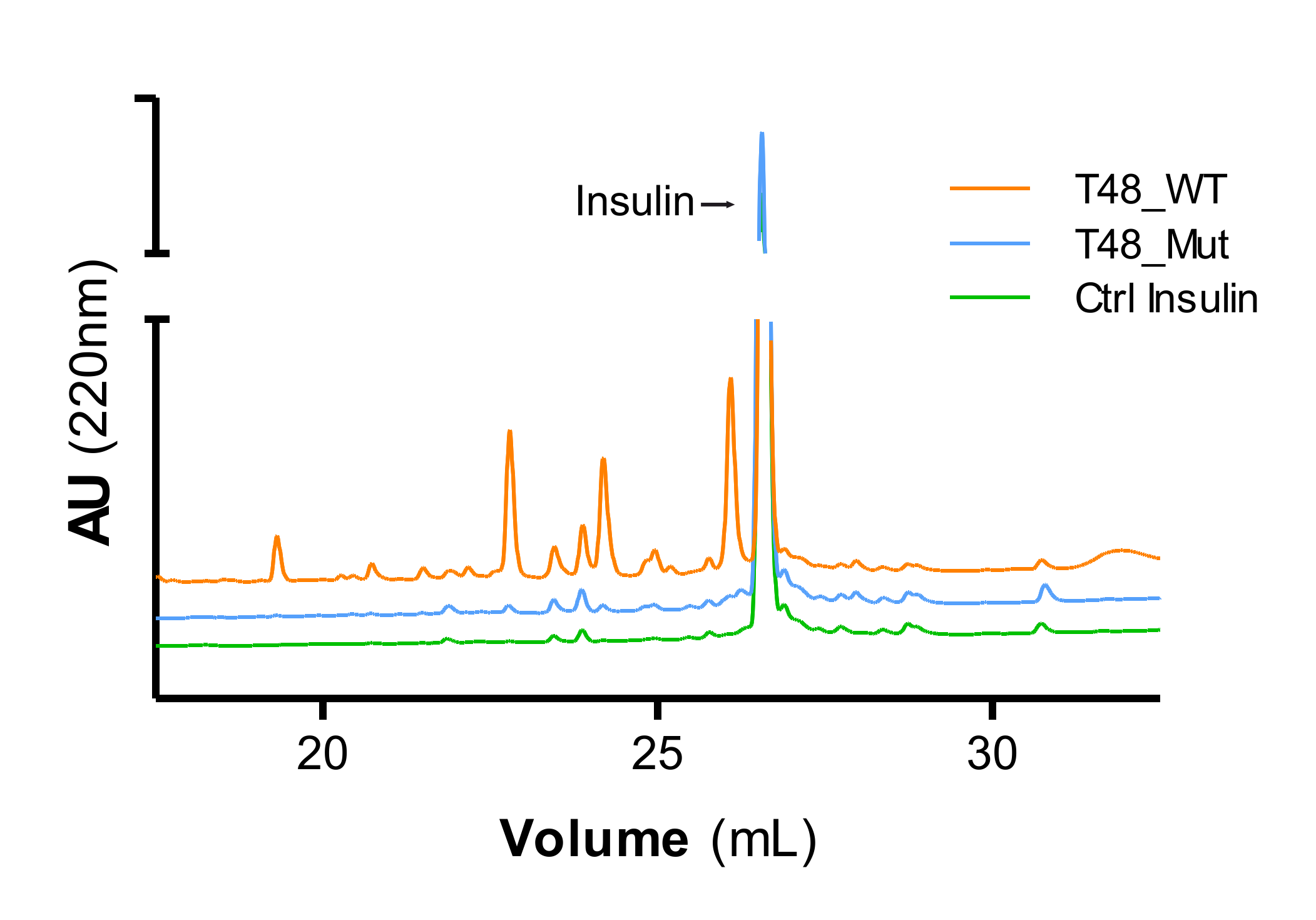

Supplement: Figure S1 — Mutation of the active site aspartic acid impairs activity towards oxidized insulin B chain. Activity of wt rGST-APRc87–231 and rGST-APRc(D140A)87–231 towards oxidized insulin B chain was tested upon activation assays in vitro in 0.1 M sodium acetate buffer pH 6 at 37°C for 48 h. T48_WT and T48_Mut correspond to the analysis of reaction products by RP-HPLC for the wt and active site mutant, respectively. Ctrl Insulin corresponds to the RP-HPLC profile of oxidized insulin B chain in the absence of protease. The presence of several peaks upon incubation with wt protease is consistent with substrate cleavage. (TIF) [file ppat.1004324.s001.tif]

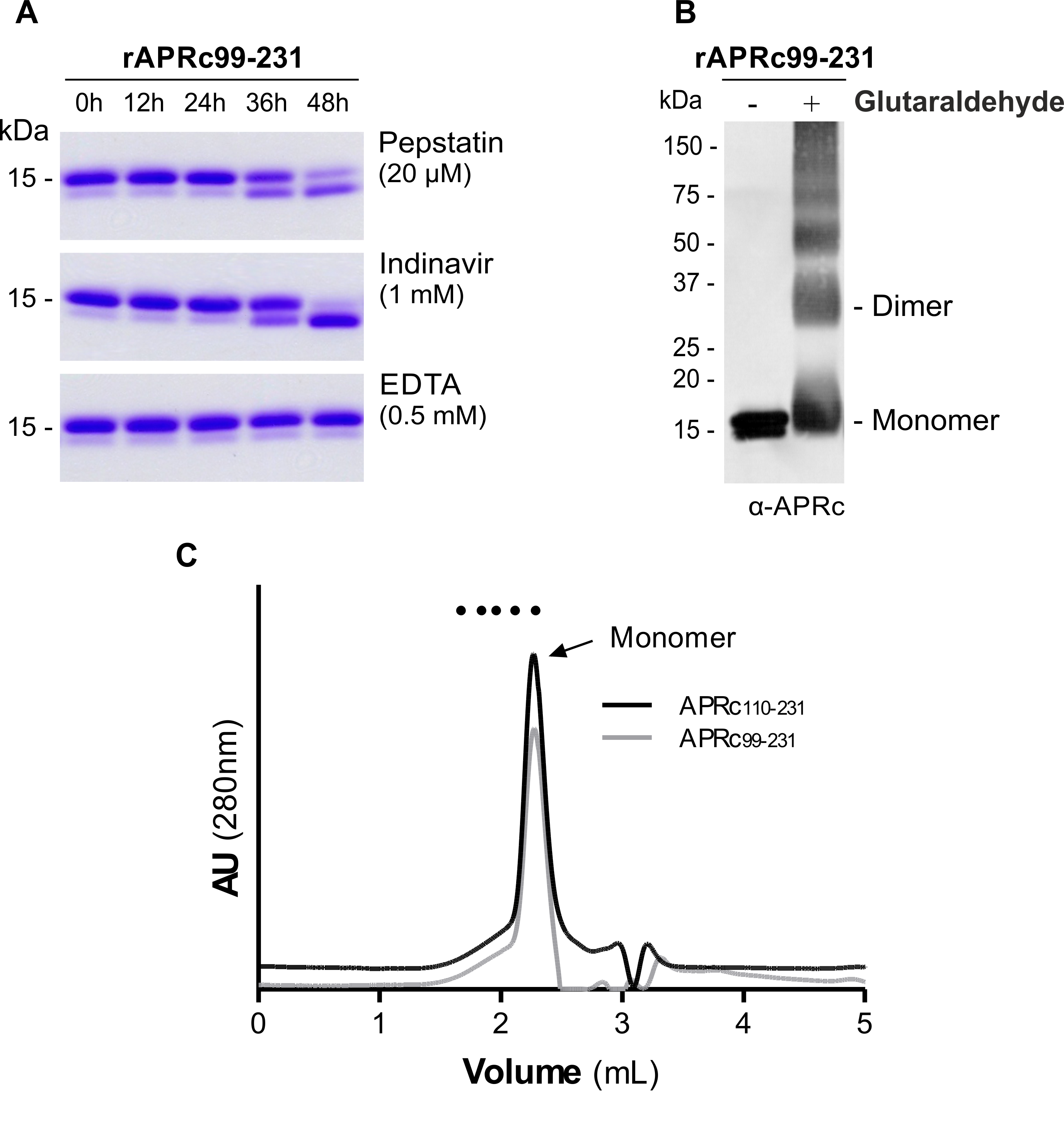

Supplement: Figure S2 — Auto-processing activity of the last intermediate of activation rAPRc99–231 and oligomeric status. (A) The intermediate of activation APRc99–231 fused to C-terminal His-tag was subjected to auto-activation assays in vitro in 0.1 M sodium acetate buffer pH 6 at 37°C for 48 h, in the presence of pepstatin, indinavir and EDTA. Assays were monitored by SDS-PAGE stained with Coomassie blue. In the presence of pepstatin A this conversion was slower and no significant effect was detected under the presence of indinavir. The presence of EDTA completely inhibited protease conversion. (B) The quaternary configuration of rAPRc99–231-His precursor was assessed by incubating the protease with the cross-linker glutaraldehyde. Both glutaraldehyde treated and untreated protein samples were subjected to Western blot analysis with anti-APRc antibody. In the presence of the cross-linking agent, a significant proportion of the protein migrated as a dimer, although the monomeric forms and larger aggregates were also observed. (C) Analysis of precursor rAPRc99–231 and activated rAPRc110–231 forms by analytical size exclusion chromatography. The Superdex 200 5/150 GL was equilibrated in 20 mM phosphate buffer pH 7.5 containing 150 mM NaCl. The black dots refer to elution volumes of molecular mass markers used for calibration. From left to right: conalbumin (75 kDa), ovalbumin (43 kDa), carbonic anhydrase (29 kDa) and ribonuclease A (13.7 kDa). (TIF) [file ppat.1004324.s002.tif]
