# Supplementary material for: RC1339/APRc from Rickettsia conorii Is a Novel Aspartic Protease with Properties of Retropepsin-Like Enzymes
Source: PLoS Pathog. 2014 Aug 21;10(8):e1004324. doi: 10.1371/journal.ppat.1004324 (PMC4140852; doi:10.1371/journal.ppat.1004324)
Supplement: Table S1 — APRc cleavage sites identified from a tryptic peptide library using Mascot and X!Tandem. Peptides identified by LC-MS/MS spectrum-to-sequence assignment with Mascot and X!Tandem are listed with PeptideProphet probability score, calculated neutral mass and one exemplary accession number of a matching UniProt protein entry is listed. This data was further processed and rendered non-redundant for generation of cleavage specificity profiles. (DOCX) [file ppat.1004324.s003.docx]

| **Identified Peptides (prime sequence)** | **PeptideProphet probability** | **Neutral peptide mass (Da)** | **Exemplary protein ID** |
| --- | --- | --- | --- |
| AAAALAAAAVK | 0.9884 | 1042.5923 | Q8TAQ2 |
| AAAAPAKVEAK | 0.9788 | 1169.6556 | Q8NHW5 |
| AAAGAVGSVVGQIAK | 0.9460 | 1413.7728 | Q14914 |
| AAAGYDVEKNNSR | 0.9726 | 1509.6960 | Q02539 |
| AAAIAYGLDK | 0.8889 | 1107.5712 | P11021 |
| AAAIAYGLDK | 0.8592 | 1107.5712 | P11021 |
| AAAIAYGLDKK | 0.9983 | 1263.6975 | P54652 |
| AAAIAYGLDKK | 0.9994 | 1263.6975 | P54652 |
| AAAIAYGLDKK | 0.9996 | 1263.6975 | P54652 |
| AAAIAYGLDKK | 0.8204 | 1263.6897 | Q91883 |
| AAAIAYGLDKRE | 0.9624 | 1392.7149 | P11021 |
| AAALAYGLDKSEDK | 0.9811 | 1594.7991 | P38646 |
| AAASIANIVK | 0.9051 | 1072.6029 | P17987 |
| AAAVDAGMAMAGQSPVLR | 0.8664 | 1802.8555 | P26599 |
| AAEKLQVVGR | 0.9845 | 1185.6618 | O43175 |
| AAGAGATHSPPTDLVWK | 0.9997 | 1793.8849 | P02545 |
| AAGAGATHSPPTDLVWK | 0.9988 | 1793.8849 | P02545 |
| AAGLFLPGSVGITDPCESGNFR | 1.0000 | 2352.0957 | Q12905 |
| AAGLFLPGSVGITDPCESGNFR | 1.0000 | 2352.0957 | Q12905 |
| AAGLSVPNVHGALAPLAIPSAAAAAAAAGR | 0.9955 | 2723.4619 | P26599-2 |
| AAGLSVPNVHGALAPLAIPSAAAAAAAAGR | 1.0000 | 2723.4619 | P26599-2 |
| AAGLSVPNVHGALAPLAIPSAAAAAAAAGR | 0.9954 | 2723.4619 | P26599-2 |
| AAGSTAGSLR | 0.7338 | 977.4678 | Q8TEJ3 |
| AAHVEYSTAAR | 0.9925 | 1262.5792 | P49411 |
| AALILVADNAGGSHASK | 0.9854 | 1709.8849 | Q7Z5L9 |
| AALKNPPINTK | 0.8115 | 1309.7506 | O15511 |
| AAMADTFLEHMCR | 0.9958 | 1639.6693 | P14618 |
| AAMADTFLEHMCR | 0.8411 | 1639.6619 | P00548 |
| AAMTLLSDASHLPK | 0.9239 | 1569.7973 | Q8NBX0 |
| AAPVAAATTAAPAAAAAPAK | 0.9801 | 1777.9474 | P05388 |
| AAQLHLQLQSK | 0.9932 | 1351.7360 | P54920 |
| AASAPVLAVAGLGDSNQFFR | 0.9944 | 2078.0333 | Q9UHL4 |
| AASAPVLAVAGLGDSNQFFR | 1.0000 | 2078.0333 | Q9UHL4 |

| **Identified Peptides (prime sequence)** | **PeptideProphet probability** | **Neutral peptide mass (Da)** | **Exemplary protein ID** |
| --- | --- | --- | --- |
| AASSSSLEK | 0.8859 | 994.4719 | P62736 |
| AASVSSSSLV | 0.9962 | 994.4645 | Q5VS55 |
| AASVSSSSLV | 0.9644 | 994.4645 | Q5VS55 |
| AASVSSSSLV | 0.9884 | 994.4645 | Q5VS55 |
| AASVSSSSLV | 0.9763 | 994.4645 | Q5VS55 |
| AASVSSSSLV | 0.9986 | 994.4645 | Q5VS55 |
| AASVSSSSLV | 0.9718 | 994.4645 | Q5VS55 |
| AASVSSSSLV | 0.9665 | 994.4645 | Q5VS55 |
| AASVSSSSLV | 0.9825 | 994.4645 | Q5VS55 |
| AASVSSSSLV | 0.9942 | 994.4645 | Q5VS55 |
| AASVSSSSLV | 0.9334 | 994.4645 | Q5VS55 |
| AASVSSSSLV | 0.9962 | 994.4645 | Q5VS55 |
| AASVSSSSLV | 0.9813 | 994.4645 | Q5VS55 |
| AASVSSSSLV | 0.9891 | 994.4645 | Q5VS55 |
| AASVSSSSLV | 0.8684 | 994.4645 | Q5VS55 |
| AATLLANHSLR | 0.9973 | 1253.6628 | P13489 |
| AAVDTSSEITTK | 0.9873 | 1337.6388 | P01252 |
| AAVSNLVR | 0.7803 | 916.4878 | P18206 |
| ADALLIIPK | 0.9958 | 1068.6331 | Q92526 |
| ADAPMFVMGVNHEK | 0.9990 | 1660.7416 | P10096 |
| ADAPMFVMGVNHEK | 0.9802 | 1660.7416 | P10096 |
| ADIKAKAQLVK | 0.8479 | 1355.8289 | Q76L83 |
| AEGIHTGQFVYCGK | 0.9896 | 1681.7671 | P62917 |
| AEHQINLIK | 0.9822 | 1180.6352 | P25398 |
| AEILELAGNAAR | 0.9858 | 1314.6680 | P04908 |
| AEVLELAGNASK | 0.9946 | 1316.6724 | Q71UI9 |
| AFADALLIIPK | 0.9928 | 1286.7386 | Q92526 |
| AGGDVCVDR | 0.8103 | 1035.4192 | Q96RT8 |
| AGPNTNGSQFFICTAK | 0.9998 | 1827.8362 | A2BFH1 |
| AGPTALLAHEIGFGSK | 0.9995 | 1683.8732 | Q99497 |
| AGPTALLAHEIGFGSK | 0.9947 | 1683.8732 | Q99497 |
| AGPTALLAHEIGFGSK | 0.9727 | 1683.8732 | Q99497 |
| AGPTALLAHEIGFGSK | 0.9990 | 1683.8732 | Q99497 |
| AGPTALLAHEIGFGSK | 0.8201 | 1683.8732 | Q99497 |
| AGPTALLAHEIGFGSK | 0.9903 | 1683.8657 | Q5E946 |
| AGPTALLAHEIGFGSK | 0.9999 | 1683.8657 | Q5E946 |
| AGPVAEYLK | 0.9992 | 1062.5498 | Q01518 |
| AGPVAEYLK | 0.9794 | 1062.5424 | Q01518 |
| AGPVAEYLK | 0.9444 | 1062.5424 | Q01518 |

| **Identified Peptides (prime sequence)** | **PeptideProphet probability** | **Neutral peptide mass (Da)** | **Exemplary protein ID** |
| --- | --- | --- | --- |
| AGQCGNQIGAK | 0.9914 | 1218.5563 | Q13885 |
| AHAVTQLANR | 0.9212 | 1167.5897 | P78371 |
| AHAVTQLANR | 0.9974 | 1167.5897 | P78371 |
| AHLDATTVLSR | 0.9966 | 1270.6418 | P06576 |
| AHLDATTVLSR | 0.9965 | 1270.6418 | P06576 |
| AHLDATTVLSR | 0.9976 | 1270.6418 | P06576 |
| AHTFNPK | 0.7271 | 929.4507 | P28838 |
| AIAEAWAR | 0.9977 | 974.4722 | Q71U36 |
| AIAEAWAR | 0.9678 | 974.4722 | Q71U36 |
| AIEHADFAGVER | 0.9583 | 1401.6425 | P78371 |
| AIGLSVADLAESIMK | 0.7667 | 1632.8467 | P00338 |
| AILDAVGDDIPVQ | 0.7746 | 1412.6861 | A9HWC3 |
| AILGMDVLCQAK | 0.9994 | 1433.7159 | O00148 |
| AILGMDVLCQAK | 0.9979 | 1433.7159 | O00148 |
| AINPELLQLLPLHPK | 0.9999 | 1811.0457 | Q99661 |
| AIPSAAAAAAAAGR | 0.9997 | 1255.6421 | P26599 |
| AITATQK | 0.9843 | 847.4552 | P04406 |
| AITQVLLLANPQK | 0.9965 | 1523.8823 | Q9UJY5 |
| ALAASALPALVMSK | 0.9495 | 1457.8064 | P36578 |
| ALALFGGEPK | 0.8501 | 1117.5920 | P49736 |
| ALCSLHSIGK | 0.9912 | 1200.6073 | P14174 |
| ALCSLHSIGK | 0.9748 | 1200.6073 | P14174 |
| ALCSLHSIGK | 0.9325 | 1200.6073 | P14174 |
| ALCSLHSIGK | 0.9430 | 1200.6073 | P14174 |
| ALFEDTNLCAIHAK | 0.9998 | 1717.8172 | P02302 |
| ALFPPVEFPAPR | 0.9590 | 1427.7275 | P49327 |
| ALFPPVEFPAPR | 0.7824 | 1427.7275 | P49327 |
| ALGWVAMAPKPGPYVK | 0.9900 | 1843.9807 | Q01518 |
| ALGWVAMAPKPGPYVK | 0.9665 | 1827.9858 | Q01518 |
| ALGWVAMAPKPGPYVK | 0.9813 | 1827.9858 | Q01518 |
| ALGWVAMAPKPGPYVK | 0.9985 | 1827.9858 | Q01518 |
| ALGWVAMAPKPGPYVK | 0.9919 | 1827.9858 | Q01518 |
| ALLAHEIGFGSK | 0.8130 | 1357.7142 | Q99497 |
| ALLPQTLLDQK | 0.9875 | 1354.7608 | P50225 |
| ALLSAPNPDDPLANDVAEQWK | 0.9995 | 2379.1495 | P61088 |
| ALNELLQHVK | 0.9559 | 1279.7036 | Q9Y490 |
| ALPFFGFSEPLAAPR | 0.7521 | 1706.8569 | P22314 |
| ALPHAILR | 0.9866 | 977.5558 | Q562R1 |
| ALPHAILR | 0.8960 | 977.5558 | Q562R1 |

| **Identified Peptides (prime sequence)** | **PeptideProphet probability** | **Neutral peptide mass (Da)** | **Exemplary protein ID** |
| --- | --- | --- | --- |
| ALPHAILRLD | 0.9410 | 1205.6669 | Q562R1 |
| ALQDMLLLK | 0.9015 | 1175.6372 | Q6P2M8-5 |
| ALSENSGMNPIQTMTEVR | 0.9337 | 2064.9356 | P48643 |
| ALTGGIGFIHHNCTPEFQANEVR | 0.9914 | 2655.2401 | P12268 |
| ALVIDNGSGMCK | 0.9327 | 1379.6247 | P53505 |
| ALVIDNGSGMCK | 0.7986 | 1379.6251 | P53505 |
| ALVKPEVWTLK | 0.9346 | 1426.8336 | Q9UL46 |
| ALVLLIAQEK | 0.9101 | 1212.7230 | P48637 |
| ALVVDNGSGMCK | 0.9901 | 1365.6169 | Q562R1 |
| ALYPEGQAPVKK | 0.9532 | 1443.7874 | P37802 |
| ALYPEGQAPVKK | 0.8664 | 1443.7874 | P37802 |
| ALYPEGQAPVKK | 0.9874 | 1443.78 | P37802 |
| AMPTLIELMKDPSVVVR | 0.9529 | 2014.0743 | Q14974 |
| AMQLLTAEIEK | 0.9385 | 1361.7012 | Q07666 |
| ANAGPNTNGSQFFICTAK | 0.9924 | 2012.9163 | A2BFH1 |
| ANNLVAAAIDAR | 0.9037 | 1285.6526 | P11586 |
| ANTVLSGGTTMYPGIADR | 0.9934 | 1910.8867 | P53478 |
| APELIHDFLVNEK | 0.9998 | 1639.8358 | P53618 |
| APELIHDFLVNEK | 0.9994 | 1639.8358 | P53618 |
| APMLVTGNPGVPVPAAAAAAAQK | 0.9808 | 2217.1728 | Q9NR56 |
| APSGQPGSTK | 0.7451 | 1044.4914 | Q7VA20 |
| APTHFLVIPK | 0.9632 | 1237.6971 | P49773 |
| APVNVTTEVK | 0.9966 | 1172.6115 | P68103 |
| APVNVTTEVK | 0.9944 | 1172.6115 | P68103 |
| APVNVTTEVK | 0.9779 | 1172.6115 | P68103 |
| AQGHGIIQVDK | 0.8946 | 1280.6625 | P29144 |
| AQINQGESITHALK | 1.0000 | 1624.8321 | Q01518 |
| AQINQGESITHALK | 1.0000 | 1624.8321 | Q01518 |
| AQINQGESITHALK | 0.9982 | 1624.8321 | Q01518 |
| AQINQGESITHALK | 0.9973 | 1624.8247 | Q01518 |
| AQINQGESITHALK | 0.9976 | 1624.8247 | Q01518 |
| AQLDHWALTQR | 0.9909 | 1425.6901 | Q6GTX8 |
| ASAAAVDAGMAMAGQSPVLR | 0.7298 | 1960.9247 | P26599 |
| ASILAAFSK | 0.9047 | 1022.5549 | P54819 |
| ASILAAFSK | 0.9742 | 1022.5475 | P08166 |
| ASILAAFSK | 0.9910 | 1022.5475 | P08166 |
| ASQCQQPAENK | 0.9999 | 1375.5938 | Q01518 |
| ASQCQQPAENK | 0.9998 | 1375.5938 | Q01518 |

| **Identified Peptides (prime sequence)** | **PeptideProphet probability** | **Neutral peptide mass (Da)** | **Exemplary protein ID** |
| --- | --- | --- | --- |
| ASQCQQPAENK | 0.9973 | 1375.5938 | Q01518 |
| ASSSSLEK | 0.8969 | 923.4348 | P62736 |
| ASSSSLEKSYELPDGQVITIGNER | 0.9984 | 2695.3089 | P62736 |
| ASTPVFGGILSLINEHR | 1.0000 | 1897.9798 | O14773 |
| ATAASSSSLEK | 0.9265 | 1166.5567 | P62736 |
| ATAASSSSLEK | 0.9687 | 1166.5493 | P53478 |
| ATAASSSSLEK | 0.9407 | 1166.5493 | P53478 |
| ATAASSSSLEK | 0.9831 | 1166.5493 | P53478 |
| ATAASSSSLEK | 0.9845 | 1166.5493 | P53478 |
| ATAASSSSLEK | 0.9956 | 1166.5493 | P53478 |
| ATAASSSSLEK | 0.9602 | 1166.5493 | P53478 |
| ATAASSSSLEK | 0.9835 | 1166.5493 | P53478 |
| ATAASSSSLEK | 0.9931 | 1166.5493 | P53478 |
| ATAASSSSLEK | 0.909 | 1166.5493 | P53478 |
| ATQLAVNKIKE | 0.9939 | 1357.7717 | Q99832 |
| ATVLARSIAKE | 0.9766 | 1273.7142 | P10809 |
| ATYAPVISAEK | 0.9884 | 1264.6377 | P68362 |
| AVALAGLLAAQK | 0.9982 | 1240.7291 | P23368 |
| AVALAGLLAAQK | 0.8190 | 1240.7291 | P23368 |
| AVALAYGIYK | 0.9922 | 1183.6389 | O95757 |
| AVDALIDSMSLAK | 0.9998 | 1448.7333 | P13010 |
| AVIAELKK | 0.8011 | 1014.6225 | P10809 |
| AVLIVAKKCPS | 0.9822 | 1328.7638 | Q04323 |
| AVRLLLPGE | 0.9342 | 1054.5923 | Q96A08 |
| AVTVAPPGARQGQQQAGGDGKTE | 0.9997 | 2338.1414 | Q00839-2 |
| AVTYTEHAK | 0.9752 | 1134.5458 | P62805 |
| AVVFGPNLLWAK | 0.9989 | 1429.7870 | Q07960 |
| AWGLVTTAPR | 0.7969 | 1158.5934 | A6NCC3 |
| CAEHQINLIK | 0.9939 | 1340.6659 | P25398 |
| CAEHQINLIK | 0.9312 | 1340.6585 | Q76I81 |
| CAGYLEGGK | 0.9759 | 1069.4577 | P00760 |
| CAILSPAFK | 0.7828 | 1121.5691 | Q92598 |
| CALSTSQLVACTK | 0.7832 | 1553.7256 | P54939 |
| CAVLIVAAGVGEFEAGISK | 0.9997 | 2006.0217 | P68103 |
| CDEGYESGFMMMKNCMDIDECQ | 0.8594 | 2880.9926 | P35555 |
| CEDIIQLKPDVVITEK | 0.9815 | 2043.0710 | P49368 |
| CELINALYPEGQAPVKK | 0.7977 | 2073.0717 | P37802 |
| CELINALYPEGQAPVKK | 0.9936 | 2073.0717 | P37802 |
| CELINALYPEGQAPVKK | 0.9829 | 2073.0717 | P37802 |

| **Identified Peptides (prime sequence)** | **PeptideProphet probability** | **Neutral peptide mass (Da)** | **Exemplary protein ID** |
| --- | --- | --- | --- |
| CETIIGAVP | 0.8890 | 1046.4781 | P23400 |
| CGVDLIIGVGGGR | 0.9799 | 1342.6377 | B8GGP5 |
| CIAIKESAK | 0.9973 | 1162.6168 | P61158 |
| CLHFNPR | 0.9989 | 1030.4555 | P09382 |
| CPGESSHICDFIR | 0.9910 | 1647.6484 | P45478 |
| CPGESSHICDFIR | 0.9987 | 1647.6484 | P45478 |
| CVLQGLQTPSCK | 0.9992 | 1505.7044 | P13489 |
| CVVAVLPHILDTGAAGR | 0.9963 | 1835.9464 | Q15084 |
| CVVAVLPHILDTGAAGR | 0.9997 | 1835.9464 | Q15084 |
| DAANFEQFLQER | 0.8383 | 1554.6851 | P35268 |
| DAFGTAHR | 0.8651 | 961.4154 | P00558 |
| DAGAGIALNDHFVK | 0.9819 | 1542.7579 | P04406 |
| DAGAGIALNDHFVK | 0.9543 | 1542.7579 | P04406 |
| DAGAGIALNDHFVK | 0.9958 | 1542.7507 | P10096 |
| DAGAGIALNDHFVK | 0.9997 | 1542.7507 | P10096 |
| DAGAGIALNDHFVK | 0.9735 | 1542.7507 | P10096 |
| DAGILQLVESVR | 0.9993 | 1386.7255 | P13489 |
| DALCVLAQTVK | 0.9002 | 1332.686 | P78371 |
| DALDKIR | 0.9204 | 945.5032 | P14625 |
| DAMAGDFVNMVEK | 0.9996 | 1541.6642 | P10809 |
| DANLQTLTEYLKK | 0.9481 | 1679.8882 | P55060 |
| DANTIVCNSK | 0.8726 | 1236.5557 | P09382 |
| DANTIVCNSKDGGAWGTEQR | 0.9977 | 2294.0134 | P09382 |
| DANTIVCNSKDGGAWGTEQRE | 0.9995 | 2423.0560 | P09382 |
| DAPMFVMGVNHEK | 0.9848 | 1589.7119 | P04406 |
| DCHTAHIACK | 0.9933 | 1327.5550 | P68104 |
| DCHTAHIACK | 0.9887 | 1327.555 | P68104 |
| DCHTAHIACK | 0.9739 | 1327.5550 | P68104 |
| DDHDPVDK | 0.8588 | 1055.4308 | P22626 |
| DDVVGIVEIINSK | 0.9999 | 1515.7933 | Q01518 |
| DEELNKLLGK | 0.9704 | 1301.6979 | P20671 |
| DEGGFAPNILENKEGLELLK | 0.9961 | 2329.1953 | P06733 |
| DEITYVELQKEEAQK | 0.9768 | 1965.9683 | Q00839 |
| DEITYVELQKEEAQK | 0.9986 | 1965.9683 | Q00839 |
| DESGPSIVHR | 0.9369 | 1183.5370 | Q9BYX7 |
| DESGPSIVHR | 0.9893 | 1183.537 | Q9BYX7 |
| DESGPSIVHR | 0.9733 | 1183.5370 | Q9BYX7 |
| DESTGSIAKR | 0.9650 | 1178.5679 | P04075 |
| DFEQEMATAASSSSLEK | 0.9818 | 1945.8363 | P60709 |

| **Identified Peptides (prime sequence)** | **PeptideProphet probability** | **Neutral peptide mass (Da)** | **Exemplary protein ID** |
| --- | --- | --- | --- |
| DFLLKPELLR | 0.9300 | 1358.7710 | O00148 |
| DIAVDGEPLGR | 0.9029 | 1228.5836 | P62937 |
| DIAVDGEPLGR | 0.9126 | 1228.5836 | P62937 |
| DIETIGEILKK | 0.8970 | 1401.7867 | P61978 |
| DISPQAPTHFLVIPK | 0.7650 | 1777.9515 | P49773 |
| DISPQAPTHFLVIPK | 0.9442 | 1777.9515 | P49773 |
| DISPQAPTHFLVIPK | 0.9588 | 1777.9515 | P49773 |
| DIVQLPTGLTGIK | 0.8570 | 1469.8242 | P34932 |
| DKANAQAAALYK | 0.9998 | 1406.7306 | P40121 |
| DKANAQAAALYK | 0.9801 | 1406.7306 | P40121 |
| DKANAQAAALYK | 0.9953 | 1406.7306 | P40121 |
| DKDGDGTITTK | 0.9540 | 1293.6201 | P62158 |
| DKDGDGTITTKE | 0.9500 | 1422.6627 | P62158 |
| DKFDENAK | 0.9956 | 1109.5141 | P00558 |
| DKGLQTSQDAR | 0.9488 | 1333.6374 | P27797 |
| DKLNVITVGPR | 0.9931 | 1326.7408 | P04040 |
| DKLNVITVGPR | 0.9634 | 1326.7408 | P04040 |
| DKPLKDVIIAD | 0.8905 | 1369.7605 | P23284 |
| DKYLIPNATQPESK | 0.9966 | 1746.894 | P31946 |
| DKYLIPNATQPESK | 0.8435 | 1746.8940 | P31946 |
| DLCHALR | 0.9924 | 971.4395 | P24534 |
| DLFNAVGDGIVLCK | 0.9893 | 1635.8079 | P13796 |
| DLFNAVGDGIVLCK | 0.9393 | 1635.8079 | P13796 |
| DLVVGLCTGQIK | 0.9785 | 1417.7388 | P06733 |
| DMVPGKPMCVESFSDYPPLGR | 0.9984 | 2497.1228 | P68104 |
| DMVPGKPMCVESFSDYPPLGR | 0.9872 | 2497.1228 | P68104 |
| DMVPGKPMCVESFSDYPPLGR | 0.9988 | 2497.1228 | P68104 |
| DNDIMLIK | 0.9709 | 1076.5324 | P35030 |
| DNSSRPSQVVAETR | 0.8388 | 1632.7604 | P13639 |
| DNVICPGAPDFLAHVR | 0.9995 | 1867.8788 | P21964 |
| DQANLTVK | 0.7675 | 1003.5087 | P09382 |
| DQAQKAEGAGDAK | 0.8455 | 1431.6742 | P05204 |
| DQIQNAQYLLQNSVK | 0.9949 | 1876.9431 | P61978 |
| DQLHAAVGASR | 0.9783 | 1211.5795 | P13804 |
| DQSYKPDENEVR | 0.9939 | 1594.7011 | P31939 |
| DRTVIDYNGER | 0.9844 | 1424.6432 | P07237 |
| DRTVIDYNGERTLD | 0.9619 | 1753.8019 | P07237 |
| DSCTCAGSCKCKE | 0.9934 | 1705.6317 | P02795 |
| DSLLAGPVAEYLK | 0.9980 | 1490.7769 | Q01518 |

| **Identified Peptides (prime sequence)** | **PeptideProphet probability** | **Neutral peptide mass (Da)** | **Exemplary protein ID** |
| --- | --- | --- | --- |
| DSLLAGPVAEYLK | 0.7829 | 1490.7769 | Q01518 |
| DSLLAGPVAEYLK | 0.9357 | 1490.7769 | Q01518 |
| DSLLAGPVAEYLK | 0.7614 | 1490.7697 | Q01518 |
| DSLLAGPVAEYLK | 0.8376 | 1490.7697 | Q01518 |
| DSLLAGPVAEYLK | 0.9653 | 1490.7695 | Q01518 |
| DSLYVEKIDVGEAEPR | 0.8656 | 1934.9373 | P54577 |
| DSYVGDEAQSKR | 0.9955 | 1469.6535 | P62736 |
| DTFLEHMCR | 0.9732 | 1295.5175 | P14618 |
| DTFLEHMCR | 0.7707 | 1295.5175 | P14618 |
| DTFWKEFGTNIK | 0.8257 | 1628.7987 | Q58FF3 |
| DTKPGTTGSGAGSGGPGGLTSAAPAGGDKK | 0.9999 | 2728.3417 | P67809 |
| DTKPGTTGSGAGSGGPGGLTSAAPAGGDKK | 0.8637 | 2728.3417 | P67809 |
| DTKPGTTGSGAGSGGPGGLTSAAPAGGDKK | 0.9915 | 2728.3417 | P67809 |
| DTLLVDVEPK | 0.9029 | 1243.6448 | P62314 |
| DTYNCDLHFK | 0.9823 | 1427.5928 | Q9BUJ2 |
| DTYNCDLHFK | 0.8452 | 1427.5928 | Q9BUJ2 |
| DTYNCDLHFK | 0.9124 | 1427.5928 | Q9BUJ2 |
| DVCPLTLGIETVGGVMTK | 0.9905 | 2004.9937 | Q91883 |
| DVVVLPGGNLGAQNLSESAAVK | 1.0000 | 2253.1753 | Q99497 |
| DVVVLPGGNLGAQNLSESAAVK | 0.9864 | 2253.1753 | Q99497 |
| DVVVLPGGNLGAQNLSESAAVK | 0.9439 | 2253.1753 | Q99497 |
| DVVYALK | 0.9718 | 922.4912 | P62805 |
| DVVYALKR | 0.8879 | 1078.5923 | P62805 |
| DYNGHVGLGVK | 0.9845 | 1273.6203 | P15880 |
| DYNGHVGLGVK | 0.9561 | 1273.6203 | P15880 |
| EALAAAELLKK | 0.9997 | 1299.7550 | P29401 |
| EAPNPKL | 0.8195 | 883.4477 | Q39072 |
| EAPNPKL | 0.8939 | 883.4477 | Q39072 |
| EASEAYLVGLFEDTNLCAIHAK | 0.7360 | 2566.2162 | Q71DI3 |
| EASGGGAFLVLPLGK | 0.9999 | 1530.8117 | P31146 |
| EAYLVGLFEDTNLCAIHAK | 0.9475 | 2279.1044 | P68431 |
| EAYLVGLFEDTNLCAIHAK | 1.0000 | 2279.1044 | P68431 |
| EDTNLCAIHAK | 0.9986 | 1386.6350 | P68431 |
| EDTNLCAIHAK | 0.9858 | 1386.635 | P68431 |
| EDTNLCAIHAK | 0.9785 | 1386.6350 | P68431 |
| EDTNLCAIHAK | 0.9989 | 1386.6276 | P84227 |
| EDTNLCAIHAK | 0.9998 | 1386.6276 | P84227 |
| EDTNLCAIHAK | 0.9998 | 1386.6277 | P84227 |
| EDTNLCAIHAK | 0.9990 | 1386.6277 | P84227 |

| **Identified Peptides (prime sequence)** | **PeptideProphet probability** | **Neutral peptide mass (Da)** | **Exemplary protein ID** |
| --- | --- | --- | --- |
| EGNDYFKEK | 0.7990 | 1272.5775 | O95801 |
| EHGIQPDGQMPSDK | 0.9983 | 1653.7205 | Q71U36 |
| EIAPHALLQAVLK | 0.9996 | 1517.8718 | P49327 |
| ELDQDMVTEDEDDPG | 0.7924 | 1792.6297 | Q9NRL2 |
| ESCGIHETTFNSIMK | 0.9994 | 1868.8185 | P60709 |
| ESCGIHETTFNSIMK | 0.9991 | 1868.8185 | P60709 |
| ESHIQSTSDR | 0.7984 | 1246.5326 | Q14974 |
| EVAAAVPAPK | 0.9357 | 1049.5584 | Q82KE2 |
| EVGDIMLIR | 0.9609 | 1132.5699 | P00491 |
| EVGVLVGK | 0.9975 | 915.5178 | P07737 |
| EVLLPGLQK | 0.8901 | 1111.6389 | P07954 |
| FAALTSIAQK | 0.8735 | 1164.6291 | Q9Y5Y2 |
| FAEALAAHK | 0.8814 | 1072.5453 | P07237 |
| FAEALAAHK | 0.9990 | 1072.5453 | P07237 |
| FAEALAAHK | 0.9996 | 1072.5453 | P07237 |
| FAGILSQGLR | 0.9941 | 1148.6090 | P09874 |
| FAGPHAALANK | 0.9990 | 1211.6199 | Q9BY44 |
| FAGSVPPP | 0.7937 | 858.395 | P13002 |
| FALLEIPK | 0.8483 | 1045.5960 | O94915 |
| FAPVNVTTEVK | 0.9611 | 1319.6874 | P68104 |
| FAPVNVTTEVK | 0.9868 | 1319.6799 | P68103 |
| FAPVNVTTEVK | 0.9994 | 1319.6797 | P68103 |
| FAPVNVTTEVK | 0.8600 | 1319.6797 | P68103 |
| FAPVNVTTEVK | 0.9995 | 1319.6797 | P68103 |
| FAQINQGESITHALK | 0.9989 | 1771.9005 | Q01518 |
| FAQINQGESITHALK | 0.9986 | 1771.9005 | Q01518 |
| FAQINQGESITHALK | 0.9997 | 1771.9005 | Q01518 |
| FAQINQGESITHALK | 0.9962 | 1771.9005 | Q01518 |
| FAQINQGESITHALK | 0.9999 | 1771.8927 | Q01518 |
| FCAILHR | 0.8255 | 1003.4810 | Q8N3D4 |
| FCSEYRPK | 0.9160 | 1201.5338 | P09429 |
| FDQANLTVK | 0.8275 | 1150.5696 | P09382 |
| FDSLLAGPVAEYLK | 0.9904 | 1637.8377 | Q01518 |
| FDSLLAGPVAEYLK | 0.9966 | 1637.8377 | Q01518 |
| FEDTNLCAIHAK | 0.9996 | 1533.7034 | P68431 |
| FEDTNLCAIHAK | 0.9999 | 1533.7034 | P68431 |
| FEDTNLCAIHAK | 0.9998 | 1533.7034 | P68431 |
| FEDTNLCAIHAK | 0.9621 | 1533.6957 | P84227 |
| FEDTNLCAIHAK | 0.9551 | 1533.6957 | P84227 |

| **Identified Peptides (prime sequence)** | **PeptideProphet probability** | **Neutral peptide mass (Da)** | **Exemplary protein ID** |
| --- | --- | --- | --- |
| FEQEMATAASSSSLEK | 0.9996 | 1830.802 | P53478 |
| FEQEMATAASSSSLEK | 0.9994 | 1830.802 | P53478 |
| FFVQTCR | 0.9714 | 1044.4599 | B2RPK0 |
| FFVQTCREE | 0.9390 | 1302.5451 | B2RPK0 |
| FGGGVIGDLAGFAAANYLR | 0.7336 | 1955.9567 | Q1LU62 |
| FGILLDQGQLNK | 0.9081 | 1460.7776 | Q00610 |
| FGPDICGPGTK | 0.9951 | 1263.5632 | Q4VIT5 |
| FGTHETAFLGPK | 0.9962 | 1419.6935 | P51858 |
| FGVLGLDLWQVK | 0.9996 | 1489.8081 | P27797 |
| FGVLGLDLWQVK | 0.9581 | 1489.8007 | Q4VIT5 |
| FGYFEVTHDITK | 0.9994 | 1571.7409 | P04040 |
| FGYFEVTHDITK | 0.9974 | 1571.7337 | Q2I6W4 |
| FHTEQMYK | 0.8022 | 1198.5229 | P59998 |
| FIAIKPDGVQR | 0.8202 | 1358.7459 | P15531 |
| FIFIDSDHTDNQR | 0.9355 | 1694.7437 | P07237 |
| FIGAIAIGDLVK | 0.9927 | 1331.7601 | P78371 |
| FIGAIAIGDLVK | 0.9997 | 1331.7527 | Q3ZBH0 |
| FIGNSTAIQELFK | 0.9926 | 1582.8143 | Q13885 |
| FIGNSTAIQELFK | 0.9787 | 1582.8067 | Q9YHC3 |
| FIGNSTAIQELFKR | 0.8744 | 1738.9154 | Q13885 |
| FIGNSTAIQELFKR | 0.9711 | 1738.9154 | Q13885 |
| FILFKDAASVEK | 0.9951 | 1510.8184 | Q99729 |
| FIVLTTSAGIMDHEEAR | 0.9959 | 1976.9337 | Q9LX88 |
| FLAAGLK | 0.8607 | 834.4752 | Q9ULV1 |
| FLAQLKDECPEVR | 0.9664 | 1719.8402 | P30153 |
| FLGMESCGIHETTFNSIMK | 1.0000 | 2317.0329 | P60709 |
| FLGMESCGIHETTFNSIMK | 0.9998 | 2317.0329 | P60709 |
| FLLNTLQENVNK | 0.9991 | 1547.8096 | Q9HAV4 |
| FLLPHPGLQVATSPDFDGK | 0.9734 | 2154.0898 | Q6DD88 |
| FLLPHPGLQVATSPDFDGK | 0.9852 | 2154.0898 | Q6DD88 |
| FLNLANDPTIER | 0.9938 | 1489.7313 | P15313 |
| FLPEFLVSTQK | 0.9975 | 1423.7500 | P30740 |
| FLPEFLVSTQK | 0.7975 | 1423.75 | P30740 |
| FLPVIGLVDAEK | 0.9976 | 1415.7812 | P17980 |
| FLPVIGLVDAEK | 0.9904 | 1415.7812 | P17980 |
| FLTTGVLSTLR | 0.7258 | 1294.7033 | P49915 |
| FMNTELAAFTK | 0.8877 | 1387.6594 | P31949 |
| FMPGFAPLTSR | 0.9956 | 1310.6156 | Q9YHC3 |
| FMVVNDAGRPK | 0.9649 | 1348.671 | P11142 |

| **Identified Peptides (prime sequence)** | **PeptideProphet probability** | **Neutral peptide mass (Da)** | **Exemplary protein ID** |
| --- | --- | --- | --- |
| FMVVNDAGRPK | 0.9975 | 1348.6710 | P11142 |
| FMVVNDAGRPK | 0.9924 | 1348.6710 | P11142 |
| FNTLQTK | 0.7459 | 966.4923 | P12814 |
| FNVINGGSHAGNK | 0.9996 | 1429.6851 | P06733 |
| FPRPVTVEPMDQLDDEEGLPEK | 0.7247 | 2656.2479 | Q15233 |
| FQLAPAILQGQTK | 0.7493 | 1529.8354 | Q96JB5 |
| FSAPKPQTSPSPK | 0.9924 | 1514.7881 | Q01518 |
| FSGLFGGSSK | 0.9399 | 1101.5243 | P54920 |
| FSTPLLLGKK | 0.9838 | 1246.7437 | P40926 |
| FSTPLLLGKK | 0.8605 | 1246.7437 | P40926 |
| FTTTAERE | 0.9329 | 1041.4515 | Q562R1 |
| FTVWDVGGQDK | 0.8143 | 1366.6306 | P84077 |
| FVALSTNTTKVKE | 0.8709 | 1580.8562 | P06744 |
| FVLDEFKR | 0.7413 | 1168.6029 | P26641 |
| FVMGVNHEK | 0.9932 | 1175.5546 | P04406 |
| FVTFCTK | 0.9301 | 1017.4742 | O60506 |
| FVTFDDHDPVDK | 0.9906 | 1549.6838 | P22626 |
| FVTFDDHDPVDK | 0.9648 | 1549.6838 | P22626 |
| FVVEVIK | 0.8488 | 948.5433 | Q07021 |
| FYELSENDLNFIK | 0.9989 | 1746.8253 | P13639 |
| FYFDPLINPISHR | 0.8857 | 1705.8365 | Q6P2Q9 |
| FYVNGLTLGGQK | 0.8321 | 1411.7248 | P07737 |
| FYVNGLTLGGQK | 0.9129 | 1411.7248 | P07737 |
| FYVNGLTLGGQK | 0.9978 | 1411.7248 | P07737 |
| FYVNGLTLGGQK | 0.9839 | 1411.7248 | P07737 |
| GAAAAIEAAAK | 0.9935 | 1058.5508 | Q9Y490 |
| GAAGVMAIEHADFAGVER | 0.9987 | 1887.8686 | P78371 |
| GAAVQAAILSGDK | 0.9963 | 1315.6884 | P11142 |
| GAAVSAGHGLPAK | 0.9992 | 1250.6520 | O75367 |
| GAFLHIK | 0.9826 | 900.4970 | Q8WUM4 |
| GAGLMGAGIAQVSVDK | 0.9985 | 1588.7957 | P40939 |
| GAGLMGAGIAQVSVDKGLK | 0.9483 | 1915.0349 | P40939 |
| GAGNPVGDKLNVITVGPR | 0.9997 | 1879.0064 | P04040 |
| GALAIANFAR | 0.9940 | 1090.5671 | P52306 |
| GALLEEAEQLLDR | 0.9389 | 1543.7556 | P48643 |
| GALLPCEECSGQLVFK | 0.9964 | 1922.8944 | P18493 |
| GAPFLKEGASEEEIR | 0.8969 | 1747.8529 | P23141 |
| GASGGAYEHR | 0.9997 | 1091.4532 | P31943 |
| GASGGAYEHR | 0.9912 | 1091.4532 | P31943 |

| **Identified Peptides (prime sequence)** | **PeptideProphet probability** | **Neutral peptide mass (Da)** | **Exemplary protein ID** |
| --- | --- | --- | --- |
| GASIYIENKEEK | 0.9465 | 1523.7619 | O75832 |
| GASTGIYEALELR | 0.9981 | 1466.7079 | Q9C9C4 |
| GDDDSGPGPK | 0.8628 | 1059.4257 | Q13316 |
| GDLDETSSNEGGVK | 0.7461 | 1522.6457 | Q9SLF3 |
| GDRFTDEEVDELYR | 0.9128 | 1830.7809 | P19105 |
| GDRFTDEEVDELYR | 0.9892 | 1830.7734 | P19105 |
| GDRFTDEEVDELYR | 0.9886 | 1830.7737 | P19105 |
| GEKPVGSLAGIGEVLGK | 0.9924 | 1753.9726 | O75531 |
| GESITHALK | 0.9755 | 1070.5508 | Q01518 |
| GESITHALK | 0.9604 | 1070.5508 | Q01518 |
| GFPCNQFGHQENAK | 0.9998 | 1748.7477 | P07203 |
| GFSAFPFELLHTPEK | 0.9999 | 1834.9042 | P07099 |
| GFSAFPFELLHTPEK | 0.9911 | 1834.9042 | P07099 |
| GGPLPPHLALK | 0.8714 | 1214.6924 | Q08211 |
| GGQDILSMMGQLMKPK | 0.9999 | 1876.9361 | Q9Y265 |
| GGQDILSMMGQLMKPK | 0.9995 | 1876.9361 | Q9Y265 |
| GGSHAGNKLAMQE | 0.9427 | 1414.6411 | P06733 |
| GGSSEPCALCSLHSIGK | 0.8927 | 1874.8403 | P14174 |
| GGSSEPCALCSLHSIGK | 0.8867 | 1874.8403 | P14174 |
| GGTTMYPGIADR | 0.9183 | 1325.5822 | P62736 |
| GGTTMYPGIADRMQKE | 0.9909 | 1869.8501 | P62736 |
| GGTTMYPGIGER | 0.8812 | 1325.5748 | P26183 |
| GGTTMYPGIGER | 0.9474 | 1325.5748 | P26183 |
| GGVCEPLK | 0.8395 | 974.4644 | Q9UHF7 |
| GGVLPNIQAVLLPK | 1.0000 | 1533.9031 | Q96QV6 |
| GGVLPNIQAVLLPK | 0.9999 | 1533.9031 | Q96QV6 |
| GGVLPNIQAVLLPK | 0.9530 | 1533.8957 | P04908 |
| GGVLPNIQAVLLPK | 0.9907 | 1533.8957 | P04908 |
| GGVLPNIQAVLLPK | 0.9987 | 1533.8957 | P04908 |
| GGVLPNIQAVLLPK | 0.9987 | 1533.8957 | P04908 |
| GGVLPNIQAVLLPK | 0.9920 | 1533.8957 | P04908 |
| GGVMSGAVPAAAAQEAVEEDIPIAK | 0.7462 | 2496.2318 | P52815 |
| GHILAAEQLSR | 0.8893 | 1281.6577 | Q15738 |
| GHLLLVAK | 0.9794 | 965.5810 | Q9BX68 |
| GHPAFVNYSTSQK | 0.9998 | 1550.7266 | P14866 |
| GHPLGASGCR | 0.9845 | 1098.4777 | Q9BWD1 |
| GHSVEELCK | 0.9973 | 1173.5236 | P29401 |
| GHVGADLAALCSEAALQAIR | 0.9999 | 2110.0377 | P55072 |
| GHVLAAGCGQNPVR | 0.9999 | 1522.7211 | Q9BWD1 |

| **Identified Peptides (prime sequence)** | **PeptideProphet probability** | **Neutral peptide mass (Da)** | **Exemplary protein ID** |
| --- | --- | --- | --- |
| GHVLAAGCGQNPVR | 0.9996 | 1522.7211 | Q9BWD1 |
| GHVLAAGCGQNPVR | 0.9924 | 1522.7211 | Q9BWD1 |
| GIFVEKYDPTIEDSYR | 0.9973 | 2046.9687 | P62834 |
| GIHETTFNSIMK | 0.9991 | 1492.7057 | P53478 |
| GIHETTFNSIMK | 0.9998 | 1492.7057 | P53478 |
| GIHETTFNSIMK | 0.9997 | 1492.7057 | P53478 |
| GIHETTFNSIMK | 0.9990 | 1492.7057 | P53478 |
| GIHETTFNSIMK | 0.9998 | 1492.7058 | P53478 |
| GIHETTFNSIMK | 0.9523 | 1492.7132 | Q562R1 |
| GIHETTFNSIMK | 0.9967 | 1492.7132 | Q562R1 |
| GIHETTFNSIMK | 0.9343 | 1492.7132 | Q562R1 |
| GIMNSFVNDIFER | 0.9978 | 1628.7327 | P06900 |
| GIMNSFVNDIFER | 0.9998 | 1628.7327 | P06900 |
| GIPVLVLGNKR | 0.9742 | 1280.7717 | Q96BM9 |
| GIPVLVLGNKR | 0.9663 | 1280.7717 | Q96BM9 |
| GIPYLDAPSEAEASCAALVK | 0.9998 | 2177.0462 | P39748 |
| GISLANLLLSK | 0.9304 | 1243.7288 | P08397 |
| GISQGLADNTVIAK | 0.9995 | 1501.7888 | P26639 |
| GITLPVDFQGR | 0.9878 | 1289.6442 | P31943 |
| GITLPVDPEGK | 0.8388 | 1240.6377 | Q5E9J1 |
| GIVPIVEPEILPDGDHDLK | 0.9999 | 2171.1262 | P04075 |
| GLAWSKTGPVAKE | 0.9915 | 1486.7932 | Q01518 |
| GLDLWQVK | 0.9439 | 1073.5658 | P27797 |
| GLEVFHAGTALK | 0.9976 | 1357.7142 | P22102 |
| GLFPCVDELSDIHTR | 0.9869 | 1845.8468 | Q92974 |
| GLIFVVDSNDR | 0.8725 | 1321.6415 | P84077 |
| GLLWALEPEKPLVR | 0.9991 | 1735.9773 | Q86TX2 |
| GLLWALEPEKPLVR | 0.9986 | 1735.9773 | Q86TX2 |
| GLTHTAVVPLDLVK | 0.8956 | 1577.8929 | Q00325-2 |
| GLTLGGQKCSVIRD | 0.9984 | 1618.8249 | P07737 |
| GLVASNLNLKPGECLR | 0.9770 | 1855.9726 | P09382 |
| GMGMEGIGFGINK | 0.9787 | 1425.6533 | P52272 |
| GMILPTMNGESVDPVGQPALK | 0.9517 | 2269.1235 | O95433 |
| GNIVGLVGVDQFLVK | 0.8504 | 1672.9227 | Q3SYU2 |
| GNIVGLVGVDQFLVK | 0.9997 | 1672.9227 | Q3SYU2 |
| GNIVGLVGVDQFLVK | 0.9982 | 1672.9227 | Q3SYU2 |
| GPKPALPAGTEDTAK | 1.0000 | 1595.8307 | P06396 |
| GPKPALPAGTEDTAKEDAANR | 0.8412 | 2252.1185 | P06396 |
| GQDEMIDVIGVTK | 0.8514 | 1519.7341 | P39023 |

| **Identified Peptides (prime sequence)** | **PeptideProphet probability** | **Neutral peptide mass (Da)** | **Exemplary protein ID** |
| --- | --- | --- | --- |
| GQKDSYVGDEAQSK | 0.9999 | 1654.7587 | P62736 |
| GQLLTSSNYDDDEKK | 0.9999 | 1855.8588 | P11388 |
| GQLLTSSNYDDDEKK | 0.9998 | 1855.8588 | P11388 |
| GQLLTSSNYDDDEKK | 0.9476 | 1855.8588 | P11388 |
| GQLMNMLSHPVIR | 0.9825 | 1582.7860 | Q7Z6Z7 |
| GQSGAGNNWAK | 0.9963 | 1204.5373 | Q13885 |
| GQSGAGNNWAK | 0.9661 | 1204.5373 | Q13885 |
| GQSGAGNNWAK | 0.8827 | 1204.5299 | Q9YHC3 |
| GQVITIGNER | 0.9861 | 1173.5890 | P62736 |
| GSLGQGLGAACGMAYTGK | 0.9989 | 1813.8239 | P29401 |
| GSPKADSPGSLTI | 0.7936 | 1344.6673 | Q8NFW5 |
| GSTSDLGHCEK | 0.9672 | 1305.5408 | P22234 |
| GSTSDLGHCEK | 0.9868 | 1305.5408 | P22234 |
| GTAAVALAGLLAAQK | 0.9999 | 1469.8354 | P23368 |
| GTFALNLLK | 0.9662 | 1091.6127 | P35237 |
| GTQDQIQNAQYLLQNSVK | 0.9924 | 2163.0708 | P61978 |
| GVDLLADAVAVTMGPK | 0.9054 | 1671.8654 | P10809 |
| GVDLLADAVAVTMGPK | 0.9908 | 1671.8577 | P10809 |
| GVGYLAGCLVHALGEK | 0.9997 | 1758.8875 | Q9Y3Z3 |
| GVGYLAGCLVHALGEK | 0.9992 | 1758.8875 | Q9Y3Z3 |
| GVHQVPTENVQVHFTER | 0.9582 | 2063.9926 | Q9HB71 |
| GVLPNIQAVLLPK | 0.8899 | 1476.8816 | Q96QV6 |
| GVPMPDKYSLEPVAVELK | 0.9709 | 2115.1074 | P00558 |
| GVPMPDKYSLEPVAVELK | 0.8261 | 2115.1074 | P00558 |
| GVSHPVLK | 0.8490 | 951.5290 | P22695 |
| GVSLAVCK | 0.9987 | 948.4851 | P06733 |
| GVSLAVCK | 0.8683 | 948.4851 | P06733 |
| GVSLQELNPEMGTDNDSENWK | 1.0000 | 2478.0757 | P07195 |
| GVVVLHK | 0.9855 | 866.5126 | O43395 |
| GWVAMAPKPGPYVK | 0.8966 | 1643.8646 | Q01518 |
| HACIGGTNVR | 0.7667 | 1171.5305 | P60842 |
| HAQVADMK | 0.9908 | 1014.4705 | P35579 |
| HCASPPPSSNNK | 0.9983 | 1410.6098 | Q15738 |
| HCIDPNDSK | 0.9925 | 1200.4982 | Q15185 |
| HEALAAAELLKK | 0.9990 | 1436.8139 | P29401 |
| HEALAAAELLKK | 0.9717 | 1436.8139 | P29401 |
| HEKYDNSLK | 0.8571 | 1276.6200 | P04406 |
| HHTFYNELR | 0.8326 | 1303.5846 | Q9BYX7 |
| HIISSNLEK | 0.9587 | 1155.6036 | Q9UL46 |

| **Identified Peptides (prime sequence)** | **PeptideProphet probability** | **Neutral peptide mass (Da)** | **Exemplary protein ID** |
| --- | --- | --- | --- |
| HILSPWGAEVK | 0.9009 | 1351.7037 | P09874 |
| HLATGDMLR | 0.9014 | 1100.5185 | P54819 |
| HLATGDMLR | 0.9927 | 1100.5185 | P54819 |
| HLKSPVR | 0.8036 | 951.5402 | Q9UIF9 |
| HLLLQNNLPAVR | 0.9991 | 1474.8156 | P48643 |
| HLQLAIRNDEE | 0.9998 | 1424.6796 | Q96QV6 |
| HNCAVEFNFGQK | 0.9986 | 1565.6833 | Q00839 |
| HQALLGTIR | 0.9985 | 1095.5937 | P25705 |
| HQATILPK | 0.9370 | 1022.5661 | P49327 |
| HQATILPK | 0.9952 | 1022.5661 | P49327 |
| HQGVMVGMGQKDSYVGDE | 0.9993 | 2051.8829 | P62736 |
| HSFGGGTGSGFTSLLMER | 1.0000 | 1927.8635 | Q71U36 |
| HSGIAPR | 0.7955 | 824.4041 | Q8IWT3 |
| HSIIETLR | 0.9792 | 1055.5512 | P07900 |
| HSIVLPLK | 0.8109 | 1021.6072 | O94979 |
| HSLGGGTGSGMGTLLISK | 0.8678 | 1787.8988 | Q13885 |
| HSLGGGTGSGMGTLLISK | 0.9789 | 1787.8988 | Q13885 |
| HSLLPALCDSK | 0.7387 | 1355.6581 | A1YES6 |
| HTISPLDLAK | 0.9779 | 1209.6506 | Q15365 |
| HTVLPEALER | 0.9130 | 1251.6286 | Q3B7M9 |
| HTVPIYEGYALPHAILR | 0.7847 | 2037.0584 | P60709 |
| HVTYAGAAVDELGK | 0.9999 | 1545.7576 | P30086 |
| HVVNIGAEDLK | 0.9985 | 1309.6778 | P13796 |
| IAAQYSGAQVR | 0.9993 | 1250.6156 | P26641 |
| IADLVVGLCTGQIK | 0.9561 | 1601.8599 | P06733 |
| IAGHPAFVNYSTSQK | 0.9148 | 1734.8478 | P14866 |
| IAIGDLVK | 0.8966 | 943.5491 | P78371 |
| IANLFNR | 0.9812 | 934.4773 | P13796 |
| IAPALVSKKLNVTE | 0.8933 | 1625.9504 | P06733 |
| IAPALVSKKLNVTE | 0.9997 | 1625.9504 | P06733 |
| IAPIVIFASNR | 0.9998 | 1287.7087 | Q9Y265 |
| IAQGGVLPNIQAVLLPK | 1.0000 | 1846.0828 | Q96QV6 |
| IAQGGVLPNIQAVLLPK | 0.9991 | 1846.0757 | P04908 |
| IAQGGVLPNIQAVLLPK | 0.9981 | 1846.0757 | P04908 |
| IASGGVLPNIHPELLAK | 0.9989 | 1844.0308 | O75367 |
| IASGGVLPNIHPELLAK | 0.9897 | 1844.0308 | O75367 |
| ICAGPTALLAHEIGFGSK | 0.7868 | 1956.988 | Q99497 |
| ICQQNGIVPIVEPEILPDGDHDLK | 0.9405 | 2814.4010 | P04075 |
| ICQQNGIVPIVEPEILPDGDHDLK | 0.9995 | 2814.401 | P04075 |

| **Identified Peptides (prime sequence)** | **PeptideProphet probability** | **Neutral peptide mass (Da)** | **Exemplary protein ID** |
| --- | --- | --- | --- |
| IFIDSDHTDNQR | 0.9968 | 1547.6753 | P07237 |
| IFIDSDHTDNQR | 0.9920 | 1547.6753 | P07237 |
| IFIDSDHTDNQR | 0.9775 | 1547.6679 | P05307 |
| IGAIAIGDLVK | 0.9970 | 1184.6917 | P78371 |
| IGNSTAIQELFKR | 0.9922 | 1591.8470 | Q13885 |
| IGRRFDDAVVQSD | 0.9438 | 1564.7382 | P11142 |
| IGSLICNVGAGGPAPAAGAAPAGGPAPSTAAAPAEEK | 0.9994 | 3341.6462 | P05386 |
| IGYPITLFVEK | 0.9997 | 1394.7598 | Q14568 |
| IGYPITLYLEK | 0.9793 | 1424.7703 | Q58FF7 |
| IIAEGIPEALTR | 0.8924 | 1369.7353 | P53396 |
| IINSLYKNKE | 0.8141 | 1364.7452 | P14625 |
| IIRPRPPK | 0.7241 | 1091.6716 | Q16881-2 |
| IKKIGYNPD | 0.8868 | 1190.6447 | P68104 |
| ILGQNGISDLVK | 0.9844 | 1371.751 | P00338 |
| ILGQNGISDLVK | 0.8321 | 1371.7437 | P00338 |
| ILGQNGISDLVK | 0.9640 | 1371.7436 | P00338 |
| ILGQNGISDLVK | 0.7707 | 1371.7437 | P00338 |
| ILGTTLKDEGK | 0.9996 | 1317.7292 | O75083 |
| ILGTTLKDEGK | 0.8954 | 1317.7292 | O75083 |
| ILLVQPTKRPE | 0.9959 | 1408.8190 | P84090 |
| ILNVSAVDKSTGKE | 0.9953 | 1603.8569 | P11142 |
| ILTHGIFSGPAISR | 0.9878 | 1555.8259 | P60891 |
| ILTLKYPIE | 0.9520 | 1204.6855 | P62736 |
| IMNSFVNDIFER | 0.9996 | 1571.7117 | P06900 |
| IMNSFVNDIFER | 0.9972 | 1571.7117 | P06900 |
| IMNSFVNDIFER | 0.9804 | 1571.7117 | P06900 |
| IMNSFVNDIFER | 0.8862 | 1571.7117 | P06900 |
| INGHNAEVR | 0.9906 | 1096.5162 | P22626 |
| IPNEIIHALQAGR | 0.9915 | 1518.8055 | P52272 |
| IPTEGGDFNEFPVPEQFK | 0.9989 | 2166.0058 | Q01518 |
| IPTLITQLTQK | 0.9882 | 1370.7921 | P55060 |
| IQANPLLEAFGNAK | 0.9970 | 1600.8361 | Q9UKX3 |
| IQAVLLPK | 0.7468 | 996.6120 | Q96QV6 |
| IQAVLLPK | 0.8108 | 996.6120 | Q96QV6 |
| IQAVLLPK | 0.9811 | 996.6120 | Q96QV6 |
| IQAVLLPK | 0.9327 | 996.612 | Q96QV6 |
| IQAVLLPKKTE | 0.9975 | 1382.8285 | Q96QV6 |
| IQGITKPAIR | 0.9891 | 1211.7138 | P62805 |
| IQWITTQCR | 0.9828 | 1292.6084 | P37802 |

| **Identified Peptides (prime sequence)** | **PeptideProphet probability** | **Neutral peptide mass (Da)** | **Exemplary protein ID** |
| --- | --- | --- | --- |
| IRNDEELNK | 0.9718 | 1245.6101 | Q96QV6 |
| ISADIETIGEILKK | 0.9999 | 1672.9399 | P61978 |
| ISADIETIGEILKK | 0.9899 | 1672.9399 | P61978 |
| ISADIETIGEILKK | 0.8749 | 1672.9399 | P61978 |
| ISATLPHEILEMTNK | 0.9952 | 1811.9239 | P38919 |
| ISKLIFKS | 0.8192 | 1078.6539 | Q9ULI3 |
| ISLCQAILDETKGDYEK | 0.9997 | 2126.0354 | P04083 |
| ISLCQAILDETKGDYEK | 0.9999 | 2126.0354 | P04083 |
| ISPYFINTSKGQKCE | 0.9980 | 1914.9298 | P10809 |
| ISRMQYAPNTQVE | 0.9635 | 1623.7463 | P40121 |
| ITALHIK | 0.9859 | 910.5388 | P62263 |
| ITFDQANLTVK | 0.8732 | 1364.7088 | P09382 |
| ITFDQANLTVK | 0.9371 | 1364.7017 | P09382 |
| ITGKTFSSR | 0.8278 | 1111.5774 | O15144 |
| ITWIGENVSGLQR | 0.9387 | 1559.7844 | Q14019 |
| ITYTDEEPVKK | 0.9329 | 1465.7453 | O14979 |
| IVASKASLRE | 0.9035 | 1188.6614 | P13489 |
| IVCNSKDGGAWGTEQRE | 0.9993 | 2021.9013 | P09382 |
| IVGNSALK | 0.7209 | 916.5130 | Q9Y283 |
| IVPALEIANAHR | 0.9979 | 1390.7469 | P10809 |
| IVPALEIANAHR | 0.9220 | 1390.7469 | P10809 |
| IVPALEIANAHR | 0.9801 | 1390.7397 | P10809 |
| IVPALEIANAHR | 0.9661 | 1390.7395 | P10809 |
| IVPALEIANAHR | 0.9979 | 1390.7397 | P10809 |
| IVPIVEPEILPDGDHDLK | 0.9998 | 2114.1048 | P04075 |
| IVPIVEPEILPDGDHDLK | 0.9759 | 2114.1048 | P04075 |
| IVPTGKTGLIIGKGGE | 0.9981 | 1682.9719 | Q96AE4 |
| IVSWGSGCAQK | 0.7351 | 1307.6006 | P00760 |
| IVSWGSGCAQK | 0.9912 | 1307.6006 | P00760 |
| IVVIGHVDSGK | 0.9998 | 1238.6771 | P68104 |
| IYTNYEAGKDDYVK | 0.9554 | 1821.8573 | P09211 |
| KADGIVSKNF | 0.8101 | 1221.6506 | P63220 |
| KANAQAAALYK | 0.9593 | 1291.7036 | P40121 |
| KAPLDIPVPDPVKE | 0.8980 | 1660.9188 | Q06323 |
| KDDAMLLK | 0.9173 | 1076.5688 | P10809 |
| KDSPSVWAAVPGK | 0.9997 | 1484.7776 | P07737 |
| KDSTLIMQLLR | 0.9913 | 1432.7860 | P31946 |
| KEPAVLELEGK | 0.9973 | 1355.7374 | Q01518 |
| KEPISVSSEQVLK | 0.9982 | 1586.8668 | P00918 |

| **Identified Peptides (prime sequence)** | **PeptideProphet probability** | **Neutral peptide mass (Da)** | **Exemplary protein ID** |
| --- | --- | --- | --- |
| KGTVAVQEK | 0.8271 | 1102.6135 | Q8NHV4 |
| KLAPGELTIIL | 0.7571 | 1282.7648 | P04114 |
| KNNQITNNQR | 0.9946 | 1344.6646 | P00558 |
| KPGMVVTFAPVNVTTEVK | 0.9903 | 2060.1129 | P68104 |
| KPIIDLYEEMGK | 0.9718 | 1578.8115 | P30085 |
| KPISVEGSSK | 0.8588 | 1174.6346 | Q9HB71 |
| KPMCVESFSDYPPLGR | 0.9997 | 1997.9127 | P68104 |
| KQDLPNAMNAAEITDK | 0.9861 | 1901.9305 | P84077 |
| KQGQDNLSSVKE | 0.9982 | 1475.7368 | P30040 |
| KSESEILR | 0.7890 | 1076.5614 | Q05823 |
| KVEFLECSAK | 0.9876 | 1353.6751 | Q9Y5M8 |
| LAAAELLKK | 0.9956 | 1099.6753 | P29401 |
| LAALGGNSSPSAKD | 0.8290 | 1402.6840 | P05387 |
| LAALGGNSSPSAKD | 0.9960 | 1402.6840 | P05387 |
| LAASALPALVMSK | 0.9913 | 1386.7693 | P36578 |
| LAAVGLVGDLCR | 0.9969 | 1330.6737 | Q14974 |
| LACNIALDAVK | 0.8981 | 1302.6754 | P49368 |
| LADNVICPGAPDFLAHVR | 0.9244 | 2051.9999 | P21964 |
| LAGGIIGVK | 0.9751 | 942.5650 | P61978 |
| LAGLATDVQTVAQR | 0.9988 | 1529.7950 | P49720 |
| LAGPVAEYLK | 0.9981 | 1175.6338 | Q01518 |
| LAGPVAEYLK | 0.8948 | 1175.6338 | Q01518 |
| LAGPVAEYLK | 0.9987 | 1175.6338 | Q01518 |
| LAGPVAEYLK | 0.9994 | 1175.6338 | Q01518 |
| LAGPVAEYLK | 0.9601 | 1175.6264 | Q01518 |
| LAGPVAEYLK | 0.9645 | 1175.6264 | Q01518 |
| LAHILSPWGAEVK | 0.9888 | 1535.8248 | P09874 |
| LAHILSPWGAEVK | 0.9823 | 1535.8248 | P09874 |
| LAIIDPGDSDIIR | 0.9914 | 1484.7623 | P62888 |
| LAIIDPGDSDIIR | 0.8758 | 1484.7623 | P62888 |
| LAIVEALNGK | 0.8556 | 1142.6447 | Q99497 |
| LAIVEALNGKEVAAQVK | 0.9999 | 1896.0832 | Q99497 |
| LANHSLR | 0.9446 | 897.4569 | P13489 |
| LANSLACQGKYTPSGQAGAAASE | 0.9993 | 2367.0913 | P04075 |
| LAPLPPLPAQFK | 0.8083 | 1406.8074 | Q9NP79 |
| LAPVNIFK | 0.7956 | 1016.5807 | P78371 |
| LAQYLINAR | 0.9931 | 1148.6090 | Q15365 |
| LASYAVQSK | 0.9245 | 1081.5556 | P26038 |
| LATYAPVISAEK | 0.9423 | 1377.7217 | P68362 |

| **Identified Peptides (prime sequence)** | **PeptideProphet probability** | **Neutral peptide mass (Da)** | **Exemplary protein ID** |
| --- | --- | --- | --- |
| LATYAPVISAEK | 0.9646 | 1377.7217 | P68362 |
| LATYAPVISAEK | 0.9575 | 1377.7217 | P68362 |
| LAVAVGHVK | 0.9947 | 1008.5869 | P62906 |
| LAVDAVIAELK | 0.9999 | 1256.7128 | P10809 |
| LAVDAVIAELK | 1.0000 | 1256.7128 | P10809 |
| LAVDAVIAELK | 0.9984 | 1256.7128 | P10809 |
| LAVDAVIAELK | 0.9916 | 1256.7054 | P10809 |
| LAVDAVIAELKK | 0.9999 | 1412.8391 | P10809 |
| LAVDAVIAELKK | 0.9998 | 1412.8391 | P10809 |
| LAVDAVIAELKK | 0.9994 | 1412.8391 | P10809 |
| LAVDAVIAELKK | 0.8569 | 1412.8317 | P10809 |
| LAVDAVIAELKK | 0.9021 | 1412.8317 | P10809 |
| LAVDAVIAELKK | 0.9383 | 1412.8317 | P10809 |
| LAVLQQFK | 0.9874 | 1061.6022 | O60506 |
| LCAIHAK | 0.9969 | 927.4748 | P68431 |
| LCAIHAK | 0.8768 | 927.4748 | P68431 |
| LCKPEPELNAAIPSANPAK | 0.9960 | 2163.1146 | Q8WUM4 |
| LEGGKQPR | 0.8731 | 999.5250 | Q9UKZ4 |
| LGALTLPLAR | 0.9873 | 1111.6501 | Q9BSJ8 |
| LGALTPMPAVR | 0.7633 | 1228.6386 | Q9UHC9 |
| LGGPEAAKSDETAAK | 0.9994 | 1587.7892 | P04792 |
| LGIPFAKPPLGPLR | 0.8858 | 1590.9398 | P23141 |
| LGSLALYEK | 0.9909 | 1108.5916 | P36542 |
| LGSLALYEK | 0.9095 | 1108.5842 | P36542 |
| LIANGPTGPVSF | 0.9292 | 1259.6224 | C7G046 |
| LINIIPEDHIPLNLSGK | 0.9754 | 2001.1047 | O95602 |
| LIPHDFGMK | 0.7430 | 1172.5800 | P09874 |
| LIQTADQLR | 0.9695 | 1144.5988 | P18031 |
| LISAGLPPLK | 0.8619 | 1123.6753 | O43143 |
| LISFGAAGPPR | 0.9936 | 1172.6090 | Q75VX8 |
| LISVYSEKGESSGK | 0.9401 | 1626.8253 | P36578 |
| LITNFHTEQMYK | 0.9897 | 1639.7816 | P59998 |
| LIVLEGVDR | 0.9916 | 1100.5978 | P23919 |
| LIVLEGVDR | 0.9763 | 1100.5978 | P23919 |
| LIYTNYEAGKDDYVK | 0.9982 | 1934.9414 | P09211 |
| LIYTNYEAGKDDYVK | 0.9990 | 1934.9414 | P09211 |
| LIYTNYEAGKDDYVK | 0.9993 | 1934.9414 | P09211 |
| LIYTNYEAGKDDYVK | 0.9975 | 1934.9337 | P09211 |
| LIYTNYEAGKDDYVK | 0.9992 | 1934.9337 | P09211 |

| **Identified Peptides (prime sequence)** | **PeptideProphet probability** | **Neutral peptide mass (Da)** | **Exemplary protein ID** |
| --- | --- | --- | --- |
| LKEDQTEYLEER | 0.9785 | 1667.7790 | Q58FF7 |
| LKQGQDNLSSVKE | 0.9061 | 1588.8209 | P30040 |
| LLAAEFLK | 0.9988 | 1019.5803 | Q99832 |
| LLAAEFLK | 0.9987 | 1019.5803 | Q99832 |
| LLAALGGNSSPSAKD | 0.9988 | 1515.7681 | P05387 |
| LLAALGGNSSPSAKD | 0.9889 | 1515.7681 | P05387 |
| LLAGIECPR | 0.9662 | 1115.5545 | Q7KZF4 |
| LLAGPVAEYLK | 0.9447 | 1288.7179 | Q01518 |
| LLAGPVAEYLK | 0.9722 | 1288.7179 | Q01518 |
| LLAGPVAEYLK | 0.8798 | 1288.7105 | Q01518 |
| LLAGPVAEYLK | 0.9081 | 1288.7105 | Q01518 |
| LLAGPVAEYLK | 0.8815 | 1288.7105 | Q01518 |
| LLALEPELEAR | 0.8590 | 1340.7088 | P04843 |
| LLAYTLGVK | 0.8631 | 1092.6331 | P68104 |
| LLLAGVFR | 0.8367 | 975.5654 | Q9Y678 |
| LLLLVGGVDQSPR | 1.0000 | 1453.8041 | P33993 |
| LLSLAAAAK | 0.7301 | 972.5756 | P0CAP2-3 |
| LLSQNLVVKPDQLIK | 0.9543 | 1851.0982 | P53396 |
| LNGIPGLER | 0.8637 | 1055.5512 | Q8NH56 |
| LNSQKAGKE | 0.8945 | 1117.5880 | Q13185 |
| LNVVDIAGLVK | 0.9985 | 1255.7288 | Q9NTK5 |
| LQEYVANLLK | 0.9991 | 1305.7081 | O14980 |
| LQGIPVLVLGNK | 0.8000 | 1365.8132 | Q96BM9 |
| LQGIPVLVLGNKR | 0.9928 | 1521.9143 | Q96BM9 |
| LQGIPVLVLGNKR | 0.9952 | 1521.9143 | Q96BM9 |
| LQGIPVLVLGNKR | 0.9836 | 1521.9143 | Q96BM9 |
| LQGVDLLADAVAVTMGPK | 0.9998 | 1913.0080 | P10809 |
| LQGVDLLADAVAVTMGPK | 0.9998 | 1913.0006 | P10809 |
| LQLAIRNDE | 0.9724 | 1158.5781 | Q96QV6 |
| LQLAIRNDEE | 0.9876 | 1287.6207 | Q96QV6 |
| LQPLLDNQVGFK | 0.9129 | 1486.7932 | P20618 |
| LQTVAKNKDQGTYE | 0.9444 | 1737.8686 | P60660 |
| LSAGGAAVGGRR | 0.7299 | 1158.6006 | Q6ZSJ9 |
| LSASFEPFSNK | 0.9976 | 1341.6353 | P27797 |
| LSFMNTELAAFTK | 0.9941 | 1587.7677 | P31949 |
| LSLAEAQLR | 0.9913 | 1087.5774 | Q86UX7 |
| LSPLAAAVGGVASQEVLK | 0.9997 | 1825.0097 | A0AVT1 |
| LSVPCILGQNGISDLVK | 0.9992 | 1928.0189 | P00338 |
| LVADENPFAQGALK | 0.9999 | 1587.8045 | P06396 |

| **Identified Peptides (prime sequence)** | **PeptideProphet probability** | **Neutral peptide mass (Da)** | **Exemplary protein ID** |
| --- | --- | --- | --- |
| LVANVTNPNSTEHMK | 0.8630 | 1769.8519 | Q14974 |
| LVASNLNLKPGECLR | 0.9993 | 1798.9512 | P09382 |
| LVFDDVVGIVEIINSK | 0.9997 | 1875.0067 | Q01518 |
| LVFLPFADDKR | 0.9573 | 1435.7612 | P12956 |
| LVFLPFADDKR | 0.9727 | 1435.7612 | P12956 |
| LVFLPFADDKR | 0.9609 | 1435.7612 | P12956 |
| LVGAGAIGCELLK | 0.9757 | 1415.7595 | P22314 |
| LVGAGAIGCELLK | 0.9853 | 1415.752 | P31254 |
| LVGLFEDTNLCAIHAK | 0.9965 | 1915.9614 | P68431 |
| LVGLFEDTNLCAIHAK | 0.9983 | 1915.9614 | P68431 |
| LVGLIQK | 0.9638 | 885.5436 | P17655 |
| LVHWNTK | 0.8963 | 1012.5242 | P00918 |
| LVLTDPDAPSRK | 0.7857 | 1426.7568 | P30086 |
| LVSSSADPEGHFETPIWIER | 0.9745 | 2357.1076 | Q14697 |
| LVTASQCQQPAENK | 0.9306 | 1688.7940 | Q01518 |
| LVTASQCQQPAENK | 0.9619 | 1688.7940 | Q01518 |
| LVTASQCQQPAENK | 0.9994 | 1688.7866 | Q01518 |
| LVTASQCQQPAENK | 0.9998 | 1688.7867 | Q01518 |
| LVTGPLVLNR | 0.8597 | 1168.6716 | Q02878 |
| LVTYVPVTTFK | 0.7844 | 1382.7598 | P62899 |
| LVYQEPIPTAQLVQR | 0.9940 | 1841.9788 | P25787 |
| LYGLGELPQGFAR | 0.8151 | 1507.7571 | P31150 |
| LYYTGEKGQNQDYR | 0.9828 | 1849.8383 | P19338 |
| MADSGLLLK | 0.9534 | 1062.5531 | Q5SY16 |
| MANAGPNTNGSQFFICTAK | 0.9313 | 2143.9567 | A2BFH1 |
| MAPKPGPYVK | 0.9679 | 1230.6583 | Q01518 |
| MAPKPGPYVK | 0.8610 | 1230.6583 | Q01518 |
| MAPKPGPYVK | 0.9982 | 1230.6509 | Q01518 |
| MAPKPGPYVK | 0.9996 | 1230.6509 | Q01518 |
| MAPKPGPYVK | 0.9377 | 1230.6509 | Q01518 |
| MGNHELYMR | 0.9480 | 1237.5120 | P15311 |
| MIEIMEMK | 0.9947 | 1139.5177 | P40227 |
| MIEPIDEYCVQQLK | 0.9925 | 1880.88 | P07900 |
| MIGLPGAGK | 0.8389 | 958.4984 | Q00839 |
| MIVNNLLKPISVEGSSK | 0.9703 | 1972.0815 | Q9HB71 |
| MIVNNLLKPISVEGSSK | 0.9999 | 1972.0815 | Q9HB71 |
| MLARMASEVH | 0.7382 | 1247.5465 | B3QLW4 |
| MMQNPQILAALQER | 0.9998 | 1729.8391 | P55209 |
| MMTPTVLYDVQELR | 0.9925 | 1782.8433 | P09525 |

| **Identified Peptides (prime sequence)** | **PeptideProphet probability** | **Neutral peptide mass (Da)** | **Exemplary protein ID** |
| --- | --- | --- | --- |
| MPFVTEELFQR | 0.8886 | 1483.6918 | P26640 |
| MTEPIDEYCVQQLK | 0.9885 | 1868.8437 | Q58FF7 |
| MTEPIDEYCVQQLK | 0.9839 | 1868.8437 | Q58FF7 |
| MTEPIDEYCVQQLK | 0.9988 | 1868.8362 | Q58FF7 |
| MTSSYGHVLER | 0.8976 | 1382.6037 | P54821 |
| MTTVHAITATQK | 0.8629 | 1416.7183 | P04406 |
| MTTVHAITATQK | 0.9988 | 1416.7183 | P04406 |
| MVGSYGPRPEEYEFLTPVEEAPK | 0.9584 | 2740.2842 | P52566 |
| MVLTKMKE | 0.9640 | 1122.5929 | P11021 |
| MVPGKPMCVESFSDYPPLGR | 0.9496 | 2382.0959 | P68104 |
| MVPGKPMCVESFSDYPPLGR | 0.7906 | 2382.0959 | P68104 |
| MVPGKPMCVESFSDYPPLGR | 0.9647 | 2382.0959 | P68104 |
| MVTEALKPYSSGGPR | 0.9984 | 1707.8402 | P06744 |
| MVTPGHACTQK | 0.9750 | 1344.6067 | P04075 |
| MVTPGHACTQK | 0.8665 | 1344.5992 | P04075 |
| MVTPGHACTQK | 0.9986 | 1344.5992 | P04075 |
| MVTPGHACTQK | 0.9809 | 1344.5992 | P04075 |
| MVVTFAPVNVTTEVK | 0.8720 | 1749.9124 | P68104 |
| MVWEGLNVVK | 0.8713 | 1289.659 | O60361 |
| NAAGGLNPK | 0.9162 | 956.4828 | P00491 |
| NACFEPANQMVK | 0.9552 | 1523.6575 | P68362 |
| NACFEPANQMVK | 0.9969 | 1523.6575 | P68362 |
| NAHIQQVGDR | 0.9943 | 1224.5748 | Q00610 |
| NAHIQQVGDR | 0.9996 | 1224.5748 | Q00610 |
| NAPEQACHLAK | 0.9255 | 1353.6247 | P61981 |
| NAPPPELLEIINEDIAK | 0.7474 | 1991.0363 | Q15084 |
| NAPPPELLEIINEDIAKR | 1.0000 | 2147.1374 | Q15084 |
| NAPPPELLEIINEDIAKR | 0.9849 | 2147.1374 | Q15084 |
| NAQAAALYK | 0.9992 | 1064.5403 | P40121 |
| NAQAAALYK | 0.9072 | 1064.5329 | P40121 |
| NAQYLLQNSVK | 0.9995 | 1392.7149 | P61978 |
| NAQYLLQNSVK | 0.8884 | 1392.7075 | Q3T0D0 |
| NCDLHFK | 0.9971 | 1048.4548 | Q9BUJ2 |
| NCDLHFK | 0.9299 | 1048.4548 | Q9BUJ2 |
| NDGAAALVLMTADAAKR | 0.9577 | 1802.9097 | P24752 |
| NDGATILSMMDVDHQIAK | 0.9974 | 2073.9611 | P48643 |
| NDGATILSMMDVDHQIAK | 0.9989 | 2073.9611 | P48643 |
| NEASVLHNLK | 0.8660 | 1239.6360 | P35580 |
| NEASVLHNLK | 0.9489 | 1239.636 | P35580 |

| **Identified Peptides (prime sequence)** | **PeptideProphet probability** | **Neutral peptide mass (Da)** | **Exemplary protein ID** |
| --- | --- | --- | --- |
| NFIFGQTGAGNNWAK | 0.9538 | 1739.8168 | Q9BUF5 |
| NFTDGALVQHQEWDGK | 0.9966 | 1959.8864 | Q01469 |
| NFTDGALVQHQEWDGK | 0.9997 | 1959.8864 | Q01469 |
| NFVFGQSGAGNNWAK | 0.9960 | 1711.7855 | Q13885 |
| NHHLQETSFTK | 0.7314 | 1456.6847 | P13693 |
| NHPGQISAGYAPVLDCHTAHIACK | 0.9997 | 2732.2700 | P68104 |
| NHPGQISAGYAPVLDCHTAHIACK | 0.9915 | 2732.27 | P68104 |
| NIALLSDLTK | 0.9782 | 1202.6659 | P30048 |
| NIFISERPTDVLQTVK | 0.9922 | 1975.0527 | Q15813 |
| NIQLVTSQIDAQR | 0.9672 | 1572.7937 | Q86V81 |
| NLFVGNLNFNK | 0.9842 | 1394.7095 | P19338 |
| NLLKPISVEGSSK | 0.9767 | 1514.8456 | Q9HB71 |
| NLLKPISVEGSSK | 0.9933 | 1514.8387 | Q3T168 |
| NLNLKPGECLR | 0.9863 | 1428.7295 | P09382 |
| NLSYSATEETLQEVFEK | 0.9837 | 2102.9796 | P19338 |
| NLVVKPDQLIK | 0.9954 | 1409.8394 | P53396 |
| NLVVKPDQLIK | 0.9725 | 1409.8394 | P53396 |
| NMILDDGGDLTNLIHTK | 1.0000 | 1984.9676 | P23526 |
| NMLNPPAEVTTK | 0.8177 | 1429.6949 | P13010 |
| NNDIMLIK | 0.8238 | 1091.5433 | P07477 |
| NNDPLVLR | 0.8609 | 1027.5199 | Q9UH17 |
| NPGLAELIAEK | 0.9966 | 1269.6717 | P06737 |
| NPIISGLYQGAGGPGPGGFGAQGPK | 1.0000 | 2412.1975 | P08107 |
| NPIISGLYQGAGGPGPGGFGAQGPK | 0.9956 | 2412.1975 | P08107 |
| NPNIPNEIIHALQAGR | 0.9986 | 1843.9441 | P52272 |
| NPNTNDLFNAVGDGIVLCK | 0.9996 | 2176.0371 | P13796 |
| NPVDILTYVAWK | 0.9663 | 1533.7905 | P00338 |
| NSASAIGCHVVNIGAEDLK | 0.9999 | 2069.9952 | P13796 |
| NSFVNDIFER | 0.9173 | 1327.5945 | P33778 |
| NSFVNDIFER | 0.9997 | 1327.5945 | P33778 |
| NSFVNDIFER | 0.9754 | 1327.5945 | P33778 |
| NSFVNDIFER | 0.8381 | 1327.5871 | P06900 |
| NSFVNDIFER | 0.7422 | 1327.5871 | P06900 |
| NSPVWGADKCEELLEK | 0.9739 | 2017.9567 | O75367 |
| NSQLPVDHILAGSFETAMR | 0.9976 | 2173.0374 | P53621 |
| NTHADFADECPKPE | 0.9939 | 1745.7103 | P43487 |
| NTIDTLLSVVEDHK | 0.9999 | 1698.8577 | O95373 |
| NVDLSTVDKDQSIAPK | 0.9144 | 1872.9581 | P04844 |
| NVDLTEFQTNLVPYPR | 0.9993 | 1992.9617 | P68362 |

| **Identified Peptides (prime sequence)** | **PeptideProphet probability** | **Neutral peptide mass (Da)** | **Exemplary protein ID** |
| --- | --- | --- | --- |
| NVDLTEFQTNLVPYPR | 0.9992 | 1992.9617 | P68362 |
| NVGAGGPAPAAGAAPAGGPAPSTAAAPAEEK | 0.9985 | 2698.3099 | P05386 |
| NVLPVFDNLMQQK | 0.8634 | 1660.8395 | P07339 |
| NVNIGSLICNVGAGGPAPAAGAAPAGGPAPSTAAAPAEEK | 1.0000 | 3668.8005 | P05386 |
| NVSAVDKSTGKE | 0.9877 | 1377.6888 | P11142 |
| PDASKPEDWDER | 0.9999 | 1559.6567 | Q4VIT5 |
| PGLHVWR | 0.9807 | 951.4753 | P40121 |
| PLLSGLLDSPALK | 0.9210 | 1438.8109 | P49327 |
| PPAGSAPGEHVFVK | 0.9999 | 1507.7497 | P54577 |
| PPATQKAK | 0.8787 | 983.5552 | P50502 |
| PPAVAPR | 0.8262 | 794.4187 | Q9H9H4 |
| PTGTYHGDSDLQLDR | 0.9983 | 1761.7637 | Q9YHC3 |
| QAALKNPPINTK | 0.8806 | 1437.8092 | O15511 |
| QAAVYFEKGDYNK | 0.9943 | 1675.7994 | P31948 |
| QAELAVILK | 0.9447 | 1099.6389 | Q14980 |
| QALLELEMNSDLK | 0.9916 | 1618.8024 | P62081 |
| QALLELEMNSDLK | 0.9636 | 1618.795 | A6H769 |
| QANPILEAFGNAK | 0.9762 | 1487.7520 | P35749 |
| QARPDDLLISTYPK | 0.9508 | 1731.8944 | P50225 |
| QAYQEAFEISKK | 0.9994 | 1584.7936 | P31946 |
| QCQQPAENK | 0.9392 | 1217.5247 | Q01518 |
| QDAAIVGYK | 0.9882 | 1079.5399 | P07737 |
| QEAFEISKK | 0.9981 | 1222.6346 | P31946 |
| QEYDESGPSIVHR | 0.9416 | 1586.6677 | P53478 |
| QGESITHALK | 0.9999 | 1198.6094 | Q01518 |
| QGESITHALK | 0.9810 | 1198.6094 | Q01518 |
| QGESITHALK | 0.9998 | 1198.6094 | Q01518 |
| QGESITHALK | 0.9993 | 1198.6094 | Q01518 |
| QGESITHALK | 0.9877 | 1198.602 | Q01518 |
| QGESITHALK | 0.9987 | 1198.602 | Q01518 |
| QGESITHALK | 0.9963 | 1198.602 | Q01518 |
| QGESITHALK | 0.9982 | 1198.602 | Q01518 |
| QGESITHALK | 0.9891 | 1198.602 | Q01518 |
| QGESITHALK | 0.9831 | 1198.602 | Q01518 |
| QGESITHALK | 0.9943 | 1198.602 | Q01518 |
| QGESITHALK | 0.7683 | 1198.602 | Q01518 |
| QGESITHALK | 0.9301 | 1198.602 | Q01518 |
| QGESITHALK | 0.9684 | 1198.602 | Q01518 |
| QGGVLPNIQAVLLPK | 0.9772 | 1661.9617 | Q96QV6 |

| **Identified Peptides (prime sequence)** | **PeptideProphet probability** | **Neutral peptide mass (Da)** | **Exemplary protein ID** |
| --- | --- | --- | --- |
| QGGVLPNIQAVLLPK | 0.9948 | 1661.9617 | Q96QV6 |
| QGGVLPNIQAVLLPK | 0.9384 | 1661.9542 | P04908 |
| QGTVIHFNNPK | 0.9952 | 1369.6891 | Q13892 |
| QHGKVEIIANDQGNR | 0.8943 | 1793.8921 | P34931 |
| QHLGESTVR | 0.9486 | 1113.5315 | P06576 |
| QHNDIIR | 0.9882 | 982.4733 | Q14697 |
| QLGNIVFK | 0.9433 | 1033.5709 | P35749 |
| QLLLENLGNENVHR | 0.7279 | 1735.8753 | Q14974 |
| QLLLFASK | 0.9758 | 1034.5912 | Q08211 |
| QLTHSLGGGTGSGMGTLLISK | 0.9951 | 2130.0892 | Q13885 |
| QLTHSLGGGTGSGMGTLLISK | 0.9508 | 2130.0892 | Q13885 |
| QPILLELEAPLK | 0.9997 | 1478.8496 | P62136 |
| QPILLELEAPLK | 0.9976 | 1478.8427 | Q61JR3 |
| QPLVILEMESGASAK | 1.0000 | 1687.8603 | P49588 |
| QPPAAPPAAPALSAADTKPGTTGSGAGSGGPGGLTSAAPAGGDKK | 0.9670 | 4039.0399 | P67809 |
| QPSFLGMESCGIHETTFNSIMK | 1.0000 | 2629.1763 | P60709 |
| QPSFLGMESCGIHETTFNSIMK | 0.9992 | 2629.1763 | P60709 |
| QPTVGMNFKTPRGPV | 0.9243 | 1743.8879 | P08708 |
| QQLDLTHLK | 0.9518 | 1210.6458 | P61221 |
| QQNGIVPIVEPEILPDGDHDLK | 0.9951 | 2541.2863 | P04075 |
| QSEVKPILEK | 0.9773 | 1313.7343 | P30153 |
| QSPVDIDTHTAK | 0.9999 | 1409.6501 | P00918 |
| QVAEVFTGHMGK | 1.0000 | 1418.6765 | P06576 |
| QVAEVFTGHMGK | 0.8758 | 1418.669 | Q5ZLC5 |
| QYLLTLGFK | 0.8629 | 1197.6546 | Q9BXB7 |
| QYLLTLGFK | 0.8742 | 1197.6546 | Q9BXB7 |
| QYLLTLGFK | 0.8936 | 1197.6546 | Q9BXB7 |
| RDQNILLGTTYR | 0.9965 | 1536.7797 | P78527 |
| RDTKENGKHMDL | 0.9712 | 1586.7623 | Q86XP1-3 |
| RDTKENGKHMDL | 0.8933 | 1586.7623 | Q86XP1-3 |
| REPIICK | 0.7282 | 1030.5382 | P48735 |
| REPVVTLEGHTK | 0.9857 | 1480.7786 | P31146 |
| RFDQLFDDESDPFEVLK | 0.9879 | 2215.0222 | Q8NC51 |
| RIVAPGKGILAADE | 0.9780 | 1524.8412 | P04075 |
| RLPLQDVYK | 0.9964 | 1246.6822 | P68104 |
| RLPLQDVYK | 0.9563 | 1246.6822 | P68104 |
| RLPLQDVYK | 0.7985 | 1246.6748 | P68103 |
| RNPLIAGK | 0.9675 | 983.5664 | P62316 |
| RPDNFVFGQSGAGNNWAK | 0.9997 | 2079.9663 | Q13885 |

| **Identified Peptides (prime sequence)** | **PeptideProphet probability** | **Neutral peptide mass (Da)** | **Exemplary protein ID** |
| --- | --- | --- | --- |
| RPDNFVFGQSGAGNNWAK | 0.9995 | 2079.9663 | Q13885 |
| RPGLEGYALPR | 0.9196 | 1315.6785 | P33992 |
| RPGLVVVHAEDGTTSK | 0.9913 | 1780.9220 | P30049 |
| RPQNYLFGCELK | 0.9561 | 1639.7929 | P06748 |
| RPQNYLFGCELK | 0.9839 | 1639.7929 | P06748 |
| RPQNYLFGCELK | 0.9942 | 1639.7857 | Q3T160 |
| RSDSENILTNYENQSR | 0.9119 | 2012.8936 | Q03001 |
| RTVSLGAGAKDE | 0.9504 | 1318.6629 | P06748 |
| RTVSLGAGAKDE | 0.9768 | 1318.6629 | P06748 |
| RVHIPNDDAQFD | 0.9955 | 1513.6698 | Q16576 |
| RVPAGNWVLIEGVDQPIVK | 0.9954 | 2205.2058 | Q15029 |
| SAAAVLSHNR | 1.0000 | 1112.5475 | P04844 |
| SAALIQQATTVK | 0.9947 | 1345.7354 | P32969 |
| SAALIQQATTVK | 0.9753 | 1345.7354 | P32969 |
| SADFPALVVK | 0.8513 | 1161.6182 | P22102 |
| SADPEGHFETPIWIER | 0.9965 | 1970.8911 | Q14697 |
| SAGIMDHEEAR | 0.9771 | 1302.5411 | P62244 |
| SAGIMDHEEAR | 0.9961 | 1302.5411 | P62244 |
| SAGTQCLISGWGNTK | 0.9998 | 1694.776 | P00760 |
| SAGTQCLISGWGNTK | 0.9999 | 1694.7757 | P00760 |
| SAGTQCLISGWGNTK | 0.9999 | 1694.7757 | P00760 |
| SAGTQCLISGWGNTK | 0.9938 | 1694.7757 | P00760 |
| SALAAATAAAAAAASAAAATAA | 0.8837 | 1802.8837 | Q99932 |
| SALGIPSLLPFLK | 0.9303 | 1470.8598 | O75533 |
| SALGIPSLLPFLK | 0.9911 | 1470.8527 | O75533 |
| SALILHDDE | 0.8397 | 1099.4934 | P05386 |
| SAPAFSLVFPFLK | 0.9691 | 1538.8211 | Q92616 |
| SAPGPLELDLTGDLESFKK | 0.9879 | 2160.1102 | P52565 |
| SAPKPQTSPSPK | 0.9626 | 1367.7197 | Q01518 |
| SAPKPQTSPSPK | 0.9833 | 1367.7197 | Q01518 |
| SAPVLAVAGLGDSNQFFR | 0.9997 | 1935.9591 | Q9UHL4 |
| SASAIGCHVVNIGAEDLK | 0.9018 | 1955.9523 | P13796 |
| SASAIGCHVVNIGAEDLK | 0.9997 | 1955.9523 | P13796 |
| SASAIGCHVVNIGAEDLK | 0.9998 | 1955.9523 | P13796 |
| SASIPGILALDLCPSDTNK | 0.9949 | 2087.0357 | Q9UMS4 |
| SASIPGILALDLCPSDTNK | 0.9226 | 2087.0357 | Q9UMS4 |
| SASTPVFGGILSLINEHR | 0.9581 | 1985.0119 | O14773 |
| SATMPSDVLEVTK | 0.7211 | 1492.7231 | P60842 |
| SATMPSDVLEVTKK | 0.9995 | 1648.8494 | P60842 |

| **Identified Peptides (prime sequence)** | **PeptideProphet probability** | **Neutral peptide mass (Da)** | **Exemplary protein ID** |
| --- | --- | --- | --- |
| SATMPSDVLEVTKK | 0.8368 | 1648.8494 | P60842 |
| SCGLTHTAVVPLDLVK | 0.9998 | 1824.9556 | Q00325-2 |
| SCVGVFQHGK | 0.9991 | 1233.5713 | P34931 |
| SCVGVFQHGK | 0.9645 | 1233.5713 | P34931 |
| SDNAPPPELLEIINEDIAKR | 0.9927 | 2349.1964 | Q15084 |
| SDYPPLGR | 0.9982 | 991.4511 | P68104 |
| SDYPPLGR | 0.9501 | 991.4437 | P68103 |
| SEAYLVGLFEDTNLCAIHAK | 1.0000 | 2366.1365 | Q71DI3 |
| SEIAALLVKPQK | 0.9563 | 1439.8500 | Q8NF50 |
| SENFQTLLDAGLPQK | 0.9984 | 1775.8767 | O60506 |
| SENFQTLLDAGLPQK | 0.9993 | 1775.8767 | O60506 |
| SEVILPVPAFNVINGGSHAGNK | 0.7623 | 2335.2073 | P06733 |
| SFNPYSEFILATGSADK | 0.8721 | 1961.9159 | Q09028 |
| SFVDKDLLEPGCSVLLNHK | 0.9514 | 2314.1780 | P62191 |
| SGGPVVCSGK | 0.9481 | 1062.4842 | P00760 |
| SGGPVVCSGK | 0.9979 | 1062.4842 | P00760 |
| SGGPVVCSGK | 0.9972 | 1062.4842 | P00760 |
| SGGQHTVLLVK | 0.9660 | 1253.6880 | P18754 |
| SGGTTMYPGIADR | 0.9826 | 1428.6092 | P62736 |
| SGGTTMYPGIADR | 0.9989 | 1428.6092 | P62736 |
| SGGTTMYPGIADR | 0.9987 | 1428.6018 | P53478 |
| SGGTTMYPGIADR | 0.9946 | 1428.6018 | P53478 |
| SGGTTMYPGIADR | 0.7323 | 1428.6018 | P53478 |
| SGGTTMYPGIADR | 0.9802 | 1428.6018 | P53478 |
| SGGTTMYPGIADR | 0.7711 | 1428.6018 | P53478 |
| SGGTTMYPGIADR | 0.9982 | 1428.6018 | P53478 |
| SGGTTMYPGIADR | 0.9987 | 1412.6143 | P62736 |
| SGGTTMYPGIADR | 0.9030 | 1412.6143 | P62736 |
| SGGTTMYPGIADR | 0.9991 | 1412.6068 | P53478 |
| SGGTTMYPGIADR | 0.9992 | 1412.6068 | P53478 |
| SGGTTMYPGIADR | 0.9992 | 1412.6068 | P53478 |
| SGGTTMYPGIADR | 0.9990 | 1412.6068 | P53478 |
| SGGTTMYPGIADR | 0.9888 | 1412.6068 | P53478 |
| SGGTTMYPGIADR | 0.9345 | 1412.6068 | P53478 |
| SGGTTMYPGIADR | 0.9773 | 1412.6068 | P53478 |
| SGGTTMYPGIADR | 0.9633 | 1412.6068 | P53478 |
| SGGTTMYPGIADRMQKE | 0.8656 | 1956.8822 | P62736 |
| SGGVLPNIHPELLAK | 0.9742 | 1659.9096 | O75367 |
| SGGVLPNIHPELLAK | 0.9738 | 1659.9096 | O75367 |

| **Identified Peptides (prime sequence)** | **PeptideProphet probability** | **Neutral peptide mass (Da)** | **Exemplary protein ID** |
| --- | --- | --- | --- |
| SGIPAGWMGLDCGPESSKK | 0.9998 | 2119.9819 | P00558 |
| SGIPAGWMGLDCGPESSKK | 0.9960 | 2119.9819 | P00558 |
| SGSPFPGSVQDPGLHVWR | 0.9994 | 2009.9496 | P40121 |
| SGSPFPGSVQDPGLHVWR | 0.9998 | 2009.9496 | P40121 |
| SGSPFPGSVQDPGLHVWR | 0.9989 | 2009.9417 | P40121 |
| SGSSHQDLSQR | 0.9998 | 1288.5544 | P11908 |
| SGSSHQDLSQR | 0.9719 | 1288.5544 | P11908 |
| SGVTTCLR | 0.9598 | 980.4498 | Q13885 |
| SGVTTCLR | 0.7225 | 980.4498 | Q13885 |
| SGVTTCLR | 0.8879 | 980.4498 | Q13885 |
| SHDASTNGLINFIK | 0.9948 | 1631.8056 | P06744 |
| SHDGAFLAVCDASK | 1.0000 | 1592.7041 | O75083 |
| SHPLIPDK | 0.9203 | 1021.5345 | Q9NSE4 |
| SHTLAVDAK | 0.9560 | 1056.5352 | Q14289 |
| SISIALIGGSR | 0.9414 | 1160.6301 | Q86UK0 |
| SIVPALEIANAHR | 0.9475 | 1477.7789 | P10809 |
| SIVPALEIANAHR | 0.9964 | 1477.7717 | P10809 |
| SIVPALEIANAHR | 0.9713 | 1477.7717 | P10809 |
| SLAGGIIGVK | 0.9877 | 1029.5971 | P61978 |
| SLDIQCEELSDAR | 0.9595 | 1622.692 | P13489 |
| SLFLTDLYSPEYPGPSHR | 0.9993 | 2166.0170 | Q16181 |
| SLFLTDLYSPEYPGPSHR | 0.9982 | 2166.017 | Q16181 |
| SLGGGTGSGMGTLLISK | 0.9150 | 1650.8327 | Q9YHC3 |
| SLGGGTGSGMGTLLISK | 0.9758 | 1650.8327 | Q9YHC3 |
| SLIALVNDPQPEHPLR | 0.9995 | 1885.9798 | P68036 |
| SLIALVNDPQPEHPLR | 0.9947 | 1885.9798 | P68036 |
| SLLAGPVAEYLK | 0.8111 | 1375.7499 | Q01518 |
| SLLAGPVAEYLK | 0.9676 | 1375.7425 | Q01518 |
| SLLAGPVAEYLK | 0.8999 | 1375.7425 | Q01518 |
| SLLDKFLIK | 0.9230 | 1219.7328 | Q04917 |
| SLLDKFLIK | 0.7952 | 1219.7328 | Q04917 |
| SLLDKFLIK | 0.9389 | 1219.7328 | Q04917 |
| SLPLDTLLVDVEPK | 0.9969 | 1653.8977 | P62314 |
| SLPLDTLLVDVEPK | 0.9666 | 1653.8977 | P62314 |
| SLPLDTLLVDVEPK | 0.9398 | 1653.8977 | P62314 |
| SLPLITASILSK | 0.9997 | 1357.7969 | P19971 |
| SLVDLKAELFR | 0.9095 | 1405.7717 | Q6PII3 |
| SMANAGPNTNGSQFFICTAK | 0.9998 | 2230.9888 | A2BFH1 |
| SMMDVDHQIAK | 0.9988 | 1389.6169 | P48643 |

| **Identified Peptides (prime sequence)** | **PeptideProphet probability** | **Neutral peptide mass (Da)** | **Exemplary protein ID** |
| --- | --- | --- | --- |
| SMMGQLMKPK | 0.9698 | 1293.6395 | Q9Y265 |
| SNLLDLNPQNINK | 0.9925 | 1597.8212 | P63010 |
| SNLNLKPGECLR | 0.9998 | 1515.7616 | P09382 |
| SNNLCLHFNPR | 0.9874 | 1458.65 | P09382 |
| SNPLLEAFGNAK | 0.7821 | 1375.6884 | Q12965 |
| SNQGGLVHPK | 0.9914 | 1151.5836 | P56537 |
| SNVLIIGELLK | 0.8793 | 1313.7707 | Q92526 |
| SNVLIIGELLK | 0.8850 | 1313.7707 | Q92526 |
| SPAPAAAAPAVQ | 0.7793 | 1137.5492 | P51610 |
| SPAVHLDLLSLR | 1.0000 | 1407.7622 | P34810 |
| SPPVAMETASTGVAAVP | 0.8109 | 1671.7852 | Q64548 |
| SPSSSIVPAFNTGTITQVIK | 0.9021 | 2162.1371 | O43747 |
| SQCGSLIGK | 0.9208 | 1064.5073 | Q15366 |
| SQCQQPAENK | 0.9994 | 1304.5567 | Q01518 |
| SQCQQPAENK | 0.999 | 1304.5567 | Q01518 |
| SQCQQPAENK | 0.9286 | 1304.5567 | Q01518 |
| SQEESIKPK | 0.8264 | 1188.6138 | Q9UBQ5 |
| SQHQALLGTIR | 0.9995 | 1310.6843 | P25705 |
| SQLLNGLK | 0.9120 | 987.5501 | Q8NCG7 |
| SQLSAAVTALNSESNFAR | 1.0000 | 1952.9340 | O75390 |
| SQLSAAVTALNSESNFAR | 0.9513 | 1952.9340 | O75390 |
| SQQAYQEAFEISKK | 0.9999 | 1799.8842 | P31946 |
| SQVLAGLMEAQK | 0.9989 | 1389.7074 | Q96EK9 |
| SQVTTVCQALAK | 0.9295 | 1420.7133 | P50897 |
| SSAPGPLELDLTGDLESFKK | 0.9999 | 2247.1423 | P52565 |
| SSEGVPDLLV | 0.8156 | 1102.522 | Q0Q473 |
| SSEPACLAEIEEDKAR | 0.9771 | 1919.8683 | P78527 |
| SSFGPISEVVVVK | 0.9673 | 1462.7820 | P98179 |
| SSGFSLEDPQTHSNR | 0.8627 | 1748.7502 | P08238 |
| SSGGLSKDDIENMVK | 0.9998 | 1722.8246 | P38646 |
| SSGGLSKDDIENMVK | 0.9965 | 1722.8246 | P38646 |
| SSHIANVER | 0.9134 | 1099.5158 | P06396 |
| SSIVPAFNTGTITQVIK | 0.7206 | 1891.0203 | O43747 |
| SSLAATLLANHSLR | 0.9999 | 1540.8109 | P13489 |
| SSLAATLLANHSLR | 0.9998 | 1540.8109 | P13489 |
| SSLAATLLANHSLR | 0.9980 | 1540.8037 | P13489 |
| SSLMGLFEK | 0.9992 | 1126.5481 | P50395 |
| SSMAEVDAAMAARPHSIDGR | 0.9721 | 2158.9636 | P22626 |
| SSMLREQILDLSK | 0.9145 | 1650.8399 | Q09328 |

| **Identified Peptides (prime sequence)** | **PeptideProphet probability** | **Neutral peptide mass (Da)** | **Exemplary protein ID** |
| --- | --- | --- | --- |
| SSSQELGAALAQLVAQR | 0.9946 | 1815.9157 | O95336 |
| SSTFDAGAGIALNDHFVK | 0.9856 | 1964.9380 | P04406 |
| STAIQELFK | 0.9355 | 1151.5975 | Q13885 |
| STAIQELFK | 0.7894 | 1151.59 | Q9YHC3 |
| STAIQELFKR | 0.9821 | 1307.6986 | Q13885 |
| STAIQELFKR | 0.9231 | 1307.6986 | Q13885 |
| STDLPLNIECFMNDKDVSGK | 0.8491 | 2426.1246 | Q92598 |
| STSHVPEVDPGSAELQK | 0.9750 | 1895.9013 | P49327 |
| STSLLGPPPGLLTPPVATELSQNAR | 0.9982 | 2603.3707 | Q8N163 |
| STVFKDDDDVVIGK | 0.9879 | 1680.8359 | O15144 |
| SVEQITAMLLTK | 0.9024 | 1448.7697 | Q92598 |
| SVEVDGNSFEASGPSKK | 1.0000 | 1880.8904 | Q12906 |
| SVEVDGNSFEASGPSKK | 0.9927 | 1880.8904 | Q12906 |
| SVGIDHLALDEIK | 0.9997 | 1524.7936 | Q9UBQ7 |
| SVHYPGEAVATR | 0.8563 | 1373.6397 | O94808 |
| SVLNLVIVK | 0.8213 | 1099.6753 | P62753 |
| SVLNVLHSLVDK | 0.9997 | 1438.7932 | Q9Y262 |
| SVLTQSVK | 0.9674 | 976.5341 | P62316 |
| SVPAVPGALGPLTITSSAVTGR | 0.9408 | 2138.1484 | O95758 |
| SVSLVADENPFAQGALK | 0.9999 | 1860.9370 | P06396 |
| SVSLVADENPFAQGALK | 0.9945 | 1860.9297 | P06396 |
| SVSLVADENPFAQGALK | 0.9450 | 1860.9297 | P06396 |
| SVSLVADENPFAQGALR | 0.9914 | 1860.9044 | P13020 |
| SVTLHQDQLK | 0.7908 | 1283.6622 | P53602 |
| SVVIIAAELLK | 0.9977 | 1270.7648 | P17987 |
| SYLGGFDSSSNVLAGQLR | 0.8691 | 1957.9282 | Q6XQN6 |
| SYPLSEGQLDQK | 0.9229 | 1479.6919 | P23141 |
| SYVGDEAQSK | 0.8796 | 1198.518 | P53478 |
| SYVGDEAQSK | 0.9994 | 1198.518 | P53478 |
| SYVGDEAQSK | 0.9993 | 1198.518 | P53478 |
| SYVGDEAQSKR | 0.9980 | 1354.6191 | P53478 |
| SYVGDEAQSKR | 0.9995 | 1354.6191 | P53478 |
| SYVGDEAQSKR | 0.9967 | 1354.6191 | P53478 |
| SYVGDEAQSKR | 0.8097 | 1354.6191 | P53478 |
| SYVGDEAQSKR | 0.9919 | 1354.6191 | P53478 |
| SYVGDEAQSKR | 0.9607 | 1354.6191 | P53478 |
| TAASSSSLEK | 0.9267 | 1095.5122 | P53478 |
| TAASSSSLEK | 0.8598 | 1095.5122 | P53478 |
| TAEILELAGNAAR | 0.9973 | 1415.7156 | P04908 |

| **Identified Peptides (prime sequence)** | **PeptideProphet probability** | **Neutral peptide mass (Da)** | **Exemplary protein ID** |
| --- | --- | --- | --- |
| TAHIACK | 0.9615 | 915.4385 | P68104 |
| TAHIACK | 0.9343 | 915.4385 | P68104 |
| TALLQPHDR | 0.8171 | 1137.5679 | P34897 |
| TAPVNIAVIK | 0.7510 | 1140.6655 | P53602 |
| TAQLDEELGGTPVQSR | 0.9848 | 1787.8367 | P06396 |
| TDINLPYLTMDSSGPK | 0.9244 | 1866.8822 | P38646 |
| TDINLPYLTMDSSGPK | 0.9963 | 1866.8747 | P38646 |
| TDINLPYLTMDSSGPK | 0.9865 | 1866.8747 | P38646 |
| TGLAWSKTGPVAKE | 0.9973 | 1587.8409 | Q01518 |
| TGNVPLKVGQK | 0.9258 | 1283.7350 | Q8WUE5 |
| THGLNEEQR | 0.8291 | 1170.5166 | O95373 |
| THNMDVPNIK | 0.9235 | 1283.6080 | P63241 |
| THSLGGGTGSGMGTLLISK | 0.9465 | 1904.9414 | Q13885 |
| THSLGGGTGSGMGTLLISK | 0.8868 | 1904.9414 | Q13885 |
| THSLGGGTGSGMGTLLISK | 0.9927 | 1888.9465 | Q13885 |
| THSLGGGTGSGMGTLLISK | 0.9678 | 1888.9465 | Q13885 |
| THSLGGGTGSGMGTLLISK | 0.8534 | 1888.9465 | Q13885 |
| THSLGGGTGSGMGTLLISK | 0.9999 | 1888.9387 | Q9YHC3 |
| THSLGGGTGSGMGTLLISK | 0.9999 | 1888.9387 | Q9YHC3 |
| THSLGGGTGSGMGTLLISK | 0.9999 | 1888.9387 | Q9YHC3 |
| THSLGGGTGSGMGTLLISK | 0.7353 | 1888.9387 | Q9YHC3 |
| TISANGDKEIGNIISDAMKK | 0.9180 | 2276.1834 | P10809 |
| TLAVNAAQDSTDLVAK | 0.9947 | 1731.8717 | Q32L40 |
| TLAVNAAQDSTDLVAK | 0.9925 | 1731.8717 | Q32L40 |
| TLHLLPCEVAVDGPAPVGR | 0.9976 | 2088.0574 | Q8TDP1 |
| TLPAGPEIGPSPAPPYGLFVGGR | 0.9532 | 2337.1906 | Q8IZ83 |
| TLPHEILEMTNK | 0.9962 | 1540.7707 | P38919 |
| TMSGVTTCLR | 0.9926 | 1212.5379 | Q13885 |
| TNDFLSLLEK | 0.9997 | 1294.6557 | O14929 |
| TNLCAIHAK | 0.9961 | 1142.558 | P84227 |
| TNLCAIHAK | 0.9897 | 1142.558 | P84227 |
| TPAPVEKSPAK | 0.9137 | 1267.6924 | P16401 |
| TPEIMAPILANADVQER | 1.0000 | 1954.9570 | Q16186 |
| TPEIMAPILANADVQER | 0.9976 | 1954.9570 | Q16186 |
| TPGVAADLSHIETK | 0.9045 | 1553.7763 | P40926 |
| TPGVAADLSHIETK | 0.9894 | 1553.7767 | P40926 |
| TPIEGMLSHQLK | 0.9997 | 1468.7496 | Q9UQ80 |
| TPLLDYALEVEK | 1.0000 | 1505.7765 | P53396 |
| TPLLPSTTGLLNDNTFAQCK | 0.9999 | 2306.1365 | O43175 |

| **Identified Peptides (prime sequence)** | **PeptideProphet probability** | **Neutral peptide mass (Da)** | **Exemplary protein ID** |
| --- | --- | --- | --- |
| TPLLPSTTGLLNDNTFAQCK | 0.9383 | 2306.1365 | O43175 |
| TPRPVIVEPLEQLDDEDGLPEK | 0.9863 | 2604.3071 | P23246 |
| TQCGSLIGK | 0.8146 | 1078.5229 | Q15365 |
| TQPPPAPAPHATLPR | 0.9838 | 1637.8426 | P49327 |
| TQQLHAAMADTFLEHMCR | 0.8911 | 2246.9771 | P14618 |
| TSAGIMDHEEAR | 0.9999 | 1403.5887 | P62244 |
| TSEGVCLAVEK | 0.9896 | 1307.6179 | P28066 |
| TSELDMSESKTR | 0.9268 | 1498.6721 | Q86X24 |
| TSILEYPIEPSGVLGAVATK | 0.9762 | 2160.1466 | Q96PU8 |
| TTAIAEAWAR | 0.8833 | 1176.5675 | Q71U36 |
| TTHELTIPNNLIGCIIGR | 1.0000 | 2109.0789 | Q15365 |
| TTNCLAPLAK | 0.9794 | 1203.6070 | O14556 |
| TTSAGIMDHEEAR | 0.9998 | 1504.6364 | P62244 |
| TTSAGIMDHEEAR | 0.9990 | 1504.6364 | P62244 |
| TTSAGIMDHEEAR | 0.9997 | 1504.6364 | P62244 |
| TTSAGIMDHEEAR | 0.9975 | 1504.629 | Q9LX88 |
| TTSHELTIPNDLIGCIIGR | 0.9981 | 2197.0949 | Q15366 |
| TTVHAITATQK | 0.9965 | 1285.6779 | P04406 |
| TTVHAITATQK | 0.9963 | 1285.6779 | P04406 |
| TTVHAITATQK | 0.8932 | 1285.6779 | P04406 |
| TVEGPPPKDTGIAR | 0.9959 | 1552.7998 | P14678 |
| TVEGPPPKDTGIAR | 0.8886 | 1552.7998 | P14678 |
| TVFTPLEYGACGLSEEK | 0.9876 | 2015.9298 | Q16881 |
| TVLSGGTTMYPGIADR | 0.9868 | 1741.8093 | P60709 |
| TVLSGGTTMYPGIADR | 0.9975 | 1725.8067 | P53478 |
| TVLSGGTTMYPGIADR | 0.9984 | 1725.8067 | P53478 |
| TVLSGGTTMYPGIADR | 0.9917 | 1725.8067 | P53478 |
| TVPIYEGYALPHAILR | 0.9212 | 1899.9995 | P60709 |
| TVPIYEGYALPHAILR | 0.9998 | 1899.9995 | P60709 |
| TVPIYEGYALPHAILR | 0.9969 | 1899.9995 | P60709 |
| TVPIYEGYALPHAILR | 0.9976 | 1899.9917 | P53478 |
| TYGWTANMER | 0.8779 | 1315.5403 | Q58FG1 |
| VADLAESIMK | 0.9952 | 1191.5957 | P00338 |
| VADLQLIDFEGKK | 0.9945 | 1618.8719 | Q9Y376 |
| VAEITNACFEPANQMVK | 0.9237 | 2036.9448 | Q71U36 |
| VAEITNACFEPANQMVK | 0.8759 | 2036.9448 | Q71U36 |
| VAGLVAHSDLDER | 0.9344 | 1468.7058 | O60506 |
| VAGLVAHSDLDER | 0.9984 | 1468.6984 | O60506 |
| VAIVDPHIKVD | 0.9817 | 1320.7190 | Q14697 |

| **Identified Peptides (prime sequence)** | **PeptideProphet probability** | **Neutral peptide mass (Da)** | **Exemplary protein ID** |
| --- | --- | --- | --- |
| VALAGLLAAQK | 0.9524 | 1169.692 | P23368 |
| VALDFEQEMATAASSSSLEK | 0.9998 | 2229.0259 | P60709 |
| VAPPGARQGQQQAGGDGKTE | 0.9937 | 2066.9882 | Q00839-2 |
| VASNLNLKPGECLR | 0.9986 | 1685.8671 | P09382 |
| VASNLNLKPGECLR | 0.7846 | 1685.8597 | P09382 |
| VEQNFPAIAIHR | 0.9225 | 1481.7527 | O00148 |
| VFFDIAVDGEPLGR | 0.9987 | 1621.7814 | Q6DTV9 |
| VFGPDKK | 0.9165 | 933.5072 | P30041 |
| VGLFEDTNLCAIHAK | 1.0000 | 1802.8773 | P68431 |
| VGMGQKDSYVGDEAQSKR | 0.9982 | 2097.9902 | P62736 |
| VHAITATQK | 0.9936 | 1083.5825 | P04406 |
| VHAITATQK | 0.9510 | 1083.5825 | P04406 |
| VHAITATQK | 0.9848 | 1083.5751 | P10096 |
| VHAITATQK | 0.9376 | 1083.5751 | P10096 |
| VHAITATQK | 0.9255 | 1083.5751 | P10096 |
| VHAITATQK | 0.9617 | 1083.5751 | P10096 |
| VHGALAPLAIPSAAAAAAAAGR | 0.9954 | 2014.0860 | P26599 |
| VHGALAPLAIPSAAAAAAAAGR | 0.9237 | 2014.0860 | P26599 |
| VHGALAPLAIPSAAAAAAAAGR | 0.9967 | 2014.086 | P26599 |
| VHLDLLSLR | 0.9999 | 1152.6403 | P34810 |
| VHLDLLSLR | 0.9197 | 1152.6403 | P34810 |
| VIANPVNSTIPITAEVFKK | 1.0000 | 2184.2306 | P40926 |
| VIPAAHPVGT | 0.9226 | 1048.538 | B1KHV0 |
| VLAAELLR | 0.9773 | 971.5552 | P78371 |
| VLAKPTPK | 0.8963 | 996.6120 | Q92954 |
| VLIPTEGGDFNEFPVPEQFK | 0.9999 | 2378.1583 | Q01518 |
| VLLGPPGAGKGTQAPRLAE | 0.9995 | 1947.0690 | P54819 |
| VLPHILDTGAAGR | 0.8570 | 1406.7418 | Q15084 |
| VLQPGTALFS | 0.7304 | 1119.5638 | P47224 |
| VMGLLSNNNQALR | 0.9868 | 1516.7568 | Q13283 |
| VMVGMGQK | 0.9478 | 964.4622 | P62736 |
| VMVGMGQKDSYVGDEAQSK | 0.9989 | 2171.9980 | P62736 |
| VMVGMGQKDSYVGDEAQSKR | 0.9801 | 2328.0991 | P62736 |
| VNHPQVSALLGEEDEEALHYLTR | 0.9998 | 2707.2990 | Q01105 |
| VNITPAEVGVLVGK | 0.9984 | 1510.8507 | P07737 |
| VNITPAEVGVLVGKDR | 0.9146 | 1781.9788 | P07737 |
| VPAPLPKKISSE | 0.9097 | 1408.8078 | P14317 |
| VPGKPMCVESFSDYPPLGR | 0.9989 | 2251.0554 | P68104 |
| VPITFQVK | 0.9373 | 1046.5913 | P05107 |

| **Identified Peptides (prime sequence)** | **PeptideProphet probability** | **Neutral peptide mass (Da)** | **Exemplary protein ID** |
| --- | --- | --- | --- |
| VQAFDSLLAGPVAEYLK | 0.9991 | 1936.0017 | Q01518 |
| VQAFDSLLAGPVAEYLK | 0.9969 | 1936.0017 | Q01518 |
| VQAFQFTDKHGE | 0.9683 | 1521.7001 | Q06830 |
| VQEISHLIEPLANAAR | 0.9999 | 1847.9641 | Q9Y490 |
| VQGLGENVTIESVADYFK | 0.9977 | 2084.0214 | P35637 |
| VQSGMVVGLGTGSTTAFV | 0.9204 | 1797.8647 | Q8DJF2 |
| VQSGSHLAAR | 0.9763 | 1112.5475 | P04040 |
| VQSGSHLAAR | 0.9057 | 1112.5475 | P04040 |
| VQSGSHLAAR | 0.9966 | 1112.5475 | P04040 |
| VSAVDKSTGKE | 0.9698 | 1263.6459 | P11142 |
| VSLGGFEITPPVVLR | 0.9934 | 1670.9144 | P06748 |
| VSLGGFEITPPVVLR | 0.9968 | 1670.9067 | Q3T160 |
| VSLINLAMK | 0.9917 | 1103.6161 | Q96QK1 |
| VSNLVIEDTELK | 0.9987 | 1474.7667 | Q01518 |
| VSSFYHAFSGAQK | 0.8008 | 1543.7208 | P12814 |
| VSVLQLFCSSPK | 0.9260 | 1479.7544 | Q9Y678 |
| VTASQCQQPAENK | 1.0000 | 1575.7099 | Q01518 |
| VTASQCQQPAENK | 0.9983 | 1575.7025 | Q01518 |
| VTASQCQQPAENK | 0.9999 | 1575.7025 | Q01518 |
| VTASQCQQPAENK | 0.9991 | 1575.7025 | Q01518 |
| VTASQCQQPAENK | 0.9202 | 1575.7025 | Q01518 |
| VTASQCQQPAENK | 0.9999 | 1575.7025 | Q01518 |
| VTASQCQQPAENK | 0.9999 | 1575.7025 | Q01518 |
| VTKYTSAK | 0.9859 | 1040.5654 | P06899 |
| VTKYTSSK | 0.8905 | 1056.5604 | Q96A08 |
| VVAVLPHILDTGAAGR | 0.9999 | 1675.9158 | Q15084 |
| VVAVLPHILDTGAAGR | 0.9998 | 1675.9158 | Q15084 |
| VVSAAHCYK | 0.9567 | 1149.5389 | P35030 |
| VVSAAHCYK | 0.9961 | 1149.5389 | P35030 |
| VWNTHADFADECPKPELLAIR | 0.7899 | 2597.2485 | P43487 |
| WDMLDLAK | 0.9126 | 1106.5218 | P49321 |
| WIGENVSGLQR | 0.9761 | 1345.6527 | Q14019 |
| WQGLIVPDNPPYDK | 0.9828 | 1756.8573 | P68036 |
| WVAMAPKPGPYVK | 0.9953 | 1586.8431 | Q01518 |
| WVAMAPKPGPYVK | 0.9826 | 1586.8431 | Q01518 |
| WVVSAAHCYK | 0.8515 | 1335.6182 | P35030 |
| WVVSAAHCYK | 0.8042 | 1335.6182 | P35030 |
| YADPVSAQHAK | 0.9995 | 1301.6152 | P26599 |
| YAGAAVDELGK | 0.7825 | 1208.5825 | P30086 |

| **Identified Peptides (prime sequence)** | **PeptideProphet probability** | **Neutral peptide mass (Da)** | **Exemplary protein ID** |
| --- | --- | --- | --- |
| YAHELPK | 0.9609 | 972.4817 | P46777 |
| YALPHAILR | 0.9887 | 1140.6192 | Q562R1 |
| YEGYALPHAILR | 0.9998 | 1489.7466 | Q562R1 |
| YLVPILTQTLTK | 0.8965 | 1504.8653 | Q14974 |
| YMVGPIEEAVAK | 0.9512 | 1421.7013 | P06576 |
| YSCVGVFQHGK | 0.7349 | 1396.6346 | P34931 |
| YTLIVRPDNTYEVK | 0.9734 | 1825.9362 | P27797 |
| YVELQKEEAQK | 0.9996 | 1507.7670 | Q00839 |
| YVELQKEEAQK | 0.9305 | 1507.7670 | Q00839 |
| YVELQKEEAQK | 0.9916 | 1507.7597 | Q00839 |
| YVELQKEEAQK | 0.9663 | 1507.7597 | Q00839 |
| YVLLLMGAFS | 0.9045 | 1216.5876 | P37296 |
| YVRPLPPAAIESPAVAAPAYSR | 0.9838 | 2383.2436 | P04792 |
| YVRPLPPAAIESPAVAAPAYSR | 0.9985 | 2383.2436 | P04792 |
| YYLLSGAGEHLK | 0.8881 | 1465.7353 | P35579 |
