# Supplementary material for: RC1339/APRc from Rickettsia conorii Is a Novel Aspartic Protease with Properties of Retropepsin-Like Enzymes
Source: PLoS Pathog. 2014 Aug 21;10(8):e1004324. doi: 10.1371/journal.ppat.1004324 (PMC4140852; doi:10.1371/journal.ppat.1004324)
Supplement: Table S2 — APRc cleavage sites identified from a GluC peptide library using Mascot and X!Tandem. Peptides identified by LC-MS/MS spectrum-to-sequence assignment with Mascot and X!Tandem are listed with PeptideProphet probability score, calculated neutral mass and one exemplary accession number of a matching UniProt protein entry is listed. This data was further processed and rendered non-redundant for generation of cleavage specificity profiles. (DOCX) [file ppat.1004324.s004.docx]

| **Identified Peptides (prime sequence)** | **PeptideProphet probability** | **Neutral peptide mass (Da)** | **Exemplary protein ID** |
| --- | --- | --- | --- |

| AAAALAAAAVK | 0.9913 | 1042.5923 | Q8TAQ2 |
| --- | --- | --- | --- |
| AAAGYDVEKNNSR | 0.9739 | 1509.6960 | Q02539 |
| AAAIAYGLDK | 0.8871 | 1107.5712 | P11021 |
| AAAIAYGLDKK | 0.9997 | 1263.6975 | P54652 |
| AAEKLQVVGR | 0.9851 | 1185.6618 | O43175 |
| AAGAGATHSPPTDLVWK | 0.9989 | 1793.8849 | P02545 |
| AAGGGREHALR | 0.8320 | 1181.5802 | Q6ZRF8-3 |
| AALKNPPINTK | 0.8569 | 1309.7506 | O15511 |
| AALLALQHKAE | 0.8604 | 1279.7036 | Q15154 |
| AASLLGKK | 0.8823 | 930.5650 | P49321 |
| AATLEVERPLPMEVEK | 0.9959 | 1926.9873 | P51858 |
| ADIETIGEILKK | 0.8278 | 1472.8238 | P61978 |
| AEAMNYEGSPIKVTLATLK | 0.9993 | 2179.1347 | P06748 |
| AEQLKNQIR | 0.8820 | 1214.6519 | P62873 |
| AEQLKNQIR | 0.8642 | 1214.6519 | P62873 |
| AFEISKK | 0.8685 | 965.5334 | P31946 |
| AFEISKK | 0.9468 | 965.5334 | P31946 |
| AFQLFDRTGDGK | 0.8260 | 1469.7051 | P60660 |
| AFWIDKIK | 0.9037 | 1163.6491 | Q9UKV3 |
| AGALVLADR | 0.8253 | 972.5141 | P49736 |
| AGPTALLAHEIGFGSK | 0.8372 | 1683.8732 | Q99497 |
| AGPVAEYLK | 0.9993 | 1062.5498 | Q01518 |
| AHLDATTVLSR | 0.9977 | 1270.6418 | P06576 |
| AIAEAWAR | 0.9708 | 974.4722 | Q71U36 |
| AKDAFLGSFLYE | 0.9669 | 1475.7007 | P02769 |
| AKPFVPNVHAAE | 0.9961 | 1394.7095 | Q8IYD1 |
| AKQIVWNGPVGVFE | 0.9815 | 1658.8569 | P00558 |
| ALALFGGEPK | 0.8784 | 1117.5920 | P49736 |
| ALCSLHSIGK | 0.9605 | 1200.6073 | P14174 |
| ALFAQLNQGE | 0.9944 | 1177.5516 | P40123 |
| ALFAQLNQGE | 0.9764 | 1177.5516 | P40123 |
| ALGWVAMAPKPGPYVK | 0.9990 | 1827.9858 | Q01518 |
| ALGWVAMAPKPGPYVK | 0.9940 | 1827.9858 | Q01518 |

| **Identified Peptides (prime sequence)** | **PeptideProphet probability** | **Neutral peptide mass (Da)** | **Exemplary protein ID** |
| --- | --- | --- | --- |
| ALKLESCGVTSDNCR | 0.9992 | 1824.8247 | P13489 |
| ALLSLAKGDRSE | 0.9374 | 1374.7255 | P04083 |
| ALNGKEVAAQVK | 0.8218 | 1370.7670 | Q99497 |
| ALPHAILR | 0.9219 | 977.5558 | Q562R1 |
| ALPHAILRLD | 0.9854 | 1205.6669 | Q562R1 |
| ALPHAILRLD | 0.9975 | 1205.6669 | Q562R1 |
| ALPHAILRLD | 0.9941 | 1205.6669 | Q562R1 |
| ALPHAILRLD | 0.9268 | 1205.6669 | Q562R1 |
| ALQDMLLLK | 0.9084 | 1175.6372 | Q6P2M8-5 |
| ALSRQLSSGVSE | 0.9417 | 1320.6348 | P04792 |
| AQINQGESITHALK | 0.9988 | 1624.8321 | Q01518 |
| ASGKQEPEAK | 0.8777 | 1187.5934 | Q92797 |
| ASLAAAKK | 0.8668 | 902.5337 | P20700 |
| ASPAPVK | 0.9154 | 784.4157 | Q2N9J7 |
| ASQCQQPAENK | 0.9975 | 1375.5938 | Q01518 |
| ASSSSLEKSYELPDGQVITIGNER | 0.9989 | 2695.3089 | P62736 |
| ATIIDILTKR | 0.9912 | 1258.7397 | P04083 |
| AVALAGLLAAQK | 0.8766 | 1240.7291 | P23368 |
| AVLLGPPGAGKGTQAPRLAE | 0.9979 | 2018.0987 | P54819 |
| AVLPPLPKRPALE | 0.8666 | 1515.8925 | Q9NR56 |
| AVLVALKRAQSE | 0.8888 | 1399.7935 | P25786 |
| AVRLLLPGE | 0.9168 | 1054.5923 | Q96A08 |
| AVSSPPPADLCHALR | 0.8942 | 1677.8045 | P24534 |
| AVTYTEHAK | 0.9770 | 1134.5458 | P62805 |
| AVVTVPAYFND | 0.9690 | 1282.5982 | P11021 |
| C[143 | 0.9742 | 2078.6941 | A0JP86 |
| CELINALYPEGQAPVKK | 0.9871 | 2073.0717 | P37802 |
| CIAIKESAK | 0.9978 | 1162.6168 | P61158 |
| CLAPLAKVIHD | 0.9051 | 1351.7070 | P04406 |
| DANLQTLTEYLKK | 0.9511 | 1679.8882 | P55060 |
| DANTIVCNSKDGGAWGTEQR | 0.9984 | 2294.0134 | P09382 |
| DCHTAHIACK | 0.9807 | 1327.5550 | P68104 |
| DEELNKLLGK | 0.9736 | 1301.6979 | P20671 |
| DEGGFAPNILENKEGLELLK | 0.9965 | 2329.1953 | P06733 |
| DEITYVELQKEEAQK | 0.9991 | 1965.9683 | Q00839 |
| DESGPSIVHR | 0.9749 | 1183.5370 | Q9BYX7 |
| DESTGSIAKR | 0.9683 | 1178.5679 | P04075 |
| DFLLKPELLR | 0.9396 | 1358.7710 | O00148 |
| DGCHAYLSKNSLDCE | 0.9439 | 1883.7566 | Q01518 |

| **Identified Peptides (prime sequence)** | **PeptideProphet probability** | **Neutral peptide mass (Da)** | **Exemplary protein ID** |
| --- | --- | --- | --- |
| DIAVDGEPLGR | 0.9202 | 1228.5836 | P62937 |
| DISPQAPTHFLVIPK | 0.9703 | 1777.9515 | P49773 |
| DKANAQAAALYK | 0.9967 | 1406.7306 | P40121 |
| DKANAQAAALYKVSD | 0.9983 | 1707.858 | P40121 |
| DKGLQTSQDAR | 0.9522 | 1333.6374 | P27797 |
| DKVSHVSTGGGASLE | 0.9249 | 1558.7297 | P00558 |
| DKYLIPNATQPESK | 0.8801 | 1746.8940 | P31946 |
| DLFRGTLDPVE | 0.9731 | 1348.6411 | P11142 |
| DNSSRPSQVVAETR | 0.8458 | 1632.7604 | P13639 |
| DQAQKAEGAGDAK | 0.8576 | 1431.6742 | P05204 |
| DQATSLRILNNGHAFNVE | 0.8455 | 2085.998 | P00918 |
| DQLHAAVGASR | 0.9841 | 1211.5795 | P13804 |
| DQSYKPDENEVR | 0.9941 | 1594.7011 | P31939 |
| DRTAGIGGMNHFMLPD | 0.9590 | 1834.7807 | Q13SY1 |
| DRTVIDYNGER | 0.9855 | 1424.6432 | P07237 |
| DSLLAGPVAEYLK | 0.9404 | 1490.7769 | Q01518 |
| DSLYVEKIDVGEAEPR | 0.9038 | 1934.9373 | P54577 |
| DSYVGDEAQSKR | 0.9958 | 1469.6535 | P62736 |
| DTFWKEFGTNIK | 0.8305 | 1628.7987 | Q58FF3 |
| DTKPGTTGSGAGSGGPGGLTSAAPAGGDKK | 0.9918 | 2728.3417 | P67809 |
| DTYNCDLHFK | 0.9169 | 1427.5928 | Q9BUJ2 |
| DVVVLPGGNLGAQNLSESAAVK | 0.9570 | 2253.1753 | Q99497 |
| DVVYALKR | 0.9217 | 1078.5923 | P62805 |
| E[111 | 0.8931 | 1406.6544 | Q05778 |
| EAPLNPKANRE | 0.9360 | 1353.6717 | P18600 |
| EDTNLCAIHAK | 0.9762 | 1386.6350 | P68431 |
| EYIPHADLRLI | 0.8602 | 1426.7283 | P66648 |
| FAEALAAHK | 0.9996 | 1072.5453 | P07237 |
| FALLEIPK | 0.8667 | 1045.5960 | O94915 |
| FAQINQGESITHALK | 0.9998 | 1771.9005 | Q01518 |
| FAQINQGESITHALK | 0.9972 | 1771.9005 | Q01518 |
| FASKPAAR | 0.9668 | 962.5086 | P23246 |
| FCSEYRPK | 0.9233 | 1201.5338 | P09429 |
| FFVQTCR | 0.9814 | 1044.4599 | B2RPK0 |
| FGPLKAFNLVKD | 0.9217 | 1491.8238 | P26368 |
| FHFEPNEYFTNEVLTK | 0.9286 | 2129.9846 | P55209 |
| FIAIKPDGVQRGLVGE | 0.9065 | 1813.9839 | P15531 |
| FIFIDSDHTDNQR | 0.9454 | 1694.7437 | P07237 |
| FILFKDAASVEK | 0.9954 | 1510.8184 | Q99729 |

| **Identified Peptides (prime sequence)** | **PeptideProphet probability** | **Neutral peptide mass (Da)** | **Exemplary protein ID** |
| --- | --- | --- | --- |
| FINNPLAQAD | 0.9741 | 1189.5516 | P35579 |
| FKELVYPPDYNPEGK | 0.9917 | 1938.9516 | P12956 |
| FLQTPKIVADKD | 0.8935 | 1517.8242 | P07195 |
| FLQTPKIVADKD | 0.8676 | 1517.8242 | P07195 |
| FMILPVGAANFR | 0.9942 | 1422.7230 | P06733 |
| FMVVNDAGRPK | 0.9976 | 1348.6710 | P11142 |
| FMVVNDAGRPK | 0.9928 | 1348.6710 | P11142 |
| FNVINGGSHAGNKLAMQE | 0.9883 | 2001.9479 | P06733 |
| FNVINGGSHAGNKLAMQE | 0.8442 | 2001.9479 | P06733 |
| FSAPKPQTSPSPK | 0.9948 | 1514.7881 | Q01518 |
| FSQIVRVLTEDE | 0.9906 | 1522.7337 | P14324 |
| FSTPLLLGKK | 0.8938 | 1246.7437 | P40926 |
| FTTNLTEEEEKSK | 0.9321 | 1698.8100 | P35579 |
| FVALSTNTTKVKE | 0.9865 | 1580.8562 | P06744 |
| FVTFCTK | 0.9503 | 1017.4742 | O60506 |
| FVTFDDHDPVDK | 0.9668 | 1549.6838 | P22626 |
| FYAPWCGHCQR | 0.8242 | 1568.6190 | Q15084 |
| FYIITNKLKE | 0.9466 | 1411.7863 | Q9NTJ3 |
| FYVNGLTLGGQK | 0.9986 | 1411.7248 | P07737 |
| FYVNGLTLGGQK | 0.9883 | 1411.7248 | P07737 |
| FYVNGLTLGGQKCSVIRD | 0.998 | 2142.068 | P07737 |
| GAFQHVGK | 0.9111 | 958.4773 | P26641 |
| GAGLMGAGIAQVSVDKGLK | 0.9604 | 1915.0349 | P40939 |
| GAPFLKEGASEEEIR | 0.9123 | 1747.8529 | P23141 |
| GCHAYLSKNSLDCE | 0.9738 | 1768.7297 | Q01518 |
| GFTLPHAILRLD | 0.9621 | 1439.7597 | P0C542 |
| GFTLPHAILRLD | 0.8971 | 1439.7597 | P0C542 |
| GGSHAGNKLAMQE | 0.9843 | 1430.6360 | P06733 |
| GGSHAGNKLAMQE | 0.9482 | 1414.6411 | P06733 |
| GGTTMYPGIADRMQKE | 0.9948 | 1869.8501 | P62736 |
| GGTTMYPGIADRMQKE | 0.9240 | 1869.8501 | P62736 |
| GGTTMYPGIADRMQKE | 0.9773 | 1869.8427 | P18600 |
| GHSLGTGVATNLVR | 0.8227 | 1468.7535 | Q8N2K0 |
| GHVLAAGCGQNPVR | 0.9947 | 1522.7211 | Q9BWD1 |
| GIHETTFNSIMK | 0.9341 | 1492.7132 | Q562R1 |
| GILTLKYPIE | 0.9909 | 1261.7070 | P62736 |
| GIMNSFVNDIFERIAGE | 0.9345 | 1998.9187 | P33778 |
| GINLVQAKKLVE | 0.9807 | 1454.8609 | P52815 |
| GKEILVGDVGQTVDDPYATFVK | 0.9997 | 2494.2744 | P23528 |

| **Identified Peptides (prime sequence)** | **PeptideProphet probability** | **Neutral peptide mass (Da)** | **Exemplary protein ID** |
| --- | --- | --- | --- |
| GKVLPGVDALSNI | 0.9048 | 1397.7597 | P00558 |
| GKVLPGVDALSNI | 0.9670 | 1397.7597 | P00558 |
| GLAWSKTGPVAKE | 0.9980 | 1486.7932 | Q01518 |
| GLTLGGQKCSVIRD | 0.9841 | 1618.8249 | P07737 |
| GLTSVINQKLKDDE | 0.9579 | 1702.8817 | P07195 |
| GLTSVINQKLKDDE | 0.9769 | 1702.8817 | P07195 |
| GLTSVINQKLKDDE | 0.9201 | 1702.8817 | P07195 |
| GLTSVINQKLKDDE | 0.9671 | 1702.8817 | P07195 |
| GPIKTTE | 0.9347 | 860.4318 | P24487 |
| GQKDSYVGDEAQSK | 0.9999 | 1654.7587 | P62736 |
| GQLLTSSNYDDDEKK | 0.9563 | 1855.8588 | P11388 |
| GQSGAGNNWAK | 0.9782 | 1204.5373 | Q13885 |
| GTLLKPNMVTPGHACTQK | 0.9931 | 2096.0659 | P04075 |
| GTLLKPNMVTPGHACTQK | 0.9959 | 2096.0659 | P04075 |
| GVDLLADAVAVTMGPK | 0.9083 | 1671.8654 | P10809 |
| GVGILALIDALRDNE | 0.9607 | 1655.8557 | Q9NYL9 |
| GVLPNIQAVLLPK | 0.9213 | 1476.8816 | Q96QV6 |
| GVLPNIQAVLLPKKTE | 0.9988 | 1863.0982 | Q96QV6 |
| GVMVGMGQKDSYVGDE | 0.9937 | 1786.758 | P18600 |
| GVPLSDPVPDPE | 0.8852 | 1308.5912 | A1T700 |
| GVPMPDKYSLEPVAVELK | 0.8538 | 2115.1074 | P00558 |
| GVSLAVCK | 0.9001 | 948.4851 | P06733 |
| GVSLKTLHPD | 0.8475 | 1181.6193 | P00338 |
| GYALPHAILR | 0.8288 | 1197.6406 | Q562R1 |
| GYALPHAILR | 0.9661 | 1197.6406 | Q562R1 |
| GYALPHAILR | 0.9923 | 1197.6406 | Q562R1 |
| GYALPHAILR | 0.9258 | 1197.6406 | Q562R1 |
| GYALPHAILR | 0.9699 | 1197.6406 | Q562R1 |
| GYALPHAILR | 0.9990 | 1197.6406 | Q562R1 |
| GYNYTGMGNSTNKK | 0.9927 | 1677.7569 | Q08211 |
| HEKYDNSLK | 0.8692 | 1276.6200 | P04406 |
| HGIQPDGQMPSDK | 0.9991 | 1524.6779 | Q71U36 |
| HLATGDMLR | 0.9932 | 1100.5185 | P54819 |
| HLQLAIRNDEE | 0.9959 | 1424.6796 | Q96QV6 |
| HLQLAIRNDEE | 0.9172 | 1424.6796 | Q96QV6 |
| HLQLAVRNDEE | 0.9905 | 1410.6640 | Q8IUE6 |
| HSAVSLDPIKSFE | 0.9687 | 1544.7623 | Q9Y3F4 |
| HVVVPVNPK | 0.8494 | 1103.6240 | Q92499 |
| IAALVIDNGSGMCK | 0.9985 | 1579.7486 | P63261 |

| **Identified Peptides (prime sequence)** | **PeptideProphet probability** | **Neutral peptide mass (Da)** | **Exemplary protein ID** |
| --- | --- | --- | --- |
| IAALVIDNGSGMCK | 0.9918 | 1579.7486 | P63261 |
| IAALVIDNGSGMCK | 0.9999 | 1563.7537 | P63261 |
| IAALVIDNGSGMCK | 0.9997 | 1563.7537 | P63261 |
| IAALVIDNGSGMCK | 0.9920 | 1563.7537 | P63261 |
| IAALVIDNGSGMCK | 0.9920 | 1563.7537 | P63261 |
| IAALVIDNGSGMCK | 0.9998 | 1563.7537 | P63261 |
| IAALVIDNGSGMCK | 0.9993 | 1563.7537 | P63261 |
| IAAQYSGAQVR | 0.9996 | 1250.6156 | P26641 |
| IAEAYLGK | 0.9981 | 979.5126 | P11142 |
| IAMATVTALR | 0.9983 | 1133.6015 | P04075 |
| IANLFNRYPALHKPE | 0.9901 | 1897.9951 | P13796 |
| IAPALVSKKLNVTE | 0.9967 | 1625.9504 | P06733 |
| IAPALVSKKLNVTE | 0.9985 | 1625.9504 | P06733 |
| IAPALVSKKLNVTE | 0.9982 | 1625.9504 | P06733 |
| IAPALVSKKLNVTE | 0.9988 | 1625.9504 | P06733 |
| IAPALVSKKLNVTE | 0.9988 | 1625.9504 | P06733 |
| IAPALVSKKLNVTE | 0.9926 | 1625.9504 | P06733 |
| IAPALVSKKLNVTE | 0.9962 | 1625.9504 | P06733 |
| IAPALVSKKLNVTE | 0.9938 | 1625.9504 | P06733 |
| IAPALVSKKLNVTE | 0.9919 | 1625.9504 | P06733 |
| IAPALVSKKLNVTE | 0.9985 | 1625.9504 | P06733 |
| IAPALVSKKLNVTE | 0.9715 | 1625.9504 | P06733 |
| IAPALVSKKLNVTE | 0.9799 | 1625.9504 | P06733 |
| IAPALVSKKLNVTE | 0.9858 | 1625.9504 | P06733 |
| IAPALVSKKLNVTE | 0.9326 | 1625.9504 | P06733 |
| IAQGGVLPNIQAVLLPKKTE | 0.9916 | 2232.2994 | Q96QV6 |
| IAQVDPKK | 0.9416 | 1041.5971 | Q9Y2B0 |
| IASGGVLPNIHPELLAK | 0.9903 | 1844.0308 | O75367 |
| IFIDSDHTDNQR | 0.9933 | 1547.6753 | P07237 |
| IFIDSDHTDNQRILE | 0.9106 | 1902.886 | P07237 |
| IFMAIAK | 0.9043 | 908.4942 | P20339 |
| IGAIAIGDLVK | 0.9975 | 1184.6917 | P78371 |
| IGGIGTVPVGRVE | 0.9987 | 1340.7201 | P68104 |
| IGLAKDDQLK | 0.8252 | 1243.6924 | P41567 |
| IGNLNTLVVKKSDVE | 0.9871 | 1771.9832 | O60812 |
| IITTEKTSK | 0.9421 | 1163.6550 | P40939 |
| ILFLDPSGKVHPE | 0.9712 | 1566.8194 | O95881 |
| ILLVQPTKRPE | 0.9625 | 1408.8190 | P84090 |
| ILTAFQK | 0.8309 | 935.5228 | P30040 |

| **Identified Peptides (prime sequence)** | **PeptideProphet probability** | **Neutral peptide mass (Da)** | **Exemplary protein ID** |
| --- | --- | --- | --- |
| ILTHGIFSGPAISR | 0.9912 | 1555.8259 | P60891 |
| ILTLKYPIE | 0.9596 | 1204.6855 | P62736 |
| ILTLKYPIE | 0.9153 | 1204.6855 | P62736 |
| ILTLKYPIE | 0.9856 | 1204.6855 | P62736 |
| ILTLKYPIE | 0.9931 | 1204.6855 | P62736 |
| ILTLKYPIE | 0.9698 | 1204.6855 | P62736 |
| ILTLKYPIE | 0.9721 | 1204.6855 | P62736 |
| ILTLKYPIE | 0.9762 | 1204.6855 | P62736 |
| IMFGPDKCGE | 0.9932 | 1268.5318 | P27824 |
| INAISKK | 0.8596 | 916.5494 | P61158 |
| INPDHPIVETLR | 0.9799 | 1490.7630 | P08238 |
| IQAVLLPKKTE | 0.9961 | 1382.8285 | Q96QV6 |
| IQAVLLPKKTE | 0.9959 | 1382.8285 | Q96QV6 |
| IQAVLLPKKTE | 0.9987 | 1382.8285 | Q96QV6 |
| IQAVLLPKKTE | 0.9980 | 1382.8285 | Q96QV6 |
| IQAVLLPKKTE | 0.9991 | 1382.8285 | Q96QV6 |
| IQAVLLPKKTE | 0.9399 | 1382.8285 | Q96QV6 |
| IQAVLLPKKTE | 0.9575 | 1382.8285 | Q96QV6 |
| IQAVLLPKKTE | 0.9968 | 1382.8285 | Q96QV6 |
| IQAVLLPKKTE | 0.9955 | 1382.8285 | Q96QV6 |
| IQAVLLPKKTE | 0.8761 | 1382.8285 | Q96QV6 |
| IQAVLLPKKTE | 0.9907 | 1382.8285 | Q96QV6 |
| IQAVLLPKKTE | 0.9986 | 1382.8285 | Q96QV6 |
| IQAVLLPKKTE | 0.9961 | 1382.8285 | Q96QV6 |
| IQAVLLPKKTE | 0.9952 | 1382.8285 | Q96QV6 |
| IQAVLLPKKTE | 0.9925 | 1382.8285 | Q96QV6 |
| IQAVLLPKKTE | 0.9940 | 1382.8285 | Q96QV6 |
| IQAVLLPKKTE | 0.9908 | 1382.8285 | Q96QV6 |
| IQAVLLPKKTE | 0.9273 | 1382.8285 | Q96QV6 |
| IQAVLLPKKTE | 0.9236 | 1382.8285 | Q96QV6 |
| IQGITKPAIR | 0.9929 | 1211.7138 | P62805 |
| IQGLTTAHEQFK | 0.9788 | 1487.7521 | P12814 |
| IQNAPEQACHLAK | 0.9862 | 1594.7674 | P61981 |
| IRNDEELNK | 0.9681 | 1245.6101 | Q96QV6 |
| ISADIETIGEILKK | 0.8922 | 1672.9399 | P61978 |
| ISFGTTKDK | 0.8673 | 1139.5975 | P48643 |
| ISHLIEPLANAAR | 0.9990 | 1491.7946 | Q9Y490 |
| ISLPIHPMITNVAK | 0.9714 | 1648.9123 | Q14204 |
| ISPYFINTSKGQKCE | 0.9990 | 1914.9298 | P10809 |

| **Identified Peptides (prime sequence)** | **PeptideProphet probability** | **Neutral peptide mass (Da)** | **Exemplary protein ID** |
| --- | --- | --- | --- |
| ISPYFINTSKGQKCE | 0.9980 | 1914.9298 | P10809 |
| ITYTDEEPVKK | 0.9341 | 1465.7453 | O14979 |
| ITYTDEEPVKKLLE | 0.9168 | 1820.9560 | O14979 |
| IVKLPLLPHE | 0.9352 | 1273.7546 | O43681 |
| IVTNWDDME | 0.8470 | 1225.4709 | P60709 |
| IYGMEGIPEKDMDER | 0.9813 | 1897.8338 | O43670 |
| IYTNYEAGKDDYVK | 0.9566 | 1821.8573 | P09211 |
| KACANPAAGSVILLE | 0.9874 | 1628.8267 | P00558 |
| KACANPAAGSVILLE | 0.9028 | 1628.8267 | P00558 |
| KACQSIYPLHD | 0.8469 | 1446.664 | Q801S3 |
| KACSLAKTAFDE | 0.9332 | 1483.7057 | P68250 |
| KACSLAKTAFDE | 0.9819 | 1483.7057 | P68250 |
| KADGIVSKNF | 0.8425 | 1221.6506 | P63220 |
| KAGAAPYVQAFD | 0.8415 | 1352.6437 | Q01518 |
| KAPLDIPVPDPVKE | 0.9922 | 1660.9188 | Q06323 |
| KAPNLKILNLSGNE | 0.9434 | 1653.9202 | Q9UBU9 |
| KDDAMLLK | 0.9249 | 1076.5688 | P10809 |
| KDFKAAID | 0.9290 | 1050.5498 | Q155Q3 |
| KDGLILTSRGPGTSFE | 0.9306 | 1792.9037 | Q5E946 |
| KDSPSVWAAVPGK | 0.9997 | 1484.7776 | P07737 |
| KDSTLIMQLLR | 0.9919 | 1432.7860 | P31946 |
| KFLIPNASQAE | 0.9894 | 1332.6752 | P63103 |
| KFLIPNASQAE | 0.9947 | 1332.6752 | P63103 |
| KGTVQQADE | 0.8771 | 1090.5043 | P32969 |
| KHLIPAANTGE | 0.9823 | 1265.6442 | P62261 |
| KHTGPNSPDTAND | 0.9990 | 1468.6331 | P31943 |
| KHTGPNSPDTAND | 0.9993 | 1468.6331 | P31943 |
| KKISSIQSIVPALE | 0.9132 | 1655.9537 | P10809 |
| KLAPVPFFSLLQYE | 0.8471 | 1766.9317 | P07741 |
| KLCYVALDFEQE | 0.9410 | 1629.7427 | P18600 |
| KLFIGGLSFE | 0.9194 | 1225.6421 | Q32P51 |
| KLIAPVAEEE | 0.8401 | 1213.6267 | P07195 |
| KLRIYFLE | 0.8590 | 1196.6705 | Q96SN8 |
| KLSDLLAPISE | 0.9043 | 1300.6957 | Q01518 |
| KMINLSVPDTIDE | 0.9941 | 1589.7687 | P13796 |
| KMINLSVPDTIDE | 0.9604 | 1589.7687 | P13796 |
| KMSVQPTVSLGGFE | 0.9106 | 1594.7813 | P06748 |
| KMSVQPTVSLGGFE | 0.9985 | 1594.7737 | Q3T160 |
| KMVADGVEP | 0.9440 | 1076.4886 | A0M380 |

| **Identified Peptides (prime sequence)** | **PeptideProphet probability** | **Neutral peptide mass (Da)** | **Exemplary protein ID** |
| --- | --- | --- | --- |
| KNLSDLIDLVPSLCE | 0.9525 | 1830.9111 | P79136 |
| KNNQITNNQR | 0.9963 | 1344.6646 | P00558 |
| KQDRTLTIVD | 0.8987 | 1303.6884 | Q58FG1 |
| KQGQDNLSSVKE | 0.9905 | 1475.7368 | P30040 |
| KQGQDNLSSVKE | 0.9745 | 1475.7368 | P30040 |
| KSPLLQLPHIEE | 0.8584 | 1518.8194 | Q9UGP8 |
| KTVQLRNGNLQYD | 0.9863 | 1663.8430 | P06396 |
| KVCNPIITK | 0.9752 | 1215.6798 | P11142 |
| KVLSLLALVKPE | 0.9380 | 1452.8997 | Q9UL46 |
| KYDPSLKPLSVSYD | 0.9152 | 1754.8879 | P00918 |
| KYTLPPGVDPTQVSSSLSPE | 0.9964 | 2217.0953 | P04792 |
| LAGHQTSAESWGTGR | 0.9727 | 1644.7393 | P36578 |
| LAGPTNAIFK | 0.8435 | 1146.6185 | Q15366 |
| LAGPVAEYLK | 0.9988 | 1175.6338 | Q01518 |
| LAGPVAEYLK | 0.9995 | 1175.6338 | Q01518 |
| LAIIDPGDSDIIR | 0.8760 | 1484.7623 | P62888 |
| LAIVEALNGKEVAAQVK | 0.9999 | 1896.0832 | Q99497 |
| LALIDKQE | 0.9176 | 1044.5603 | Q9UFN0 |
| LALLDGSNVVFK | 0.9958 | 1390.7608 | O15212 |
| LAPLAKVIHD | 0.9088 | 1191.6764 | P04406 |
| LAPSTMKIKIIAPPE | 0.9895 | 1752.0007 | P62736 |
| LAQVLAQERPK | 0.9990 | 1367.7673 | P49327 |
| LATATGAK | 0.8390 | 847.4552 | Q9BZH6 |
| LAVDAVIAELK | 0.9986 | 1256.7128 | P10809 |
| LAVDAVIAELKK | 0.9995 | 1412.8391 | P10809 |
| LAWSKTGPVAKE | 0.9912 | 1429.7717 | Q01518 |
| LAWSKTGPVAKE | 0.9891 | 1429.7717 | Q01518 |
| LCAIHAK | 0.9092 | 927.4748 | P68431 |
| LFADKVPK | 0.8575 | 1060.6069 | P62937 |
| LFAEFGTLKK | 0.9604 | 1296.7230 | Q86V81 |
| LFAEFGTLKK | 0.8950 | 1296.7230 | Q86V81 |
| LFHQQGTPR | 0.9887 | 1170.5682 | P20700 |
| LFLPEEYPMAAPK | 0.9695 | 1620.8010 | P61088 |
| LGAYCGYSAVR | 0.9461 | 1303.5767 | P21964 |
| LGGPEAAKSDETAAK | 0.9994 | 1587.7892 | P04792 |
| LGIILAHTNLR | 0.9995 | 1307.7462 | P31939 |
| LGIPFAKPPLGPLR | 0.9231 | 1590.9398 | P23141 |
| LGPKPEVAQQTR | 0.8426 | 1438.7680 | P53621 |
| LGPLVSKVKE | 0.8785 | 1212.7230 | Q86VP6 |

| **Identified Peptides (prime sequence)** | **PeptideProphet probability** | **Neutral peptide mass (Da)** | **Exemplary protein ID** |
| --- | --- | --- | --- |
| LIINSLYKNKE | 0.9706 | 1477.8292 | P14625 |
| LIINSLYKNKE | 0.9920 | 1477.8292 | P14625 |
| LIINSLYKNKE | 0.9772 | 1477.8292 | P14625 |
| LIINTFYSNKE | 0.9733 | 1456.735 | Q58FF8 |
| LIQTADQLR | 0.9734 | 1144.5988 | P18031 |
| LISVYSEKGESSGK | 0.9402 | 1626.8253 | P36578 |
| LIVLEGVDR | 0.9786 | 1100.5978 | P23919 |
| LIVPDNPPYDKGAFRIE | 0.9091 | 2059.0527 | P68036 |
| LIYTNYEAGKDDYVK | 0.9994 | 1934.9414 | P09211 |
| LKAPLDIPVPDPVKE | 0.9976 | 1774.0029 | Q06323 |
| LKEDQTEYLEER | 0.9823 | 1667.7790 | Q58FF7 |
| LKKAGGANYDAQTE | 0.9917 | 1608.7817 | Q2HJ57 |
| LKKAGGANYDAQTE | 0.9743 | 1608.7817 | Q2HJ57 |
| LKQEVISTSSK | 0.8889 | 1362.7507 | P63244 |
| LLAAEFLK | 0.9989 | 1019.5803 | Q99832 |
| LLAKNLPYKVTQDE | 0.8899 | 1774.9617 | P19338 |
| LLALVKPE | 0.8901 | 997.5960 | Q9UL46 |
| LLAYTLGVK | 0.9190 | 1092.6331 | P68104 |
| LLDKYLIPNATQPESK | 0.9954 | 1973.0621 | P31946 |
| LLIGPRGNTLKNIE | 0.8990 | 1652.9362 | Q15637 |
| LLKQGQDNLSSVKE | 0.9443 | 1701.9049 | P30040 |
| LLKQGQDNLSSVKE | 0.8636 | 1701.9049 | P30040 |
| LLPAIVHINHQPFLE | 0.9191 | 1827.9784 | P17844 |
| LLTSFGPLK | 0.9887 | 1090.6175 | P26368 |
| LLVVTDPRADHQPLTE | 0.9264 | 1890.9588 | P08865 |
| LMTPAACPEPPPEAPTEDDHDEL | 0.9787 | 2619.0893 | P14314 |
| LNILTAFQKKGAE | 0.9967 | 1575.8773 | P30040 |
| LNMLSLK | 0.9704 | 933.5105 | Q9Y617 |
| LNVVDIAGLVK | 0.9988 | 1255.7288 | Q9NTK5 |
| LQANCYEEVKDR | 0.9711 | 1639.7412 | P23528 |
| LQGIPVLVLGNKR | 0.9884 | 1521.9143 | Q96BM9 |
| LQKYPPPLIPPRGE | 0.8925 | 1719.9460 | P25098 |
| LQLAIRNDEE | 0.9625 | 1287.6207 | Q96QV6 |
| LQLFRGDTVLLK | 0.9998 | 1517.8718 | P55072 |
| LQTVAKNKDQGTYE | 0.9453 | 1737.8686 | P60660 |
| LQTVAKNKDQGTYE | 0.9641 | 1737.8686 | P60660 |
| LSNLKAPLDIPVPDPVKE | 0.9843 | 2088.1619 | Q06323 |
| LSQLQKQLAAKE | 0.8444 | 1499.8459 | P02545 |
| LVEAIVLPMNHK | 0.9434 | 1478.8067 | P17980 |

| **Identified Peptides (prime sequence)** | **PeptideProphet probability** | **Neutral peptide mass (Da)** | **Exemplary protein ID** |
| --- | --- | --- | --- |
| LVFLPFADDKR | 0.9643 | 1435.7612 | P12956 |
| LVTASQCQQPAENK | 0.9662 | 1688.7940 | Q01518 |
| LVVLLQANRDPDAGIDE | 0.9957 | 1924.9567 | P08758 |
| LVVLLQANRDPDAGIDE | 0.9907 | 1924.9567 | P08758 |
| LVVLLQANRDPDAGIDE | 0.9874 | 1924.9567 | P08758 |
| LVVLLQANRDPDAGIDE | 0.9950 | 1924.9568 | P08758 |
| LVWVPSDKSGFEPASLKE | 0.9955 | 2132.0942 | P35579 |
| LVYQEPIPTAQLVQR | 0.9957 | 1841.9788 | P25787 |
| LYCLEHGIQPDGQMPSDK | 0.9412 | 2202.9826 | Q71U36 |
| LYTLIVRPDNTYE | 0.9211 | 1683.8256 | P27797 |
| LYYTGEKGQNQDYR | 0.9834 | 1849.8383 | P19338 |
| MANAGPNTNGSQFFICTAK | 0.9489 | 2143.9567 | A2BFH1 |
| MAPKPGPYVKE | 0.9953 | 1359.6937 | Q01518 |
| MAPKPGPYVKE | 0.9954 | 1359.6937 | Q01518 |
| MATAASSSSLEK | 0.9927 | 1313.5921 | P62736 |
| MATAASSSSLEK | 0.9515 | 1297.5972 | P62736 |
| MATAASSSSLEK | 0.9983 | 1297.5972 | P62736 |
| MATAASSSSLEK | 0.9918 | 1297.5972 | P62736 |
| MGTYATQSALSSSRPTK | 0.9952 | 1900.9101 | P53618 |
| MLMAHAVTQLANR | 0.9993 | 1542.7547 | P78371 |
| MQIQHPTASLIAK | 0.9044 | 1552.8184 | Q92526 |
| MQKLDAQVK | 0.9319 | 1203.6434 | P04843 |
| MRPGVACSVSQAQKDE | 0.9679 | 1877.8512 | P32969 |
| MRPGVACSVSQAQKDE | 0.9683 | 1877.8512 | P32969 |
| MSHLGRPDGVPMPD | 0.9778 | 1595.6973 | P00558 |
| MVPGKPMCVESFSDYPPLGR | 0.9712 | 2382.0959 | P68104 |
| NDGAAALVLMTADAAKR | 0.9671 | 1802.9097 | P24752 |
| NDGATILSMMDVDHQIAK | 0.9993 | 2073.9611 | P48643 |
| NGFLSPDKLSLLEK | 0.9845 | 1703.9246 | P31689 |
| NHIIDGVK | 0.9737 | 1010.5297 | Q9GZT3 |
| NSFVNDIFER | 0.9758 | 1327.5945 | P33778 |
| NSKDGGAWGTEQRE | 0.9945 | 1649.7182 | P09382 |
| NTAVSQLTKAKE | 0.9970 | 1432.7674 | Q9NTJ3 |
| NTHADFADECPKPE | 0.9808 | 1745.7103 | P43487 |
| NVDLSTVDKDQSIAPK | 0.9395 | 1872.9581 | P04844 |
| NVLRQTGNNE | 0.8449 | 1231.5693 | Q9BYX4 |
| NVPLPNTLPLPKRE | 0.9946 | 1702.9518 | Q9Y520 |
| NVSAVDKSTGKE | 0.9855 | 1377.6888 | P11142 |
| NVSAVDKSTGKE | 0.8845 | 1377.6888 | P11142 |

| **Identified Peptides (prime sequence)** | **PeptideProphet probability** | **Neutral peptide mass (Da)** | **Exemplary protein ID** |
| --- | --- | --- | --- |
| QAALKNPPINTK | 0.9139 | 1437.8092 | O15511 |
| QARPDDLLISTYPK | 0.9506 | 1731.8944 | P50225 |
| QGESITHALK | 0.9994 | 1198.6094 | Q01518 |
| QGGVLPNIQAVLLPK | 0.9966 | 1661.9617 | Q96QV6 |
| QGGVLPNIQAVLLPKKTE | 0.9974 | 2048.1782 | Q96QV6 |
| QGLIVPDNPPYDKGAFRIE | 0.9551 | 2244.1327 | P68036 |
| QHGKVEIIANDQGNR | 0.9121 | 1793.8921 | P34931 |
| QIDNPDYKGTWIHPE | 0.9627 | 1927.8853 | P27797 |
| QYLLTLGFK | 0.9079 | 1197.6546 | Q9BXB7 |
| QYLLTLGFK | 0.9159 | 1197.6546 | Q9BXB7 |
| QYLLTLGFK | 0.9251 | 1197.6546 | Q9BXB7 |
| RAQPVQVAE | 0.9954 | 1084.5413 | P06396 |
| RDQNILLGTTYR | 0.9967 | 1536.7797 | P78527 |
| REVPCPPGTE | 0.8986 | 1228.5295 | Q9Y4B4 |
| RIVAPGKGILAADE | 0.8499 | 1524.8412 | P04075 |
| RIVAPGKGILAADE | 0.9391 | 1524.8412 | P04075 |
| RIVILGPE | 0.9135 | 983.5552 | Q5T089 |
| RKAEPEGLR | 0.8924 | 1170.6257 | Q9GZX7 |
| RLCYVALDFEQE | 0.9893 | 1629.7171 | P43239 |
| RLCYVALDFEQE | 0.9859 | 1629.7171 | P43239 |
| RLCYVALDFEQE | 0.9845 | 1629.7171 | P43239 |
| RLCYVALDFEQE | 0.9592 | 1629.7171 | P43239 |
| RNPLIAGK | 0.9790 | 983.5664 | P62316 |
| RPDNFVFGQSGAGNNWAK | 0.9995 | 2079.9663 | Q13885 |
| RPGLEGYALPR | 0.9260 | 1315.6785 | P33992 |
| RRLPLPKP | 0.9056 | 1091.6716 | Q6IE36 |
| RSYELPDGQVITIGNE | 0.9910 | 1877.8833 | Q8BFZ3 |
| RSYELPDGQVITIGNE | 0.9957 | 1877.8833 | Q8BFZ3 |
| RSYELPDGQVITIGNE | 0.9958 | 1877.8833 | Q8BFZ3 |
| RSYELPDGQVITIGNE | 0.9958 | 1877.8833 | Q8BFZ3 |
| RVHIPNDDAQFD | 0.9927 | 1513.6698 | Q16576 |
| RVHIPNDDAQFD | 0.9248 | 1513.6698 | Q16576 |
| SAALIQQATTVK | 0.9840 | 1345.7354 | P32969 |
| SAGIMDHEEAR | 0.9964 | 1302.5411 | P62244 |
| SAIVILRPTKA | 0.9582 | 1283.7639 | Q5JFZ4 |
| SALFAQLNQGE | 0.9963 | 1264.5836 | P40123 |
| SALILHDDE | 0.9627 | 1099.4934 | P05386 |
| SAPKPQTSPSPK | 0.9875 | 1367.7197 | Q01518 |
| SAQLSQLQKQLAAKE | 0.9953 | 1785.9663 | P02545 |

| **Identified Peptides (prime sequence)** | **PeptideProphet probability** | **Neutral peptide mass (Da)** | **Exemplary protein ID** |
| --- | --- | --- | --- |
| SATMPSDVLEVTKK | 0.8444 | 1648.8494 | P60842 |
| SAVPPGADKKAE | 0.9062 | 1312.6701 | Q3T0F4 |
| SCVGVFQHGKVE | 0.9471 | 1461.6823 | P34931 |
| SDNLKFPDLGLKLI | 0.9125 | 1715.9536 | Q57690 |
| SFTLRQQLQTTRQE | 0.9895 | 1822.8997 | Q08E38 |
| SGGGVAMIGVGE | 0.8641 | 1120.4897 | Q58039 |
| SGGTTMYPGIADR | 0.9304 | 1412.6143 | P62736 |
| SGGTTMYPGIADRMQKE | 0.9848 | 1956.8822 | P62736 |
| SGGTTMYPGIADRMQKE | 0.9972 | 1956.8747 | P18600 |
| SGGVTIPP | 0.8890 | 814.3899 | Q5JH10 |
| SGSSHQDLSQR | 0.9733 | 1288.5544 | P11908 |
| SGVTTCLR | 0.9232 | 980.4498 | Q13885 |
| SILGTTLKDE | 0.9704 | 1191.6135 | O75083 |
| SIQALGWVAMAPKPGPYVK | 0.9990 | 2156.1604 | Q01518 |
| SLIALVNDPQPEHPLRADLAEE | 0.9280 | 2514.2502 | P68036 |
| SLIINTFYSNKE | 0.9769 | 1543.7597 | Q76LV2 |
| SLIINTFYSNKE | 0.8679 | 1543.7597 | Q76LV2 |
| SLIINTFYSNKE | 0.8433 | 1543.7597 | Q76LV2 |
| SLLDKFLIK | 0.9482 | 1219.7328 | Q04917 |
| SLLLFEAMRK | 0.9485 | 1322.7168 | P47897 |
| SLPLDTLLVDVEPK | 0.9427 | 1653.8977 | P62314 |
| SNVLIIGELLK | 0.8987 | 1313.7707 | Q92526 |
| SPIMAKPR | 0.8343 | 1014.5432 | Q9NP61 |
| SPLVSRLTLYD | 0.9618 | 1350.6857 | Q32LG3 |
| SPNSKVNTLSKE | 0.9226 | 1446.7466 | P40939 |
| SQCQQPAENK | 0.9376 | 1304.5567 | Q01518 |
| SQLQDTQELLQEENRQK | 0.9995 | 2202.0665 | P35579 |
| SRGFGFVLFKE | 0.9973 | 1401.7117 | Q14103 |
| SSEPACLAEIEEDKAR | 0.9841 | 1919.8683 | P78527 |
| SSFYVNGLTLGGQKCSVIRD | 0.9529 | 2316.1321 | P07737 |
| SSGFSLEDPQTHSNR | 0.8535 | 1748.7502 | P08238 |
| SSMAEVDAAMAARPHSIDGR | 0.9767 | 2158.9636 | P22626 |
| STGALSLKKVPE | 0.8539 | 1372.7714 | P09622 |
| STGLSLEQVKK | 0.9371 | 1332.7401 | P16615 |
| STRIIYGGSVTGATCKE | 0.9962 | 1914.9258 | P60174 |
| STRIIYGGSVTGATCKE | 0.9891 | 1914.9258 | P60174 |
| SVEVDGNSFEASGPSKK | 0.9950 | 1880.8904 | Q12906 |
| SVLISLKQAPLVH | 0.9986 | 1519.8797 | P04973 |
| SVLISLKQAPLVH | 0.9814 | 1519.8797 | P04973 |

| **Identified Peptides (prime sequence)** | **PeptideProphet probability** | **Neutral peptide mass (Da)** | **Exemplary protein ID** |
| --- | --- | --- | --- |
| SVLLPLVAKE | 0.8439 | 1183.6964 | P0C024 |
| SVLVDAFSHVAR | 0.9999 | 1387.6996 | Q9NQG5 |
| SVSLVADENPFAQGALRSE | 0.9978 | 2076.9787 | Q3SX14 |
| SVYIKGFPTDATLDDIKE | 0.9937 | 2155.0837 | P05455 |
| TAEAYLGKK | 0.9108 | 1123.6025 | P11021 |
| TAGIQRIPLPPPPAPE | 0.9454 | 1740.9311 | Q07666 |
| TAHIACK | 0.9547 | 915.4385 | P68104 |
| TALLSSGFSLEDPQTHSNR | 0.9996 | 2147.0031 | P08238 |
| TDINLPYLTMDSSGPK | 0.9237 | 1866.8822 | P38646 |
| TGLAWSKTGPVAKE | 0.9948 | 1587.8409 | Q01518 |
| TGLAWSKTGPVAKE | 0.9515 | 1587.8409 | Q01518 |
| THSLGGGTGSGMGTLLISK | 0.9327 | 1904.9414 | Q13885 |
| THSLGGGTGSGMGTLLISK | 0.8932 | 1888.9465 | Q13885 |
| TILRPLNVEPPLTDLQK | 0.9298 | 2062.1575 | Q99459 |
| TKVVAPTISSPVCQE | 0.9985 | 1730.8661 | Q9Y490 |
| TLHLLPCEVAVDGPAPVGR | 0.9979 | 2088.0574 | Q8TDP1 |
| TLLAKNLPYKVTQDE | 0.9956 | 1876.0094 | P19338 |
| TPAPVEKSPAK | 0.9201 | 1267.6924 | P16401 |
| TPLKPSPLPVIPDTIKE | 0.9133 | 1988.1346 | Q7Z6Z7 |
| TPLLPSTTGLLND | 0.9315 | 1428.7249 | O43175 |
| TPLSKLMKAYCE | 0.9343 | 1583.7839 | P61956 |
| TQDKLYQPEYQEVSTEEQREEISGK | 0.9992 | 3157.4839 | Q9Y4L1 |
| TRKYTLPPGVDPTQVSSSLSPE | 0.9049 | 2474.2441 | P04792 |
| TSIANLPKLNKLKKLE | 0.8991 | 2009.2401 | P39687 |
| TSLYTQDR | 0.9701 | 1070.4781 | P50897 |
| TTAIAEAWAR | 0.8972 | 1176.5675 | Q71U36 |
| TTFNSIMK | 0.9936 | 1056.5062 | Q562R1 |
| TTGLAWSKTGPVAKE | 0.8693 | 1688.8886 | Q01518 |
| TTSAGIMDHEEAR | 0.9998 | 1504.6364 | P62244 |
| TTVHAITATQK | 0.9219 | 1285.6779 | P04406 |
| TVEGPPPKDTGIAR | 0.8885 | 1552.7998 | P14678 |
| VAGLAGKDPVQCSRD | 0.9072 | 1687.8100 | Q99497 |
| VAKLGNREDPLPQDSFE | 0.9355 | 2029.9857 | P57737 |
| VAPISDIIAIK | 0.8800 | 1254.7335 | P13804 |
| VAVLPHILD | 0.9575 | 1063.5814 | Q15084 |
| VENGGSLGSKK | 0.8533 | 1218.6356 | P14618 |
| VEPSDTIENVKAK | 0.8627 | 1572.8147 | P62987 |
| VFFFGTHE | 0.9667 | 1070.4536 | Q9XSK7 |
| VGLLIGPRGNTLKNIE | 0.8945 | 1809.026 | Q15637 |

| **Identified Peptides (prime sequence)** | **PeptideProphet probability** | **Neutral peptide mass (Da)** | **Exemplary protein ID** |
| --- | --- | --- | --- |
| VGMGQKDSYVGDEAQSKR | 0.9979 | 2097.9902 | P62736 |
| VHAITATQK | 0.9656 | 1083.5825 | P04406 |
| VHAITATQKTVD | 0.9856 | 1398.7255 | P04406 |
| VIILNHPGQISAGYAPVLD | 0.9170 | 2064.0717 | P68103 |
| VILIDPFHK | 0.9930 | 1196.6706 | P61313 |
| VIVVSVKEAIPGGKVKKG | 0.9207 | 2007.2534 | A8GPE0 |
| VLAAELLR | 0.9794 | 971.5552 | P78371 |
| VLPKLFE | 0.8768 | 960.5432 | Q14008 |
| VLPNIQAVLLPKKTE | 0.8400 | 1806.0767 | Q96QV6 |
| VLPNIQAVLLPKKTE | 0.9563 | 1806.0767 | Q96QV6 |
| VMVGMGQKDSYVGDEAQSK | 0.9994 | 2171.9980 | P62736 |
| VMVGMGQKDSYVGDEAQSKR | 0.9808 | 2328.0991 | P62736 |
| VNITPAEVGVLVGKDR | 0.9327 | 1781.9788 | P07737 |
| VPIILVGNKK | 0.8591 | 1223.7754 | P61586 |
| VQAFQFTDKHGE | 0.9664 | 1521.7001 | Q06830 |
| VQAFQFTDKHGE | 0.9093 | 1521.7001 | Q06830 |
| VQALDDTERGSGGFGSTGKN | 0.9997 | 2110.9668 | P33316 |
| VQSGSHLAAR | 0.9976 | 1112.5475 | P04040 |
| VQSGSHLAARE | 0.9554 | 1241.5901 | P04040 |
| VSTYIKK | 0.9312 | 981.5647 | P68104 |
| VTIVNILTNR | 0.9849 | 1229.6880 | P07355 |
| VVAVHPGGDTVAIGGVDGNVR | 0.9378 | 2076.0501 | O75083 |
| VVAVLPHILDTGAAGR | 0.9999 | 1675.9158 | Q15084 |
| WIVLKEPISVSSE | 0.8925 | 1601.8453 | P00918 |
| WVAMAPKPGPYVK | 0.9879 | 1586.8431 | Q01518 |
| YGKIDTIEIITDR | 0.9996 | 1651.8569 | P22626 |
| YHQVIQQMEQK | 0.9185 | 1546.7350 | P80303 |
| YQEVSTEEQREEISGK | 0.9983 | 2026.9231 | Q9Y4L1 |
| YSCVGVFQHGKVE | 0.8550 | 1624.7456 | P34931 |
| YSCVGVFQHGKVE | 0.9617 | 1624.7456 | P34931 |
| YVELQKEEAQK | 0.9304 | 1507.7670 | Q00839 |
| YVTIIDAPGHRD | 0.8654 | 1443.6895 | P68104 |
